# Supplementary material for: Pharmacological interventions for addressing pediatric and adolescent obesity: A systematic review and network meta-analysis
Source: PLoS One. 2025 Feb 27;20(2):e0314787. doi: 10.1371/journal.pone.0314787 (PMC11867386; doi:10.1371/journal.pone.0314787)
Supplement: S1 File — (DOC) [file pone.0314787.s001.doc]

**Title:** Pharmacological Interventions for Addressing Pediatric and Adolescent Obesity: A Systematic Review and Network Meta-analysis.

**Authors:** Shuo Yang^1^, Shuangqing Xin^1^, Ronghui Ju^2^, Peizhuo Zang^3^

**Affiliation:** ^1^Department of Endocrinology and Metabolism, The People's Hospital of China Medical University, The People's Hospital of Liaoning Province, Shenyang, China. ^2^Department of Radiology, The People's Hospital of China Medical University, The People’s Hospital of Liaoning Province, Shenyang, China. ^3^Department of Cerebrovascular Disease Treatment Center, The People's Hospital of China Medical University, The People's Hospital of Liaoning Province, Shenyang, China.

**Corresponding author:** Peizhuo Zang, Department of Cerebrovascular Disease Treatment Center, The People's Hospital of China Medical University, The People's Hospital of Liaoning Province, No.33 Wenyi Road, Shenhe District, Shenyang 110016, Liaoning Province, China. Email: [d10@lnph.com;](mailto:d10@lnph.com;) Ronghui Ju, Department of Radiology, The People's Hospital of China Medical University, The People’s Hospital of Liaoning Province, No.33 Wenyi Road, Shenhe District, Shenyang 110016, Liaoning Province, China. Email: d8299@lnph.com.

**Contents of supplementary appendix**

**Appendix 1** 4

Search strategy

**Appendix 2** 10

Deviance information criterion for model selection

**Appendix 3** 11

Characteristics of included studies

**Appendix 4** 16

Characteristics of excluded studies

**Appendix 5** 23

Network plots for outcomes

**Appendix 6** 27

Risk of bias assessment

**Appendix 7** 42

Comparison-adjusted funnel plot

**Appendix 8** 46

Results of pairwise meta-analysis for each outcome

**Appendix 9** 52

Results of network meta-analysis

**Appendix 10** 60

Ranking based on simulations for outcome

**Appendix 11** 62

Subgroup network meta-analyses

**Appendix 12** 65

Sensitivity analyses

**Appendix 13** 66

Meta-regression for network meta-analyses

**Appendix 14** 67

Grading the evidence for the network meta-analysis using CINeMA

**Appendix 15** 104

References for the included trials

**Appendix 1. Search strategy**

**Supplemental Table 1. Search Strategy**

| **Data source** | **Search terms** | **Results** |
| --- | --- | --- |
| PubMed | 1. Obesity[mh] OR ([Obesity, Abdominal](https://www.msu.aliya.topdot.xyz/mesh/68056128)) OR ([Obesity, Maternal](https://www.msu.aliya.topdot.xyz/mesh/2030840)) OR ([Obesity, Metabolically Benign](https://www.msu.aliya.topdot.xyz/mesh/2009700)) OR ([Obesity, Morbid](https://www.msu.aliya.topdot.xyz/mesh/68009767)) OR Overweight[mh] | 277,179 |
|  | 1. Adolescent[mh] OR Adolescents OR Adolescence OR Teens OR Teen OR Teenagers OR Teenager OR Youth OR Youths OR (Adolescents, Female) OR (Adolescent, Female) OR (Female Adolescent) OR (Female Adolescents) OR (Adolescents, Male) OR (Adolescent, Male) OR (Male Adolescent) OR (Male Adolescents) OR Child[mh] OR Children OR kid OR kids OR ([Only Child](https://www.msu.aliya.topdot.xyz/mesh/68009863)) OR [Minors](https://www.msu.aliya.topdot.xyz/mesh/68033242) OR Pediatrics[mh] | 3,805,192 |
|  | 1. (Anti-obesity drugs[mh]) OR (Agents, Anti-Obesity) OR (Anti Obesity Agents) OR (Drugs, Anti-Obesity) OR (Agent, Anti-Obesity) OR (Agent, Antiobesity) OR (Drug, Antiobesity) OR (Weight-Loss Agents) OR (Agents, Weight-Loss) OR (Weight Loss Drugs) OR (Drug, Weight-Loss) | 13,604 |
|  | 1. Metformin[mh] OR Biguanides OR Dimethylguanylguanidine OR Glucophage OR (Metformin Hydrochloride) OR (Hydrochloride, Metformin) OR (Metformin HCl) OR (HCl, Metformin) | 19,597 |
|  | 1. (Glucagon-Like Peptide-1 Receptor Agonists[mh]) OR (glucagon-like peptide 1 receptor inhibitor) OR (glucagon-like peptide 1 receptor agonist) OR (glucagon like peptide 1 inhibitor) OR (glucagon-like peptide 1 agonist) OR (GLP-1 receptor inhibitor) OR (GLP-1 receptor agonist) OR (GLP-1 inhibitor) OR (GLP-1 agonist) OR Albiglutide OR Dulaglutide OR Semaglutide OR Liraglutide OR Lixisenatide OR Taspoglutide OR Exenatide | 11,489 |
|  | 1. Orlistat[mh] OR Tetrahydrolipstatin OR THLP OR Tetrahydrolipastatin OR Xenical OR Cetilistat | 1,549 |
|  | 1. Sibutramine[mh] | 946 |
|  | 1. Fluoxetine[mh] OR Fluoxetin OR Sarafem OR (Fluoxetine Hydrochloride) OR Prozac OR (Lilly 110140) | 10,288 |
|  | 1. Rimonabant[mh] OR (Rimonabant Hydrochloride) OR Acomplia OR SR141716 | 2,301 |
|  | 1. lorcaserin[mh] OR (APD 356) | 267 |
|  | 1. Diethylpropion[mh] OR Amfepramon OR Phepranon OR (2-Diethylaminopropiophenone) OR Lipomin OR Regenon OR (Dietil retard) OR Tenuate OR Propion OR (Hydrochloride, Diethylpropion) | 354 |
|  | 1. Mazindol[mh] OR Mazindole OR Teronac OR Teronak OR AN-448 OR Sanorex | 618 |
|  | 1. Phentermine[mh] OR Duromine OR (Phentermine Hydrochloride) OR Adipex-P OR (Hydrochloride, Phentermine) OR (Adipex P) OR AdipexP OR Ionamine | 1,256 |
|  | 1. Topiramate[mh] OR Epitomax OR (McN 4853) OR McN-4853 OR USL255 OR Topamax | 3,116 |
|  | 1. Randomized Controlled Trial[Publication Type] OR randomized[Title/Abstract] OR placebo[Title/Abstract] | 1,038,214 |
|  | 1. #1 AND #2 | 71,530 |
|  | 1. #3- #14 | 60,481 |
|  | 1. #15 AND #16 AND #17 | 411 |
| Embase | 1. ‘Obesity’/exp | 817,123 |
|  | 1. ‘[Obesity, Abdominal](https://www.msu.aliya.topdot.xyz/mesh/68056128)’ OR ‘[Obesity, Maternal](https://www.msu.aliya.topdot.xyz/mesh/2030840)’ OR ‘[Obesity, Metabolically Benign](https://www.msu.aliya.topdot.xyz/mesh/2009700)’ OR ‘[Obesity, Morbid](https://www.msu.aliya.topdot.xyz/mesh/68009767)’ OR Overweight | 181,304 |
|  | 1. #1 OR #2 | 826,237 |
|  | 1. ‘Adolescent’/exp | 2,086,569 |
|  | 1. Adolescents OR Adolescence OR Teens OR Teen OR Teenagers OR Teenager OR Youth OR Youths OR ‘Adolescents, Female’ OR ‘Adolescent, Female’ OR ‘Female Adolescent’ OR ‘Female Adolescents’ OR ‘Adolescents, Male’ OR ‘Adolescent, Male’ OR ‘Male Adolescent’ OR ‘Male Adolescents’ | 4,605,835 |
|  | 1. ‘Child’/exp | 3,937,292 |
|  | 1. Children OR kid OR kids OR ‘[Only Child](https://www.msu.aliya.topdot.xyz/mesh/68009863)’ OR [Minors](https://www.msu.aliya.topdot.xyz/mesh/68033242) | 4,282,775 |
|  | 1. ‘Pediatrics’/exp | 1,073,544 |
|  | 1. #4- #8 | 5,899,709 |
|  | 1. ‘Anti-obesity drugs’ OR ‘Agents, Anti-Obesity’ OR ‘Anti Obesity Agents’ OR ‘Drugs, Anti-Obesity’ OR ‘Agent, Anti-Obesity’ OR ‘Agent, Antiobesity’ OR ‘Drug, Antiobesity’ OR ‘Weight-Loss Agents’ OR ‘Agents, Weight-Loss’ OR ‘Weight Loss Drugs’ OR ‘Drug, Weight-Loss’ | 23,690 |
|  | 1. ‘Metformin’/exp | 93,057 |
|  | 1. Biguanides OR Dimethylguanylguanidine OR Glucophage OR ‘Metformin Hydrochloride’ OR ‘Hydrochloride, Metformin’ OR ‘Metformin HCl’ OR ‘HCl, Metformin’ | 130,945 |
|  | 1. #11 OR #12 | 134,038 |
|  | 1. ‘glucagon-like peptide-1 receptor agonists’ OR ‘glucagon-like peptide 1 receptor inhibitor’ OR ‘glucagon-like peptide 1 receptor agonist’ OR ‘glucagon like peptide 1 inhibitor’ OR ‘glucagon-like peptide 1 agonist’ OR ‘GLP-1 receptor inhibitor’ OR ‘GLP-1 receptor agonist’ OR ‘GLP-1 inhibitor’ OR ‘GLP-1 agonist’ OR Albiglutide OR Dulaglutide OR Semaglutide OR Liraglutide OR Lixisenatide OR Taspoglutide OR Exenatide | 50,991 |
|  | 1. ‘Tetrahydrolipstatin’/exp | 7,660 |
|  | 1. THLP OR Tetrahydrolipastatin OR Xenical OR Cetilistat | 7,660 |
|  | 1. #15 OR #16 | 7,698 |
|  | 1. ‘Sibutramine’/exp | 4,850 |
|  | 1. ‘Fluoxetine’/exp | 55,101 |
|  | 1. Fluoxetin OR Sarafem OR ‘Fluoxetine Hydrochloride’ OR Prozac OR ‘Lilly 110140’ | 53,641 |
|  | 1. #19 OR #20 | 55,181 |
|  | 1. ‘Rimonabant’/exp | 7,209 |
|  | 1. ‘Rimonabant Hydrochloride’ OR Acomplia OR SR141716 | 7,033 |
|  | 1. #22 OR #23 | 7.249 |
|  | 1. ‘lorcaserin’/exp | 1,526 |
|  | 1. APD 356 | 23 |
|  | 1. #25 OR #26 | 1,540 |
|  | 1. ‘Amfepramone’/exp | 1,738 |
|  | 1. Amfepramon OR Phepranon OR ‘2-Diethylaminopropiophenone’ OR Lipomin OR Regenon OR ‘Dietil retard’ OR Tenuate OR Propion OR ‘Hydrochloride, Diethylpropion’ | 44,616 |
|  | 1. #28 OR #29 | 44,629 |
|  | 1. ‘Mazindol’/exp | 2,056 |
|  | 1. Mazindole OR Teronac OR Teronak OR ‘AN-448’ OR Sanorex | 1,907 |
|  | 1. #31 OR #32 | 2,062 |
|  | 1. ‘Phentermine’/exp | 4,103 |
|  | 1. Duromine OR ‘Phentermine Hydrochloride’ OR ‘Adipex-P’ OR ‘Hydrochloride, Phentermine’ OR ‘Adipex P’ OR AdipexP OR Ionamine | 3,353 |
|  | 1. #34 OR #35 | 4,105 |
|  | 1. ‘Topiramate’/exp | 28,234 |
|  | 1. Epitomax OR ‘McN 4853’ OR ‘McN-4853’ OR USL255 OR Topamax | 26,907 |
|  | 1. #37 OR #38 | 28,238 |
|  | 1. #10 OR #11 OR #13 OR #14 OR #17 OR #18 OR #21 OR #24 OR #27 OR #30 OR #33 OR #36 OR #39 | 311,547 |
|  | 1. 'randomized controlled trial'/exp | 1,083,260 |
|  | 1. 'controlled clinical trial' OR 'randomized controlled trial' OR rct OR random* OR trial:ab,ti | 2,943,759 |
|  | 1. #41 OR #42 | 2,943,759 |
|  | 1. #3 AND #9 AND #40 AND #43 | 1,314 |
| Central | 1. Obesity [MeSH] | 21,604 |
|  | 1. Overweight [MeSH] | 25,056 |
|  | 1. (‘[Obesity, Abdominal](https://www.msu.aliya.topdot.xyz/mesh/68056128)’ OR ‘[Obesity, Maternal](https://www.msu.aliya.topdot.xyz/mesh/2030840)’ OR ‘[Obesity, Metabolically Benign](https://www.msu.aliya.topdot.xyz/mesh/2009700)’ OR ‘[Obesity, Morbid](https://www.msu.aliya.topdot.xyz/mesh/68009767)’):ti,ab,kw | 10,833 |
|  | 1. #1 OR #2 OR #3 | 30,998 |
|  | 1. Adolescent [MeSH] | 126,194 |
|  | 1. (Adolescents OR Adolescence OR Teens OR Teen OR Teenagers OR Teenager OR Youth OR Youths OR ‘Adolescents, Female’ OR ‘Adolescent, Female’ OR ‘Female Adolescent’ OR ‘Female Adolescents’ OR ‘Adolescents, Male’ OR ‘Adolescent, Male’ OR ‘Male Adolescent’ OR ‘Male Adolescents’):ti,ab,kw | 165,987 |
|  | 1. Child [MeSH] | 78,984 |
|  | 1. (Children OR kid OR kids OR ‘[Only Child](https://www.msu.aliya.topdot.xyz/mesh/68009863)’ OR [Minors](https://www.msu.aliya.topdot.xyz/mesh/68033242)):ti,ab,kw | 205,653 |
|  | 1. Pediatrics [MeSH] | 1,179 |
|  | 1. #5 OR #6 OR #7 OR #8 OR #9 | 310,878 |
|  | 1. (‘Anti-obesity drugs’ OR ‘Agents, Anti-Obesity’ OR ‘Anti Obesity Agents’ OR ‘Drugs, Anti-Obesity’ OR ‘Agent, Anti-Obesity’ OR ‘Agent, Antiobesity’ OR ‘Drug, Antiobesity’ OR ‘Weight-Loss Agents’ OR ‘Agents, Weight-Loss’ OR ‘Weight Loss Drugs’ OR ‘Drug, Weight-Loss’):ti,ab,kw | 12,563 |
|  | 1. Metformin [MeSH] | 5,011 |
|  | 1. (Biguanides OR Dimethylguanylguanidine OR Glucophage OR ‘Metformin Hydrochloride’ OR ‘Hydrochloride, Metformin’ OR ‘Metformin HCl’ OR ‘HCl, Metformin’):ti,ab,kw | 1,371 |
|  | 1. #12 OR #13 | 5,990 |
|  | 1. (‘glucagon-like peptide-1 receptor agonists’ OR ‘glucagon-like peptide 1 receptor inhibitor’ OR ‘glucagon-like peptide 1 receptor agonist’ OR ‘glucagon like peptide 1 inhibitor’ OR ‘glucagon-like peptide 1 agonist’ OR ‘GLP-1 receptor inhibitor’ OR ‘GLP-1 receptor agonist’ OR ‘GLP-1 inhibitor’ OR ‘GLP-1 agonist’ OR Albiglutide OR Dulaglutide OR Semaglutide OR Liraglutide OR Lixisenatide OR Taspoglutide OR Exenatide):ti,ab,kw | 6,482 |
|  | 1. Orlistat [MeSH] | 330 |
|  | 1. (Tetrahydrolipstatin OR THLP OR Tetrahydrolipastatin OR Xenical OR Cetilistat):ti,ab,kw | 144 |
|  | 1. #16 OR #17 | 406 |
|  | 1. (Sibutramine):ti,ab,kw | 358 |
|  | 1. Fluoxetine [MeSH] | 1,634 |
|  | 1. (Fluoxetin OR Sarafem OR ‘Fluoxetine Hydrochloride’ OR Prozac OR ‘Lilly 110140’):ti,ab,kw | 3,827 |
|  | 1. #20 OR #21 | 3,827 |
|  | 1. Rimonabant [MeSH] | 127 |
|  | 1. (‘Rimonabant Hydrochloride’ OR Acomplia OR SR141716):ti,ab,kw | 40 |
|  | 1. #23 OR #24 | 157 |
|  | 1. (‘lorcaserin’ OR APD 356):ti,ab,kw | 150 |
|  | 1. Diethylpropion [MeSH] | 51 |
|  | 1. (Amfepramon OR Phepranon OR Lipomin OR Regenon OR ‘Dietil retard’ OR Tenuate OR Propion OR ‘Hydrochloride, Diethylpropion’):ti,ab,kw | 5,795 |
|  | 1. #27 OR #28 | 5,827 |
|  | 1. Mazindol [MeSH] | 56 |
|  | 1. (Mazindole OR Teronac OR Teronak OR ‘AN-448’ OR Sanorex):ti,ab,kw | 1,207 |
|  | 1. #30 OR #31 | 1,207 |
|  | 1. Phentermine [MeSH] | 136 |
|  | 1. (Duromine OR ‘Phentermine Hydrochloride’ OR ‘Adipex-P’ OR ‘Hydrochloride, Phentermine’ OR ‘Adipex P’ OR AdipexP OR Ionamine):ti,ab,kw | 17 |
|  | 1. #33 OR #34 | 145 |
|  | 1. Topiramate [MeSH] | 655 |
|  | 1. (Epitomax OR ‘McN 4853’ OR ‘McN-4853’ OR USL255 OR Topamax):ti,ab,kw | 77 |
|  | 1. #36 OR #37 | 704 |
|  | 1. #11 OR #14 OR #15 OR #18 OR #19 OR #22 OR #25 OR #26 OR #29 OR #32 OR #35 OR #38 | 34,418 |
|  | 1. #4 AND #10 AND #39 | 799 |
| Clinicaltrials.gov | Study types: Interventional studies  Study results: All studies  Conditions: Obesity  Age: Child (birth - 17)  Intervention/treatment: Metformin | 47 |
|  | Study types: Interventional studies  Study results: All studies  Conditions: Obesity  Age: Child (birth - 17)  Intervention/treatment: Albiglutide | 0 |
|  | Study types: Interventional studies  Study results: All studies  Conditions: Obesity  Age: Child (birth - 17)  Intervention/treatment: Dulaglutide | 1 |
|  | Study types: Interventional studies  Study results: All studies  Conditions: Obesity  Age: Child (birth - 17)  Intervention/treatment: Semaglutide | 12 |
|  | Study types: Interventional studies  Study results: All studies  Conditions: Obesity  Age: Child (birth - 17)  Intervention/treatment: Liraglutide | 10 |
|  | Study types: Interventional studies  Study results: All studies  Conditions: Obesity  Age: Child (birth - 17)  Intervention/treatment: Lixisenatide | 0 |
|  | Study types: Interventional studies  Study results: All studies  Conditions: Obesity  Age: Child (birth - 17)  Intervention/treatment: Taspoglutide | 0 |
|  | Study types: Interventional studies  Study results: All studies  Conditions: Obesity  Age: Child (birth - 17)  Intervention/treatment: Exenatide | 10 |
|  | Study types: Interventional studies  Study results: All studies  Conditions: Obesity  Age: Child (birth - 17)  Intervention/treatment: Orlistat | 4 |
|  | Study types: Interventional studies  Study results: All studies  Conditions: Obesity  Age: Child (birth - 17)  Intervention/treatment: Sibutramine | 3 |
|  | Study types: Interventional studies  Study results: All studies  Conditions: Obesity  Age: Child (birth - 17)  Intervention/treatment: Fluoxetine | 1 |
|  | Study types: Interventional studies  Study results: All studies  Conditions: Obesity  Age: Child (birth - 17)  Intervention/treatment: Rimonabant | 0 |
|  | Study types: Interventional studies  Study results: All studies  Conditions: Obesity  Age: Child (birth - 17)  Intervention/treatment: lorcaserin | 3 |
|  | Study types: Interventional studies  Study results: All studies  Conditions: Obesity  Age: Child (birth - 17)  Intervention/treatment: Diethylpropion | 1 |
|  | Study types: Interventional studies  Study results: All studies  Conditions: Obesity  Age: Child (birth - 17)  Intervention/treatment: Mazindol | 0 |
|  | Study types: Interventional studies  Study results: All studies  Conditions: Obesity  Age: Child (birth - 17)  Intervention/treatment: Phentermine | 10 |
|  | Study types: Interventional studies  Study results: All studies  Conditions: Obesity  Age: Child (birth - 17)  Intervention/treatment: Topiramate | 13 |

**Appendix 2. Deviance information criterion for model selection**

**Supplemental Table 2**. Deviance information criterion for model selection (fixed- vs. random-effects)

| **Outcome** | **Model** | **Deviance Information**  **Criterion (DIC)** | **Accepted Model** |
| --- | --- | --- | --- |
| BMI | Fixed | 260.91 | Random |
|  | Random | 126.94 |  |
| Percent change in BMI | Fixed | 37.92 | Random |
|  | Random | 35.62 |  |
| BMI-SDS | Fixed | 23.97 | Fixed |
|  | Random | 25.01 |  |
| Weight | Fixed | 113.34 | Random |
|  | Random | 83.10 |  |
| Waist circumference | Fixed | 67.43 | Random |
|  | Random | 67.67 |  |
| TC | Fixed | 42.50 | Fixed |
|  | Random | 44.46 |  |
| LDL-C | Fixed | 55.54 | Random |
|  | Random | 56.83 |  |
| HDL-C | Fixed | 54.86 | Random |
|  | Random | 55.77 |  |
| TG | Fixed | 91.13 | Random |
|  | Random | 68.07 |  |
| FBG | Fixed | 55.74 | Fixed |
|  | Random | 57.36 |  |
| FINS | Fixed | 77.95 | Random |
|  | Random | 55.89 |  |
| HOMA-IR | Fixed | 63.54 | Random |
|  | Random | 50.19 |  |
| SBP | Fixed | 54.83 | Fixed |
|  | Random | 56.68 |  |
| DBP | Fixed | 66.51 | Random |
|  | Random | 66.15 |  |
| Heart rate | Fixed | 44.11 | Random |
|  | Random | 41.86 |  |
| Gastrointestinal disorders | Fixed | 28.34 | Random |
|  | Random | 26.70 |  |
| Depression | Fixed | 23.13 | Random |
|  | Random | 23.22 |  |
| SAE | Fixed | 38.47 | Random |
|  | Random | 38.36 |  |

**Appendix 3.Characteristics of included studies**

**Supplemental Table 3.** Baseline characteristics

| **Trial** | **Publication**  **status**  **Registration** | **Phase** | **Total randomised** | **Drug** | **Comparator** | **Mean Age**  **(year)** | **Race (Primary %)** | **Male (%)** | **Weight (kg)** | **Height**  **(cm)** | **BMI**  **(kg/m^2^)** | **Waist circumference (cm)** | **Follow-up (weeks)** | **Outcome**s reported |
| --- | --- | --- | --- | --- | --- | --- | --- | --- | --- | --- | --- | --- | --- | --- |
| Kelly 2022^1^ | Published  NCT03922945 | Phase 4 | 223 | PHEN/TPM (7.5-15 mg/  46-92 mg) | Placebo | 14.0±1.35 | White (66.8) | 45.7 | 106.1±23.7 | 167.1±7.8 | 37.8±7.1 | 114.0±15.9 | 56 | A, B, D, E, M, N, O, P, Q, R |
| Weghuber 2022^2^ | Published  NCT04102189 | Phase 3 | 201 | Semaglutide (2.4mg) | Placebo | 15.4±1.6 | white (79.0) | 38.0 | 107.5±24.5 | 169.7±9.8 | 37.0±6.4 | 110.4±16.0 | 68 | A, B, C, D, E, J, M, N, O, P, Q ,R |
| Weghuber 2020^3^ | [Published](http://clinicaltrials.gov/show/NCT1874431)  [NCT02794402](http://clinicaltrials.gov/show/NCT1874431) | Phase 2 | 44 | Exenatide XR (2.0mg) | Placebo | 14.0±2.33 | White  (93.2) | 50.0 | 104.4±22.1 | 169.3±9.9 | 36.1±4.8 | 113.9±11.1 | 26 | A, C, D, E, F, G, H, I, J, K, M, N, P, R |
| Kelly 2013^4^ | Published  NCT01237197 | Phase 2 | 26 | Exenatide (10-20mg) | Placebo | 15.2 ±1.8 | White  (76.9) | 38.5 | 124.0±19.3 | NR | 42.5 ±6.81 | 130 ±14.1 | 12 | A, B, D, E, F, G, H, I, J, K, M, N, O, R |
| Fox 2022^5^ | Published  NCT02496611 | Phase 2 | 66 | Exenatide XR (2.0mg) | Placebo | 16.0±1.5 | White  (82.0) | 53.0 | 108.5 ±17.6 | 171.2 ±8.2 | 36.9±4.4 | NR | 52 | A, B, D, F, G, H, I, J, K, M, N, O, P, R |
| Kelly 2020^6^ | Published  NCT02918279 | Phase 3 | 251 | Liraglutide  (3.0 mg) | Placebo | 14.5±1.6 | White  (87.6) | 40.6 | 100.8±20.7 | 170.0 ±10.0 | 35.6±5.4 | 105.9±13.1 | 56 | A, B, C, D, E, J, M, N, O, P, Q, R |
| Fox 2016^7^ | Published  NCT01859013 | Phase 2 | 30 | Topiramate  (75 mg) | Placebo | 15.2±1.7 | White  (60.0) | 36.7 | 115.0±19.9 | 168.0 ±8.0 | 40.3±4.6 | NR | 24 | A, B, D, F, G, H, I, J, K, M, N, O, Q, R |
| Atabek 2008^8^ | Published | NR | 120 | Metformin (1000mg) | Placebo | 11.8±2.8 | NR | 50.0 | 66.9±16.8 | 152.3±12.1 | 28.4±3.4 | NR | 24 | A, D, F, G, H, I, J, K, L, M, N |
| Clarson 2009^9^ | Published | NR | 31 | Metformin (1500mg) | No treatment | 13.1 | NR | NR | NR | NR | 35.0±1.90 | NR | 24 | A, G, H, I, J, K, L |
| Freemark 2007^10^ | Published | NR | 32 | Metformin (1000mg) | Placebo | 14.9±0.7 | Caucasian  (55.2) | 62.1 | NR | NR | 40.1±1.8 | NR | 24 | A^a^ |
| Kendall 2013^11^ | Published  ISRCTN19517475 | NR | 151 | Metformin (1500mg) | Placebo | 13.7±2.2 | White (76.2) | 32.5 | 98.3±23.0 | 163.5 ±11.0 | 36.5±6.34 | NR | 24 | A, C, D, F, G, H, I, J, K, L, M, N, R |
| Mauras 2012^12^ | Published  NCT00139477 | NR | 66 | Metformin (1000-2000mg) | No treatment | 12.2±2.7 | White (45.5) | 45.5 | NR | 156.2±14.0 | 32.6 ±5.07 | 101.4 ±12.0 | 24 | A^a^, D^a^, R |
| Rezvanian 2010^13^ | Published | NR | 180 | Metformin (1500mg), Fluoxetine (20mg),  Metformin (1500mg)+Fluoxetine (20mg) | Placebo | 13.4±1.3 | NR | NR | NR | NR | 26.4 ±0.67 | 81.8 ±2.9 | 24 | A, E, R |
| Wiegand 2010^14^ | Published | NR | 70 | Metformin (1000 mg) | Placebo | 15.05 | Caucasian(88.6) | 32.9 | NR | NR | 34.8 ±5.4 | NR | 24 | A, C, F, G, H, I, J, K, L, M, N, P |
| Wilson 2010^15^ | Published  NCT00209482, NCT00120146 | Phase 2  Phase 3 | 77 | Metformin XR (2000 mg) | Placebo | 14.9 ±1.4 | White  (63.6) | 33.8 | 98.8 ±16.3 | NR | 35.9 ±5.2 | 104.3 ±11.2 | 48 | A, G, H, I, L, R |
| Yanovski 2011^16^ | Published  NCT00005669 | Phase 2 | 100 | Metformin (2000 mg) | Placebo | 10.2 ±1.5 | White  (91.0) | 40.0 | 78.1 ±21.9 | NR | 34.4 ±6.5 | 105.7 ±14.6 | 24 | A, C, D, E, F, G, H, I, J, K, L, M, N, R |
| Pastor-Villaescusa 2016^17^ | Published  EudraCT, ID: 2010-023061-21 | NR | 160 | Metformin (1000 mg) | Placebo | 6.8 to 15.3 | NR | 51.4 | 68.3 ±17.6 | 150.4 ±13.2 | 29.4 ±3.6 | 93.2 ±12.0 | 24 | A, D, F, G, H, I, J, K, L, R |
| Warnakulasuriya 2018^18^ | Published  NCT02274948 | Phase 4 | 339 | Metformin (1000-2000 mg) | Placebo | 11.94 ±2.31 | NR | 52.2 | NR | 150.8 ±11.8^b^ | 27.7 ±3.4 | 87.4 ±9.2^b^ | 48 | A, C, D, E, F, G, H, I, J, K, L, M, N, P, R |
| Burgert 2008^19^ | Published | NR | 34 | Metformin (1500 mg) | Placebo | 15.0 ±1.59 | White  (57.1) | 32.1 | 110.8 ±17.6 | 165.7 ±8.4 | 40.5 ±5.9 | NR | 16 | A, D, F, G, H, I, J, K, L, M, N, O |
| Garibay-Nieto 2017^20^ | Published  NCT02063802 | Phase 2 | 54 | Metformin (1000 mg) | Placebo | 12.07 ±2.43 | NR | NR | 67.0 ±13.1 | 151.1 ±11.3 | 28.7 ±2.8 | 88.4 ±8.0 | 16 | A, D, E, H, I, K, L |
| Evia-Viscarra 2012^21^ | Published  NCT01410604 | Phase 4 | 31 | Metformin (1000 mg) | Placebo | 13.37 ±1.92 | NR | 29.0 | 83.9 ±22.7 | 158.3 ±10.2 | 33.1 ±6.0 | 103.0 ±15.0 | 12 | A, D, E, J, K, L, R |
| Berkowitz 2003^22^ | Published  NCT00212173 | NR | 82 | Sibutramine (15 mg) | Placebo | 14.1 ±1.2 | White  (54.9) | 32.9 | 103.6 ±15.4 | 165.4 ±8.0 | 37.8 ±3.8 | 110.8 ±10.0 | 24 | B, D, E, M, N, O |
| Berkowitz 2006^23^ | Published  NCT00261911 | Phase 3 | 498 | Sibutramine (10-15 mg) | Placebo | 13.67 ±1.30 | White  (56.6) | 35.3 | 97.9 ±14.7 | 164.5 ±7.7 | 36.1 ±3.9 | 105.9 ±10.3 | 52 | A, B, D, E, H, I, K, L, M, N, O, Q, R |
| García-Morales 2006^24^ | Published | NR | 51 | Sibutramine (10 mg) | Placebo | 14.95±1.22 | NR | 43.5 | 95.8±19.1 | 162.9±8.7 | 35.9 ±5.3 | 109.9±14.9 | 24 | A, B, D, E, F, G, J, M, N, O, R |
| Van Mil 2007^25^ | Published | NR | 24 | Sibutramine (10 mg) | Placebo | 13.95 ±1.26 | NR | 45.8 | 85.0 ±16.2 | 163.0±6.5 | 31.7 ±4.9 | NR | 12 | A, C, D, M, N, O, Q |
| Chanoine 2005^26^ | Published | NR | 539 | Orlistat  (360 mg) | Placebo | 13.57 ±1.27 | White  (76.0) | 33.0 | 96.8 ±14.8 | 164.7 ±8.2 | 35.6 ±4.2 | 105.8 ±11.0 | 52 | A^a^, Q, R^a^ |
| Maahs 2006^27^ | Published | NR | 40 | Orlistat  (360 mg) | Placebo | 15.8 ±1.4 | NR | 32.5 | 112.7 ±31.3 | NR | 40.5 ± 9.1 | NR | 24 | A, D, F, G, H, I, R^a^ |
| NCT00001723^28^ | NCT00001723 | Phase 2 | 200 | Orlistat  (360 mg) | Placebo | 14.59 ±1.41 | NR | 34.5 | NR | NR | NR | NR | 24 | A, D, R |
| Ozkan 2004^29^ | Published | NR | 42 | Orlistat  (360 mg) | No treatment | 12.7 ±2.3 | NR | 33.0 | 78.2 ±18.7 | NR | 31.88 | NR | 47 | A, D, P |
| Zahmatkesh 2023^30^ | Published  IRCT20220409054467N2 | NR | 60 | Orlistat  (360 mg) | Placebo | 13.8 ±1.80 | NR | 56.6 | 61.5±12.5 | 152.0±10.8 | 26.9 ±2.1 | 90.1±13.1 | 12 | A, E, F, G, H, I, J, K, L |

^a^The information came from a meta-analysis and wasn’t in the original paper

^b^The data came from 150 participants in the analysis

Abbreviations: A, BMI; B, change in BMI; C, BMI-SDS; D, weight; E, waist circumference; F, TC; G, LDL-C; H, HDL-C; I, TG; J, FBG; K, FINS; L, HOMA-IR; M, SBP; N, DBP; O, heart rate; P, gastrointestinal disorders; Q, depression; R, serious adverse events; XR, extended release; NR, not reported

**Appendix 4. Characteristics of excluded studies**

**Supplemental Table 4.Characteristics of excluded studies**

Owing to its volume, we were unable to describe all excluded papers. However, we present representative excluded papers herein

| Study | Title | Reason for exclusion |
| --- | --- | --- |
| Allison 2023 | A pilot randomized controlled trial of liraglutide 3.0 mg for binge eating disorder | Participants were adults |
| Nikolic 2022 | Liraglutide Improved Cardiometabolic Parameters More in Obese than in Non-obese Patients with Type 2 Diabetes: A Real-World 18-Month Prospective Study | Participants were adults |
| Kadowaki 2022 | Semaglutide once a week in adults with overweight or obesity, with or without type 2 diabetes in an east Asian population (STEP 6): a randomised, double-blind, double-dummy, placebo-controlled, phase 3a trial | Participants were adults |
| Garvey 2022 | Two-year effects of semaglutide in adults with overweight or obesity: the STEP 5 trial | Participants were adults |
| Suliman 2021 | Liraglutide 3 mg for the Treatment of Obesity: Real Life Experience of Use in a Large Emirati Population | Participants were adults |
| Tronieri 2020 | Effects of liraglutide on appetite, food preoccupation, and food liking: results of a randomized controlled trial | Participants were adults |
| Tan 2017 | Efficacy and safety of once-weekly semaglutide for the treatment of type 2 diabetes | Participants were adults |
| Al-Tahami 2017 | Metabolic and Inflammatory Changes with Orlistat and Sibutramine Treatment in Obese Malaysian Subjects | Participants were adults |
| Andelman 1967 | Treatment of obesity in underprivileged adolescents. Comparison of diethylpropion hydrochloride with placebo in a double-blind study | Follow-up<12 weeks |
| Grugni 1997 | Dexfentluramine in the treatment of juvenile obesity | Follow-up<12 weeks |
| Casteels 2010 | Metformin therapy to reduce weight gain and visceral adiposity in children and adolescents with neurogenic or myogenic motor deficit | Failure to meet inclusion critia (Children had neurogenic or myogenic motor deficit) |
| Fox 2015 | Topiramate for weight reduction in adolescents with severe obesity | Non-randomized controlled trial (RCT) study |
| Danielsson 2007 | Impact sibutramine therapy in children with hypothalamic obesity or obesity with aggravating syndromes | Secondary cause of obesity (Aim was to treat hypothalamic obesity ) |
| Freemark 2007 | Pharmacotherapy of childhood obesity: An evidence-based, conceptual approach | Non-randomized controlled trial (RCT) study |
| Pan 2022 | Effects of Semaglutide on Cardiac Protein Expression and Cardiac Function of Obese Mice | Ineligible study design (animal experiment) |
| NCT05574439 | Young Adults With Early-onset Obesity Treated With Semaglutide | Participants were adults |
| Bobot 2022 | Liraglutide as a rapidly effective treatment option for refractory severe obesity in adolescents? | Review or meta-analysis |
| Stenlid 2023 | Exenatide decreases circulating dipeptidyl peptidase-4 in adolescents with obesity | Conference abstract |
| Weghuber 2022 | Once-Weekly Subcutaneous Semaglutide 2.4 mg in Adolescents With Overweight or Obesity | Conference abstract |
| Stenlid 2022 | Six-months of treatment with exenatide improves glycemic control, but does not affect endogenous intact GLP-1 concentrations in adolescents with obesity | Conference abstract |
| Williams 2021 | Liraglutide therapy in combination with dietary and lifestyle measures contributing to rapid weight loss in an adolescent with morbid obesity and life threatening obstructive sleep apnoea | Conference abstract |
| Moore 2021 | Phentermine/topiramate after adolescent bariatric surgery shows feasibility and initial efficacy | Conference abstract |
| Berman 2021 | Topiramate for weight management in children <12 with severe obesity | Conference abstract |
| Zhang 2023 | Effects of Orlistat combined with an anti-androgenic oral contraceptive pill on cardiovascular risk factors in overweight or obese PCOS patients | Failure to meet inclusion critia |
| Yoon 2023 | Pharmacokinetic and pharmacodynamic interaction of DWP16001, a sodium-glucose cotransporter-2 inhibitor, with phentermine in healthy subjects | Failure to meet inclusion critia |
| Stenlid 2023 | Screening for Inflammatory Markers Identifies IL-18Rα as a Potential Link between Exenatide and Its Anti-Inflammatory Effect: New Results from the Combat-JUDO Randomized Controlled Trial | Failure to meet inclusion critia |
| Min 2022 | Effect of orlistat during individualized comprehensive life-style intervention on visceral fat in overweight or obese PCOS patients | Failure to meet inclusion critia |
| Holmback 2022 | Effects of a novel weight-loss combination product containing orlistat and acarbose on obesity: a randomized, placebo-controlled trial | Failure to meet inclusion critia |
| Gu 2022 | Effect on the cardiovascular independent risk factor lipoprotein(a) in overweight or obese PCOS patients with ethinyl-estradiol/drospirenone alone or plus orlistat | Failure to meet inclusion critia |
| Fatima 2022 | Phase II Open-label Pilot Trial Of Liraglutide In Adolescents With Obesity After Vertical Sleeve Gastrectomy: Interim Results From 8-weeks Of Treatment | Failure to meet inclusion critia |
| Wang 2021 | Effect of Orlistat on Live Birth Rate in Overweight or Obese Women Undergoing IVF-ET: A Randomized Clinical Trial | Failure to meet inclusion critia |
| NCT05067621 | Liraglutide Effects in Obese Youth With Prediabetes/New Onset Type 2 Diabetes and Non-Alcoholic Fatty Liver Disease | Failure to meet inclusion critia |
| Lofton 2021 | A randomized, double-blind, placebo-controlled trial using liraglutide for weight regain after RYGB | Failure to meet inclusion critia |
| Grudén 2021 | Safety of a Novel Weight Loss Combination Product Containing Orlistat and Acarbose | Failure to meet inclusion critia |
| Tronieri 2020 | Measures of adherence as predictors of early and total weight loss with intensive behavioral therapy for obesity combined with liraglutide 3.0mg | Failure to meet inclusion critia |
| Vallé-Jones 1983 | A comparative study of phentermine and diethylpropion in the treatment of obese patients in general practice | Failure to meet inclusion critia |
| Haqq 2005 | The Study of the Effects of Diet on Metabolism and Nutrition (STEDMAN) weight loss project: rationale and design | Failure to meet inclusion critia |
| Doggrell 2009 | Tesofensine--a novel potent weight loss medicine. Evaluation of: Astrup A, Breum L, Jensen TJ, Kroustrup JP, Larsen TM. Effect of tesofensine on bodyweight loss, body composition, and quality of life in obese patients: a randomised, double-blind, placebo-controlled trial. Lancet 2008;372:1906-13 | Failure to meet inclusion critia |
| Bjorner 2023 | The improved health utility of once-weekly subcutaneous semaglutide 2.4 mg compared with placebo in the STEP 1-4 obesity trials | Older version/duplicate articles of the same study |
| Diez-Lopez 2022 | Liraglutide SC in the treatment of severe obesity in Pediatrics: a missed therapeutic opportunity? | Older version/duplicate articles of the same study |
| Bensignor 2023 | Evaluating potential predictors of weight loss response to liraglutide in adolescents with obesity: a post hoc analysis of the randomized, placebo-controlled SCALE Teens trial | Older version/duplicate articles of the same study |
| Jensterle 2023 | Semaglutide alters tongue transcriptome along with the improvement of taste perception and increased brain activation in response to sweet tasting solution women with obesity and PCOS | Failure to meet inclusion critia (irrelevant research) |
| Elbarbary  2023 | THE EFFICACY AND SAFETY OF LIRAGLUTIDE 3.0 MG FOR WEIGHT MANAGEMENT AS AN ADD-ON TREATMENT IN OBESE ADOLESCENTS WITH TYPE 1 DIABETES | Failure to meet inclusion critia (irrelevant research) |
| Diene 2023 | Liraglutide for Weight Management in Children and Adolescents With Prader-Willi Syndrome and Obesity | Failure to meet inclusion critia (irrelevant research) |
| Aguilar 2023 | Liraglutide in Prader Willi-Syndrome: The Importance of Placebo-Controlled Studies | Failure to meet inclusion critia (irrelevant research) |
| Yu 2022 | Effects of liraglutide or lifestyle interventions combined with other antidiabetic drugs on abdominal fat distribution in people with obesity and type 2 diabetes mellitus evaluated by the energy spectrum ct: a prospective randomized controlled study | Failure to meet inclusion critia (irrelevant research) |
| Uhrenholt 2022 | Effect of Semaglutide versus placebo on psychotic symptoms and quality of life - a pre-specified secondary analysis of HISTORI: A randomized clinical trial in people with pre-diabetes and schizophrenia | Failure to meet inclusion critia (irrelevant research) |
| Fatima 2022 | Interim Results From 16 Weeks of Liraglutide in Teens With Obesity Post Vertical Sleeve Gastrectomy | Failure to meet inclusion critia (irrelevant research) |
| Elbarbary 2022 | Clinical effectiveness of Liraglutide 3.0 mg for weight management as an adjuvant therapy in obese adolescents with type 1 diabetes: A single-center pilot study | Failure to meet inclusion critia (irrelevant research) |
| Diene 2022 | Liraglutide for Weight Management in Children and Adolescents With Prader-Willi Syndrome and Obesity | Failure to meet inclusion critia (irrelevant research) |
| Brady 2022 | Liraglutide does not provide sustainable results for weight improvement in adolescents with obesity | Failure to meet inclusion critia (irrelevant research) |
| Jourdren 2021 | Effect of Semaglutide on body weight in obese children with craniopharyngioma: A preliminary report | Failure to meet inclusion critia (irrelevant research) |
| Jensterle 2021 | Semaglutide reduces fat accumulation in the tongue: A randomized single-blind, pilot study | Failure to meet inclusion critia (irrelevant research) |
| Newsome  2019 | Effect of semaglutide on liver enzymes and markers of inflammation in subjects with type 2 diabetes and/or obesity | Failure to meet inclusion critia (irrelevant research) |
| Jensterle  2021 | Does intervention with GLP-1 receptor agonist semaglutide modulate perception of sweet taste in women with obesity: study protocol of a randomized, single-blinded, placebo-controlled clinical trial | Failure to meet inclusion critia (irrelevant research) |
| Jensen 2018 | Pharmacokinetics and tolerability of semaglutide in people with hepatic impairment | Failure to meet inclusion critia (irrelevant research) |
| Klein 2014 | Liraglutide's safety, tolerability, pharmacokinetics, and pharmacodynamics in pediatric type 2 diabetes: a randomized, double-blind, placebo-controlled trial | No relevant outcomes reported |
| Kelly 2012 | Exenatide as a weight-loss therapy in extreme pediatric obesity: a randomized, controlled pilot study | No relevant outcomes reported |
| Srinivasan 2006 | Randomized, controlled trial of metformin for obesity and insulin resistance in children and adolescents: improvement in body composition and fasting insulin | No relevant outcomes reported |
| Chanoine 2005 | Effect of orlistat on weight and body composition in obese adolescents: a randomized controlled trial | No relevant outcomes reported |
| NCT04102189 | A Research Study on How Well Semaglutide Works in Adolescents With Overweight or Obesity | Study protocol |
| Bhattacharyya 2022 | EFFICACY AND SAFETY OF LIRAGLUTIDE IN AN INDIAN ADOLESCENT POPULATION WITH T2DM AND OBESITY: A SINGLE CENTRE EXPERIENCE FROM EASTERN INDIA | Failure to meet inclusion critia (aim was to treat obesity combined with type 2 diabetes) |
| Arslanian 2022 | Once-Weekly Dulaglutide for the Treatment of Youths with Type 2 Diabetes | Failure to meet inclusion critia (aim was to treat obesity combined with type 2 diabetes) |
| Al-Abdulsalam 2022 | Maturity-onset diabetes of the Young type 7 treated with the glucagon-like peptide-1 receptor agonist (Liraglutide)-First report | Failure to meet inclusion critia (aim was to treat obesity combined with type 2 diabetes) |
| NCT04881799 | Liraglutide in Adolescents With Type 2 Diabetes and Obesity | Failure to meet inclusion critia (aim was to treat obesity combined with type 2 diabetes) |
| NCT04829903 | Dulaglutide Versus Liraglutide in Obese Type 2 Diabetic Adolescents Using Metformin | Failure to meet inclusion critia (aim was to treat obesity combined with type 2 diabetes) |
| Bensignor 2021 | Effect of liraglutide treatment on body mass index and weight parameters in children and adolescents with type 2 diabetes: Post hoc analysis of the ellipse trial | Failure to meet inclusion critia (aim was to treat obesity combined with type 2 diabetes) |
| Oberle 2020 | Effect of liraglutide treatment on weight parameters in children and adolescents with type 2 diabetes: Post hoc analysis of the ellipse trial | Failure to meet inclusion critia (aim was to treat obesity combined with type 2 diabetes) |
| Yang 2018 | Efficacy and safety of lixisenatide in a predominantly Asian population with type 2 diabetes insufficiently controlled with basal insulin: The GetGoal-L-C randomized trial | Failure to meet inclusion critia (aim was to treat obesity combined with type 2 diabetes) |
| Skrivanek 2014 | Dose-finding results in an adaptive, seamless, randomized trial of once-weekly dulaglutide combined with metformin in type 2 diabetes patients (AWARD-5) | Failure to meet inclusion critia (aim was to treat obesity combined with type 2 diabetes) |
| Hollander 2013 | Effects of taspoglutide on glycemic control and body weight in obese patients with type 2 diabetes (T-emerge 7 study) | Failure to meet inclusion critia (aim was to treat obesity combined with type 2 diabetes) |
| Klein 2014 | Liraglutide's safety, tolerability, pharmacokinetics, and pharmacodynamics in pediatric type 2 diabetes: a randomized, double-blind, placebo-controlled trial | Failure to meet inclusion critia (aim was to treat obesity combined with type 2 diabetes) |
| Paprocki 2022 | Clinical Presentation and Management of Diabetes Mellitus in Youth with Prader-Willi Syndrome | Failure to meet inclusion critia |
| Gemmill 2011 | Clinical trials in youth with type 2 diabetes | Failure to meet inclusion critia |
| Pino 2018 | Cohort profile: The MULTI sTUdy Diabetes rEsearch (MULTITUDE) consortium | Failure to meet inclusion critia |
| Katz 2015 | Alterations in left ventricular, left atrial, and right ventricular structure and function to cardiovascular risk factors in adolescents with type 2 diabetes participating in the TODAY clinical trial | Failure to meet inclusion critia |
| Handelsman 2015 | American Association of Clinical Endocrinologists and American College of Endocrinology - Clinical practice guidelines for developing a diabetes mellitus comprehensive care plan - 2015 | Failure to meet inclusion critia |
| Kapadia 2013 | Are the ADA hemoglobin A1c criteria relevant for the diagnosis of type 2 diabetes in youth? | Failure to meet inclusion critia |
| Franks 2008 | Assessing gene-treatment interactions at the FTO and INSIG2 loci on obesity-related traits in the Diabetes Prevention Program | Failure to meet inclusion critia |
| Aldossari 2023 | The Association between Type-1 Diabetes Mellitus and Risk of Depression among Saudi Patients: A Cross-Sectional Study | Failure to meet inclusion critia |
| Yeung 2022 | Baseline characteristics of participants in the Pre-Diabetes Interventions and Continued Tracking to Ease-out Diabetes (Pre-DICTED) Program | Failure to meet inclusion critia |
| Tell 2022 | Bromocriptine quick-release as adjunct therapy in youth and adults with type 1 diabetes: A randomized, placebo-controlled crossover study | Failure to meet inclusion critia |
| Sylvetsky 2015 | Buddy Study: Partners for better health in adolescents with type 2 diabetes | Failure to meet inclusion critia |
| Tamborlane 2014 | Can we get it right for youth with type 2 diabetes? | Failure to meet inclusion critia |
| Shukla 2017 | Carbohydrate-last meal pattern lowers postprandial glucose and insulin excursions in type 2 diabetes | Failure to meet inclusion critia |
| Barbour 2018 | A cautionary response to SMFM statement: pharmacological treatment of gestational diabetes | Failure to meet inclusion critia |
| Chhabra 2013 | Challenges in the management of type 2 diabetes mellitus and cardiovascular risk factors in obese subjects: What is the evidence and what are the myths? | Failure to meet inclusion critia |
| Tam 2013 | Changes in systemic inflammation in obese children with prediabetes after a 12 month lifestyle intervention; RESIST, a randomised control trial | Failure to meet inclusion critia |
| Dhaliwal 2019 | Changes in Visceral and Subcutaneous Fat in Youth With Type 2 Diabetes in the TODAY Study | Failure to meet inclusion critia |
| Mazur 2022 | Childhood Obesity: Position Statement of Polish Society of Pediatrics, Polish Society for Pediatric Obesity, Polish Society of Pediatric Endocrinology and Diabetes, the College of Family Physicians in Poland and Polish Association for Study on Obesity | Failure to meet inclusion critia |
| Matthews 2002 | Children with type 2 diabetes: The risks of complications | Failure to meet inclusion critia |
| Albarracin 2008 | Chromium picolinate and biotin combination improves glucose metabolism in treated, uncontrolled overweight to obese patients with type 2 diabetes | Failure to meet inclusion critia |
| Støa-Birketvedt 1998 | Cimetidine reduces weight and improves metabolic control in overweight patients with type 2 diabetes | Failure to meet inclusion critia |
| White 2009 | Clinical characteristics and co-morbidities in a large cohort of youth with type 2 diabetes mellitus (T2DM) screened for the treatment options for type 2 diabetes in adolescents and youth (TODAY) study | Failure to meet inclusion critia |
| Redondo 2019 | Comparison of serum adipokines and anthropometric measures in children with type 1 diabetes (T1D) and obesity | Failure to meet inclusion critia |
| Inge 2018 | Comparison of Surgical and Medical Therapy for Type 2 Diabetes in Severely Obese Adolescents | Failure to meet inclusion critia |
| Zirie 2003 | Complications of diabetes mellitus among patients attending outpatient clinics in Qatar | Failure to meet inclusion critia |
| Horowitz 2012 | Effect of the once-daily human GLP-1 analogue liraglutide on appetite, energy intake, energy expenditure and gastric emptying in type 2 diabetes | Failure to meet inclusion critia |
| Garg 2011 | Effects of colesevelam on LDL-C, A1c and GLP-1 levels in patients with type 1 diabetes: A pilot randomized double-blind trial | Failure to meet inclusion critia |
| NCT04095104 | Adjunct Phentermine + Topiramate After Bariatric Surgery in 12-24 Year Olds | Study protocol |
| NCT03801252 | Antibiotic Prophylaxis to Prevent Obesity-Related Induction Complications in Nulliparae at Term | Study protocol |
| NCT01665742 | Anti-inflammatory Dietary Intervention in Overweight and Obese Adolescents | Study protocol |
| Euctr 2014 | Continuous positive airway pressure with or without liraglutide in obstructive sleep apnoea and type 2 diabetes (ROMANCE) | Study protocol |
| Irct20111203008286N | The Effect of Cognitive Behavioral Program based on an Interactive Application on Serum Glucose Level and HbA1C of Family member of Patients with Type 2 diabetes | Study protocol |
| NCT00936663 | Using Sitagliptin as a Treatment to Prevent New Onset Diabetes After Kidney Transplantation | Study protocol |
| NCT01825798 | Treatment of Overweight Induced by Antipsychotic Medication in Young People With Autism Spectrum Disorders (ASD) | Study protocol |
| NCT01323283 | Study of Omega-3 Fatty Acids Regarding Ergogenic, Anti-inflammatory and Obesity Inhibitory Effects in 8 Year Old Children | Study protocol |
| Huang 2023 | The efficacy of metformin for the treatment of psoriasis: a meta-analysis study | Meta-analysis |
| Pascual-Morena 2021 | Physical Exercise vs. Metformin to Improve Delivery- and Newborn-Related Outcomes Among Pregnant Women With Overweight: A Network Meta-Analysis | Meta-analysis |
| Hsieh 2019 | Effects of different pharmacologic smoking cessation treatments on body weight changes and success rates in patients with nicotine dependence: A network meta-analysis | Meta-analysis |
| Ryan 2018 | Comparative effectiveness of canagliflozin, SGLT2 inhibitors and non-SGLT2 inhibitors on the risk of hospitalization for heart failure and amputation in patients with type 2 diabetes mellitus: A real-world meta-analysis of 4 observational databases (OBSERVE-4D) | Meta-analysis |
| Zhou 2017 | Effects of metformin on blood pressure in nondiabetic patients: a meta-analysis of randomized controlled trials | Meta-analysis |
| Kuzik 2016 | Evaluating the effects of metformin use on height in children and adolescents: A meta-analysis of randomized controlled trials | Meta-analysis |
| Monami 2013 | Further data on beta-blockers and cancer risk: Observational study and meta-analysis of randomized clinical trials | Meta-analysis |
| Wang 2011 | Glucagon-like peptide-1 receptor agonists versus insulin in inadequately controlled patients with type 2 diabetes mellitus: A meta-analysis of clinical trials | Meta-analysis |
| Czernichow 2010 | Efficacy of weight loss drugs on obesity and cardiovascular risk factors in obese adolescents: a meta-analysis of randomized controlled trials | Meta-analysis |
| Snethen 2006 | Effective weight loss for overweight children: A meta-analysis of intervention studies | Meta-analysis |
| Loomba 2009 | Advances in pediatric nonalcoholic fatty liver disease | Review |
| Suri 2021 | Advances in the Epidemiology, Diagnosis, and Management of Pediatric Fatty Liver Disease | Review |
| Harrison 2003 | Advances in the Understanding and Treatment of Nonalcoholic Fatty Liver Disease | Review |
| Handelsman 2015 | American Association of Clinical Endocrinologists and American College of Endocrinology - Clinical practice guidelines for developing a diabetes mellitus comprehensive care plan - 2015 | Review |
| Wang 2021 | Analysis of Time Course and Dose Effect From Metformin on Body Mass Index in Children and Adolescents | Review |
| Zhuang 2013 | Antidepressants for polycystic ovary syndrome | Review |
| Dayabandara 2017 | Antipsychotic-associated weight gain: Management strategies and impact on treatment adherence | Review |
| Goo 1996 | Metformin: A new treatment option for non-insulin-dependent diabetes mellitus | Review |
| Gourgari 2017 | Modifiable Risk Factors for Cardiovascular Disease in Children with Type 1 Diabetes: Can Early Intervention Prevent Future Cardiovascular Events? | Review |
| Katsiki 2011 | Naltrexone sustained-release (SR) + bupropion SR combination therapy for the treatment of obesity: 'A new kid on the block'? | Review |
| Alfie 2006 | Nonalcoholic fatty liver disease | Review |
| Kawasaki 2018 | Obesity and abnormal glucose tolerance in the offspring of mothers with diabetes | Review |
| Patel 2021 | Patient initiation and maintenance of GLP-1 RAs for treatment of obesity | Review |
| Liyanagedera 2017 | The pharmacological management of NAFLD in children and adolescents | Review |

**Appendix 5. Network plots**

**Supplemental Figure 1a.** Weight-related outcomes

**
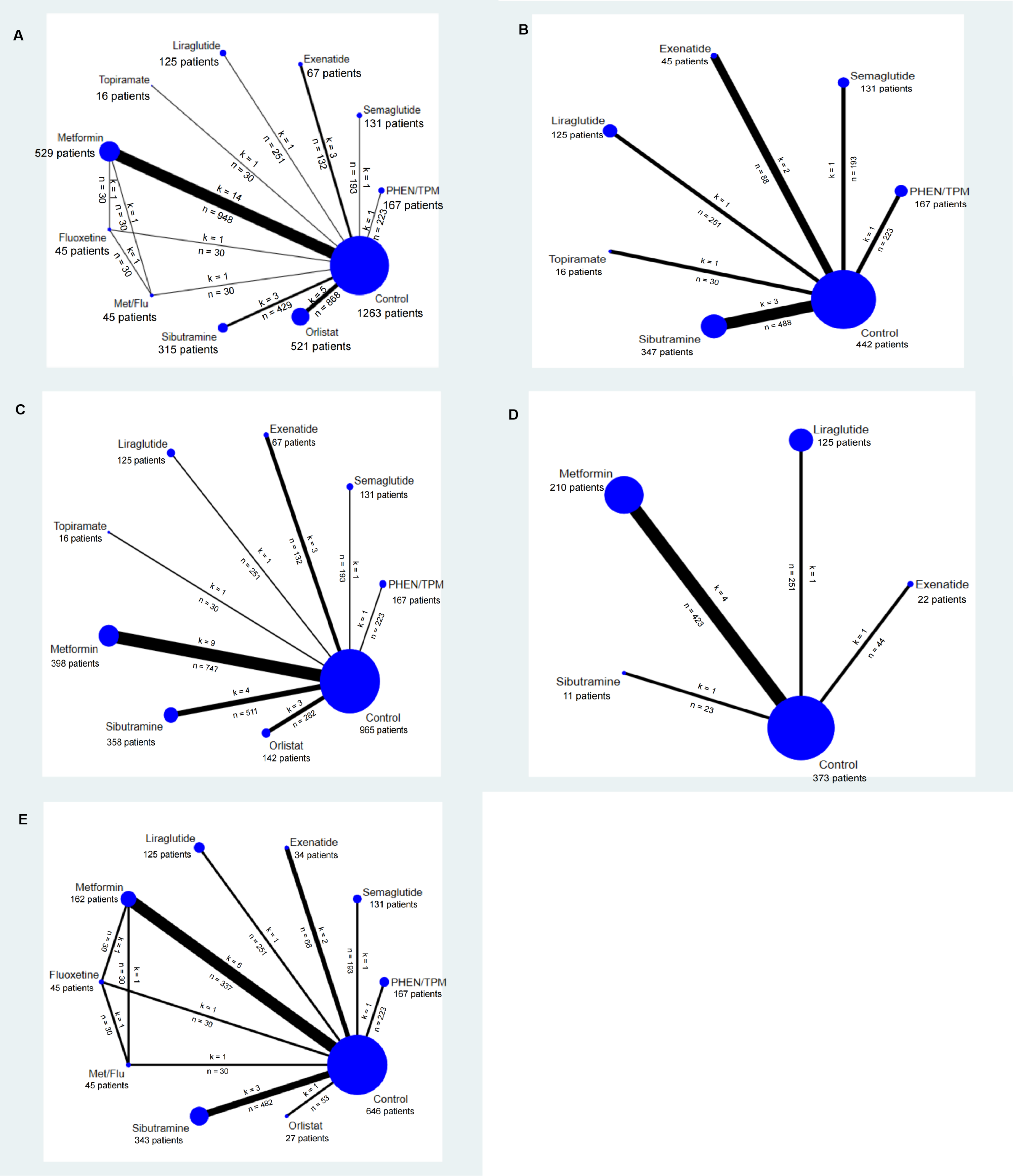
**

**Supplemental Figure 1b.** Metabolic outcomes

**
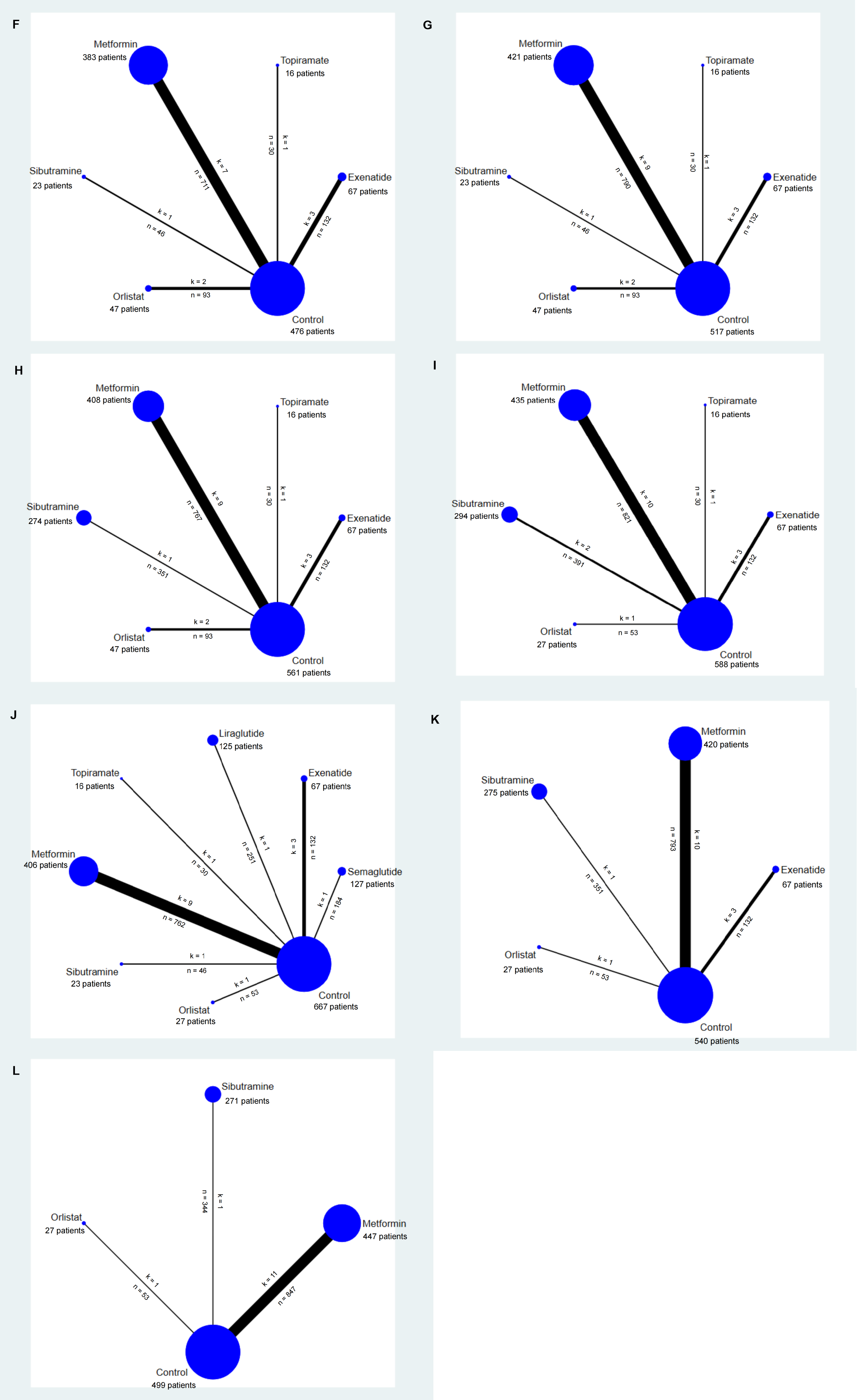
**

**Supplemental Figure 1c.** Anthropometric outcomes

**
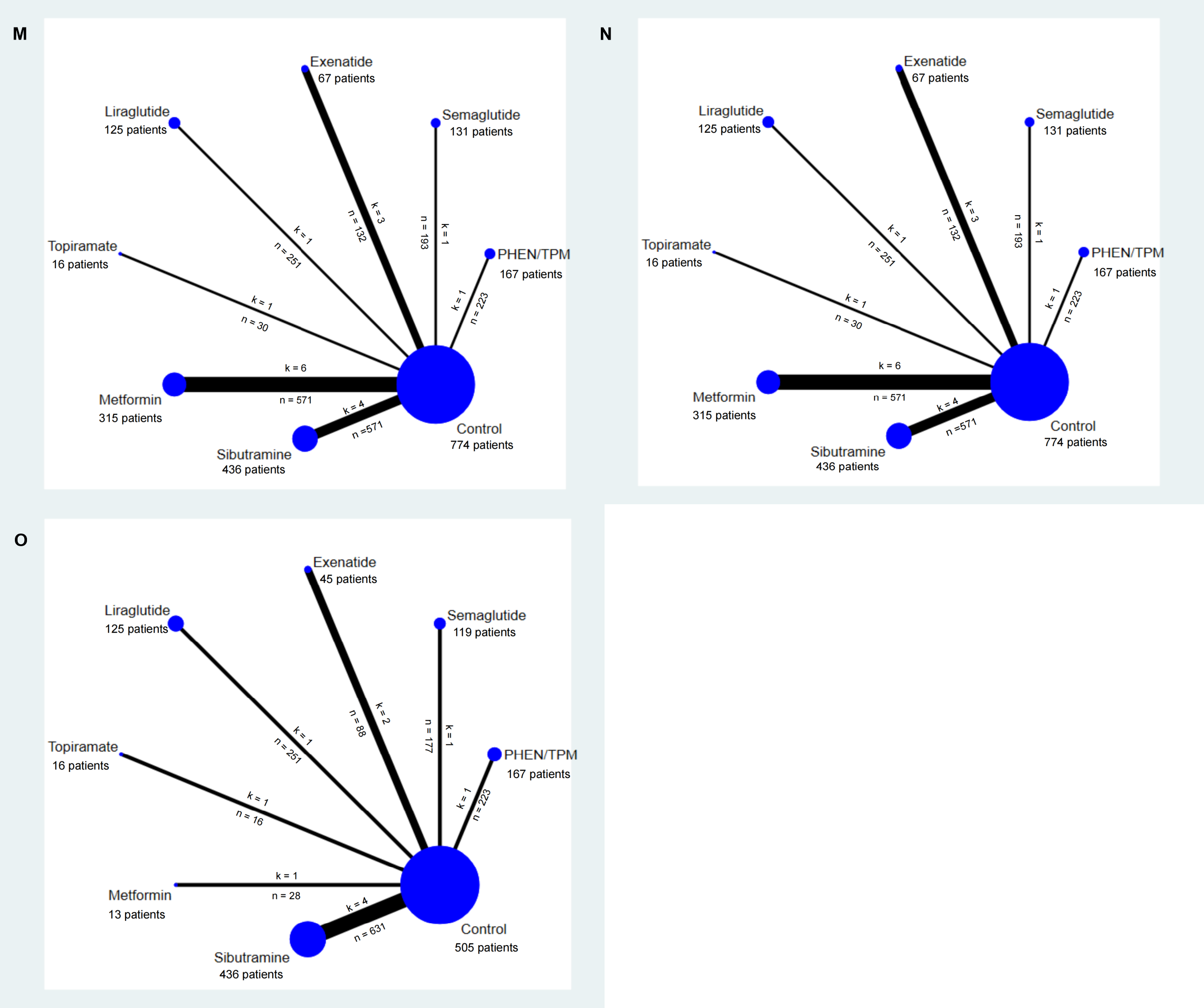
**

**Supplemental Figure 1d.** Safety outcomes

**
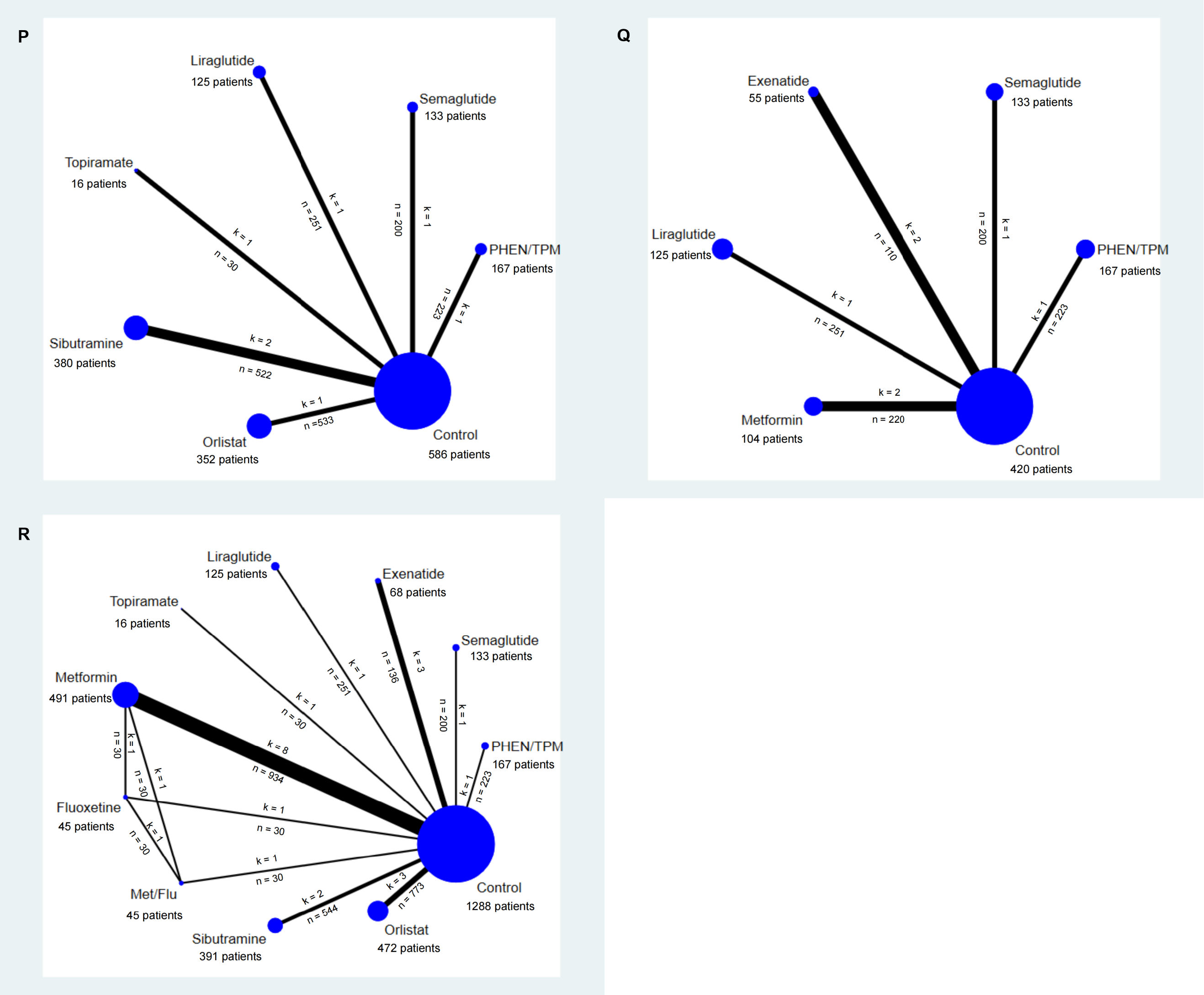
**

Graphical representation of networks for endpoints. Connecting lines

represent head-to-head comparisons (thickness proportional to number of trials) between drugs, indicated by nodes (size proportional to number of patients). Numbers above and below the lines indicate studies and patients, respectively.

**Abbreviation**: A, BMI; B, change in BMI; C, weight; D, BMI-SDS; E, waist circumference; F, TC; G, LDL-C; H, HDL-C; I, TG; J, FBG; K, FINS; L, HOMA-IR; M, SBP; N, DBP; O, heart rate; P, depression; Q, gastrointestinal disorders; R, serious adverse events; Control: placebo or no treatment; k: number of trials per comparison; n: number of patients per comparison.

**Appendix 6. Risk of bias assessment**

**Supplemental Table 5.** Risk of bias assessment for each outcome

| **Trial** | **Randomisation process** | **Deviations from the intended interventions** | **Missing outcome data** | **Measurement of the outcome** | **Selection of the reported result** | **Overall** |
| --- | --- | --- | --- | --- | --- | --- |
| Kelly 2022 | **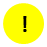** | 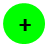 | 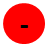 | 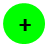 | 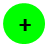 | 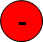 |
| Weghuber 2022 | 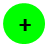 | 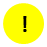 | 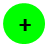 | 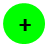 | 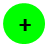 | 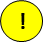 |
| Weghuber 2020 | 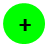 | 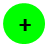 | 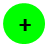 | 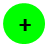 | 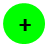 | 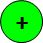 |
| Kelly 2013 | 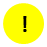 | 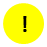 | 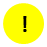 | 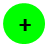 | 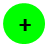 | 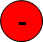 |
| Fox 2022 | 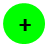 | 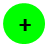 | 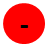 | 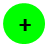 | 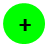 | 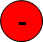 |
| Kelly 2020 | 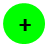 | 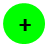 | 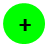 | 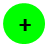 | 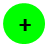 | 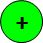 |
| Fox 2016 | 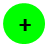 | 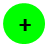 | 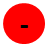 | 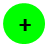 | 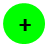 | 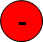 |
| Atabek 2008 | 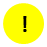 | 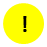 | 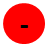 | 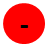 | 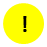 | 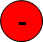 |
| Clarson 2009 | 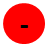 | 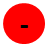 | 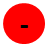 | 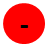 | 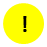 | 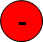 |
| Freemark 2007 | 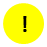 | 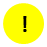 | 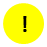 | 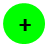 | 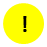 | 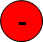 |
| Kendall 2013 | 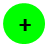 | 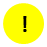 | 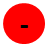 | 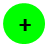 | 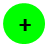 | 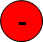 |
| Mauras 2012 | 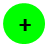 | 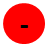 | 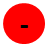 | 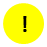 | 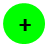 | 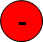 |
| Rezvanian 2010 | 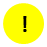 | 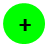 | 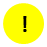 | 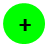 | 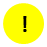 | 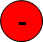 |
| Wiegand 2010 | 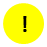 | 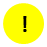 | 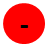 | 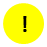 | 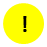 | 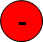 |
| Wilson 2010 | 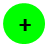 | 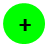 | 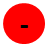 | 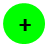 | 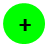 | 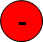 |
| Yanovski 2011 | 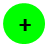 | 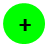 | 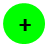 | 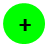 | 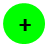 | 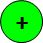 |
| Pastor-Villaescusa 2016 |  |  |  |  |  |  |
| Warnakulasuriya 2018 |  |  |  |  |  |  |
| Burgert 2008 |  |  |  |  |  |  |
| Garibay-Nieto 2017 |  |  |  |  |  |  |
| Evia-Viscarra 2012 |  |  |  |  |  |  |
| Berkowitz 2003 |  |  |  |  |  |  |
| Berkowitz 2006 |  |  |  |  |  |  |
| García-Morales 2006 |  |  |  |  |  |  |
| Van Mil 2007 |  |  |  |  |  |  |
| Chanoine 2005 |  |  |  |  |  |  |
| Maahs 2006 (a) |  |  |  |  |  |  |
| NCT00001723 |  |  |  |  |  |  |
| Ozkan 2004 |  |  |  |  |  |  |
| Zahmatkesh 2023 |  |  |  |  |  |  |

| Low risk | Some concerns | High risk |
| --- | --- | --- |

**Supplemental figure 2**. Risk of bias assessment for overall included studies

**Supplemental Table 6. Risk of bias for included studies**

| **Trial** | **Bias** | **Authors' judgement** | **Support for judgement** |
| --- | --- | --- | --- |
| Kelly 2022 | Randomisation process | Some concerns | Insuffificient information in the study about the process of allocation concealment. Baseline characteristics were similar in the two groups. |
|  | Deviations from the intended interventions | Low risk | The author confirmed that all participants and trial personnel were blinded. The efficacy endpoint was analyzed based on the intention-to-treat (ITT) principle. |
|  | Missing outcome data | High risk | High attrition rate likely to affect objective outcomes (The study was completed by 29 (56.9%), 37 (75.5%), and 73 (65.2%) participants receiving placebo, mid-dose PHEN/TPM, and top-dose PHEN/TPM, respectively). |
|  | Measurement of the outcome | Low risk | The methods employed for measuring outcomes were appropriate and consistent throughout the intervention groups. Blinding of outcome assessment was not reported; however, the outcomes were unlikely to be influenced by the knowledge of treatment allocation. |
|  | Selection of the reported result | Low risk | Identical outcomes were reported in both the clinical trial register and the publication (NCT03922945). |
| Weghuber 2022 | Randomisation process | Low risk | An appropriate randomization approach was employed (randomization was performed with the use of an interactive Web-response system). Baseline characteristics were similar in the two groups. |
|  | Deviations from the intended interventions | Low risk | The author affirmed that all participants and trial personnel were kept blinded. The efficacy endpoint was analyzed in accordance with the intention-to-treat (ITT) principle. |
|  | Missing outcome data | Low risk | Dropouts: 14 (10.4%) in the semaglutide group and 7 (10.4%) in the placebo group. The characteristics of the subjects who withdrew were provided. |
|  | Measurement of the outcome | Low risk | The methods utilized for measuring outcomes were appropriate and consistent across all intervention groups. Blinding of outcome assessment was not reported; nevertheless, the outcomes were highly unlikely to be affected by the knowledge of treatment allocation. |
|  | Selection of the reported result | Low risk | Identical outcomes were documented both in the clinical trial register and the publication (NCT04102189). |
| Weghuber 2020 | Randomisation process | Low risk | An appropriate randomization method was utilized (visit based on a computer-generated randomization scheme and trial drugs were similar in appearance). Baseline characteristics were similar in the two groups. |
|  | Deviations from the intended interventions | Low risk | The author verified that both participants and personnel were blinded. The primary analysis was conducted on the intention-to-treat (ITT) population. |
|  | Missing outcome data | Low risk | Dropouts: 3 (13.6%) in the exenatide group and 4 (18.1%) in the placebo group. The characteristics of the subjects who withdrew were presented. |
|  | Measurement of the outcome | Low risk | The methods utilized for measuring outcomes were appropriate and consistent across all intervention groups. Blinding of outcome assessment was not reported; nevertheless, the outcomes were highly unlikely to be affected by the knowledge of treatment allocation. |
|  | Selection of the reported result | Low risk | Identical outcomes were recorded both in the clinical trial registry and the publication (NCT02794402). |
| Kelly 2013 | Randomisation process | Some concerns | Details regarding randomisation were not furnished. Insufficient information was presented. Baseline characteristics were similar in the two groups. |
|  | Deviations from the intended interventions | Some concerns | Inadequate information (the method of blinding was unclear) |
|  | Missing outcome data | Some concerns | Dropouts: 1 (7.7%) occurred in the exenatide group and 3 (23.0%) in the placebo group. There was a significantly higher dropout rate in the placebo group. Analysis was conducted on intention-to-treat (ITT). |
|  | Measurement of the outcome | Low risk | The methods employed for measuring outcomes were appropriate and consistent throughout the intervention groups. Blinding of outcome assessment was not reported; however, the outcomes were unlikely to be influenced by the knowledge of treatment allocation. |
|  | Selection of the reported result | Low risk | Identical outcomes were reported in both the clinical trial register and the publication (NCT01237197). |
| Fox 2022 | Randomisation process | Low risk | An appropriate randomization approach was employed (randomization, using permuted blocks of 2, 4, or 6, was computer-generated and codes were maintained by the University of Minnesota Investigational Drug Service Pharmacy). Baseline characteristics were similar in the two groups. |
|  | Deviations from the intended interventions | Low risk | Treatment allocation was blinded to the participants, investigators, coordinators, data collectors, and sponsors throughout the trial. The primary analysis compared mean percent change between exenatide XR and placebo groups using the intention-to-treat (ITT) population, adjusting for randomization BMI for enhanced precision. |
|  | Missing outcome data | High risk | Dropouts: 3 (9.0%) emerged in the exenatide XR group and 7 (21.2%) in the placebo group. There was a significantly higher dropout rate in the placebo group. The disparity was significant. |
|  | Measurement of the outcome | Low risk | The methods employed for measuring outcomes were appropriate and consistent throughout the intervention groups. |
|  | Selection of the reported result | Low risk | Identical outcomes were reported in both the clinical trial register and the publication (NCT02496611). |
| Kelly 2020 | Randomisation process | Low risk | Randomization was stratified according to pubertal status (Tanner stage 2 or 3 vs. Tanner stage 4 or 5) and glycemic status (normoglycemia vs. prediabetes or type 2 diabetes) and was performed with the use of an interactive Web response system, in permuted blocks, with block sizes of 4. Baseline characteristics were similar in the two groups. |
|  | Deviations from the intended interventions | Low risk | The author affirmed that all participants and trial personnel were kept blinded. Primary and secondary efficacy end points were analyzed according to the intention-to-treat principle. |
|  | Missing outcome data | Low risk | Dropouts: 24 (19.2%) occurred in the liraglutide group and 26 (20.6%) in the placebo group. Baseline characteristics between the subjects completed and the drop outs were similar. All missing data were handled with the multipleimputation method under the assumption that participants who were withdrawn from the trial responded as though they had been treated with placebo for the entire trial. To investigate the sensitivity of the results of the main analysis of the primary end point with regard to the handling of missing data, an ANCOVA was performed with imputation of missing values according to the last observation carried forward. |
|  | Measurement of the outcome | Low risk | The methods employed for measuring outcomes were appropriate and consistent throughout the intervention groups. Blinding of outcome assessment was not reported; however, the outcomes were unlikely to be influenced by the knowledge of treatment allocation. |
|  | Selection of the reported result | Low risk | Identical outcomes were reported in both the clinical trial register and the publication (NCT02918279). |
| Fox 2016 | Randomisation process | Low risk | An appropriate randomization method was utilized (randomization scheme was generated based on randomly permuted blocks of size 2, 4, and 6 and maintained by the University of Minnesota—Fairview Investigational Drug Service Pharmacy). Baseline characteristics were similar in the two groups. |
|  | Deviations from the intended interventions | Low risk | The author verified that both participants and personnel were blinded. The primary analysis was conducted on the intention-to-treat (ITT) population. |
|  | Missing outcome data | High risk | Dropouts: 4 (25.0%) emerged in the topiramate group and 5 (35.7%) in the placebo group. There was a significantly higher dropout rate in the placebo group. The disparity was significant. |
|  | Measurement of the outcome | Low risk | The methods employed for measuring outcomes were appropriate and consistent throughout the intervention groups. Blinding of outcome assessment was not reported; however, the outcomes were unlikely to be influenced by the knowledge of treatment allocation. |
|  | Selection of the reported result | Low risk | Identical outcomes were reported in both the clinical trial register and the publication (NCT01859013). |
| Atabek 2008 | Randomisation process | Some concerns | No description of randomisation process, and no description of how allocation was concealed. |
|  | Deviations from the intended interventions | Some concerns | Unsure who was blinded. |
|  | Missing outcome data | High risk | The trial did not report the number of dropouts, or clarify there  were no dropouts. |
|  | Measurement of the outcome | High risk | It remains ambiguous whether the outcome evaluators were cognizant of the interventions received by the study participants; It is uncertain whether the assessment of outcomes is impacted by the acquired knowledge of the intervention; It is indistinct whether the assessment of outcomes might have been influenced by the received knowledge of the intervention. |
|  | Selection of the reported result | Some concerns | No results were presented for food consumption data. Additionally, it was highly ambiguous regarding the number lost to follow-up and the types of analyses that were conducted. |
| Clarson 2009 | Randomisation process | High risk | The author confirmed that the allocation was not concealed. No placebo was administered to the control group. However, it remains unclear whether there were baseline differences. |
|  | Deviations from the intended interventions | High risk | The absence of a placebo in the control group implied that blinding of participants and personnel could not be accomplished. The author confirmed that neither participants nor personnel were blinded. The assessment of outcomes was not blinded, as affirmed by the author. |
|  | Missing outcome data | High risk | The rate of trial dropouts was relatively low; however, no imputation method was employed. |
|  | Measurement of the outcome | High risk | The outcomes assessment was not blinded as affirmed by the author. It remains ambiguous whether the outcome evaluators were aware of the interventions received by the study participants. It is uncertain whether the assessment of outcomes is affected by the acquired knowledge of the intervention. It is indistinct whether the assessment of outcomes might have been influenced by the received knowledge of the intervention. |
|  | Selection of the reported result | Some concerns | There was no previously published protocol; thus, reporting bias could not be accessed. |
| Freemark 2007 | Randomisation process | Some concerns | An appropriate randomization approach was employed (following enrolment, patients were randomized to the metformin and placebo groups by a research pharmacist using computer-generated randomization tables). However, the baseline BMI was discrepant between the two groups. |
|  | Deviations from the intended interventions | Some concerns | Both the subject or research staff was blinded to the intervention. It remains ambiguous whether appropriate analyses were employed to evaluate the effect of the distributive intervention. It is indistinct whether the failure to analyze the randomized participants is likely to have a considerable impact on the results. |
|  | Missing outcome data | Some concerns | The trial did not report the number of dropouts, or clarify there  were no dropouts. |
|  | Measurement of the outcome | Low risk | The methods employed for measuring outcomes were appropriate and consistent throughout the intervention groups. Blinding of outcome assessment was not reported; however, the outcomes were unlikely to be influenced by the knowledge of treatment allocation. |
|  | Selection of the reported result | Some concerns | Since no protocol was published before trial was completed, it is  unclear whether all outcomes were reported. |
| Kendall 2013 | Randomisation process | Low risk | An appropriate randomization method was utilized (Independent pharmacists dispensed either metformin or placebo according to a computer-generated randomization list for each stratification group (males 8–13 yr, females 8–13 yr, males 14–18 yr, and females 14–18 yr).  The thirdparty, concealed allocation process ensured that participantsand all investigators were unaware of the allocated treatment). |
|  | Deviations from the intended interventions | Some concerns | Although it is unclear who was blinded (this was a prospective, randomized, double-blind, placebo-controlled trial), consider interventions that do not deviate from expectations in accordance with the experimental setting. |
|  | Missing outcome data | High risk | Dropouts: 19 (25.7%) emerged in the metformin group and 22 (28.6%) in the placebo group. The dropout rate was high and no imputation approach was employed to replace the missing data. |
|  | Measurement of the outcome | Low risk | The methods employed for measuring outcomes were appropriate and consistent throughout the intervention groups. Blinding of outcome assessment was not reported; however, the outcomes were unlikely to be influenced by the knowledge of treatment allocation. |
|  | Selection of the reported result | Low risk | Identical outcomes were reported in both the clinical trial register and the publication (ISRCTN19517475). |
| Mauras 2012 | Randomisation process | Low risk | Adequate randomisation process (randomisation assignments were balanced for pubertal status. We used sealed envelopes with equal amount of labels organized at random for pubertal and pre-pubertal kids to choose from at their CRC visit (baseline)). The author of the trial verified that allocation was concealed through the sealed envelopes. |
|  | Deviations from the intended interventions | High risk | No placebo was administered to the control group; consequently, the participants could not have been blinded. |
|  | Missing outcome data | High risk | Dropouts: 12 (34.3%) emerged in the metformin group and 12 (38.7%) in the placebo group. The dropout rate was high and no imputation approach was employed to replace the missing data. |
|  | Measurement of the outcome | Some concerns | The outcomes assessment was not blinded. It remains ambiguous whether the outcome evaluators were aware of the interventions received by the study participants. It is uncertain whether the assessment of outcomes is affected by the acquired knowledge of the intervention. It is indistinct whether the assessment of outcomes might have been influenced by the received knowledge of the intervention. |
|  | Selection of the reported result | Low risk | Identical outcomes were reported in both the clinical trial register and the publication (NCT00139477). |
| Rezvanian 2010 | Randomisation process | Some concerns | Adequate randomization process (Participants were randomly assigned into four groups (n = 45 for each group) by a computer-generated random numbers table using the children's record numbers in our clinic, but it was unclear whether the allocation was concealed). |
|  | Deviations from the intended interventions | Low risk | participants and personnel would have been blinded (triple-masked randomized clinical trial). |
|  | Missing outcome data | Some concerns | An imputation approach was not employed to substitute for the missing data; nevertheless, the dropout rate was relatively low. |
|  | Measurement of the outcome | Low risk | The methods employed for measuring outcomes were appropriate and consistent throughout the intervention groups. |
|  | Selection of the reported result | Some concerns | It was impossible to evaluate whether all outcomes were reported due to the unavailability of a protocol. |
| Wiegand 2010 | Randomisation process | Some concerns | There is no description of the randomization process and it is unclear whether the allocation was concealed. The groups did not differ significantly by gender (female 72 vs 62%; PZ0.35), age (15.1 vs 15.0 years; PZ0.98), ethnicity (Caucasian 87.3 vs 91.2%; PZ0.11), pubertal stage (Tanner O III 87.1 vs 90.5%; PZ0.71), or BMI (34.3 vs 35.5 kg/m2; PZ0.35). |
|  | Deviations from the intended interventions | Some concerns | Although it is unclear who was blinded (we performed a double-blind, randomized controlled clinical trial), consider interventions that do not deviate from expectations in accordance with the experimental setting. |
|  | Missing outcome data | High risk | Dropouts: 2 (5.6%) emerged in the metformin group and 5 (14.7%) in the placebo group. No imputation approach was employed to substitute the missing data. |
|  | Measurement of the outcome | Some concerns | The methods employed for measuring outcomes were appropriate and consistent throughout the intervention groups. Blinding of outcome assessment was not reported. It is uncertain whether the assessment of outcomes is affected by the acquired knowledge of the intervention. It is indistinct whether the assessment of outcomes might have been influenced by the received knowledge of the intervention. |
|  | Selection of the reported result | Some concerns | The clinical trial entry could not be located; thus, it remains uncertain whether selective reporting took place. |
| Wilson 2010 | Randomisation process | Low risk | An appropriate randomization method was employed, along with adequate allocation concealment (Subjects who successfully completed the run-in period were randomized to metformin XR or placebo treatment according to random sequences constructed at the Data Coordinating Center; Subjects and study personnel were blinded to assignment throughout  the entire study). |
|  | Deviations from the intended interventions | Low risk | Performance bias is likely to be mitigated through blinding participants and trial personnel (Subjects and study personnel were blinded to assignment throughout the entire study; Unblinded data were seen only by the Data and Safety Monitoring Board and study statistician). |
|  | Missing outcome data | High risk | The dropout rate was relatively high in each group and no imputation method was implemented to replace the missing data (ninety-two subjects were screened and 77 were randomized, 39 to metformin XR, 38 to placebo; 27 and 19 in each group were measured at weeks 52 and 100, respectively). |
|  | Measurement of the outcome | Low risk | The methods employed for measuring outcomes were appropriate and consistent throughout the intervention groups. |
|  | Selection of the reported result | Low risk | Identical outcomes were reported in both the clinical trial register and the publication (NCT00209482, NCT00120146). |
| Yanovski 2011 | Randomisation process | Low risk | An appropriate randomization method was employed, along with adequate allocation concealment (We randomly assigned participants in a 1:1 randomization ratio to receive metformin hydrochloride or placebo, twice daily with meals. Investigators assigned consecutive code numbers to participants from prespecified lists stratified by race/ethnicity, sex, and degree of pubertal development; The CRC Pharmaceutical Development Section used permuted blocks with stratification to generate allocations that translated code numbers into study group assignments by using a pseudo-random number program and prepared identically appearing placebo and metformin capsules). |
|  | Deviations from the intended interventions | Low risk | Both the participants and personnel were blinded (no participant, investigator, or other medical or nursing staff interacting with participants was aware of study group assignments during the trial).  An appropriate analysis was employed to assess the effect of assignment to intervention (we assessed efficacy in the intention-to-treat sample of all randomly assigned participants using a multiple imputation model for missing data under a missing-at-random assumption). |
|  | Missing outcome data | Low risk | There is a low risk of attrition bias regarding subjective outcomes (we assessed efficacy in the intention-to-treat sample of all randomly assigned participants using a multiple imputation model for missing data under a missing-at-random assumption). |
|  | Measurement of the outcome | Low risk | The methods employed for measuring outcomes were appropriate and consistent throughout the intervention groups. |
|  | Selection of the reported result | Low risk | Identical outcomes were reported in both the clinical trial register and the publication (NCT00005669). |
| Pastor-Villaescusa 2016 | Randomisation process | Low risk | An appropriate randomization method was utilized (accordance with a randomization schedule generated by the Pharmacy Service of the Virgen de las Nieves University Hospital in Granada. MAS 100 version 2.1 software (GlaxoWelcome, Madrid, Spain) was used by the Support Consortium to Biomedical Research Network). Baseline characteristics were similar in the two groups. |
|  | Deviations from the intended interventions | Some concerns | All research staff was blinded to both the treatment allocation during the time of the study and the data analysis. Less appropriate analyses were employed to evaluate the effects of the allocation intervention (The data associated with the subjects who dropped out were subsequently excluded from the statistical analysis). |
|  | Missing outcome data | High risk | Dropouts: 12 (15.0%) emerged in the metformin group and 8 (10.0%) in the placebo group. No imputation approach was employed to substitute the missing data. |
|  | Measurement of the outcome | Low risk | The methods employed for measuring outcomes were appropriate and consistent throughout the intervention groups. |
|  | Selection of the reported result | Low risk | Identical outcomes were reported in both the clinical trial register and the publication (EudraCT, ID: 2010-023061-21). |
| Warnakulasuriya 2018 | Randomisation process | Some concerns | An appropriate randomization method was utilized (following the baseline assessment, participants in each age stratum were separately randomized into two parallel treatment arms to receive one of the two management protocols on a 1:1 basis using random numbers generated by the principal investigator using Microsoft Excel 2010. manufactured metformin and placebo and both tablets look similar except for the active pharmacological compound in one). Nevertheless, the baseline levels of fasting insulin (FINS) differed between the two groups. |
|  | Deviations from the intended interventions | High risk | No appropriate analysis was employed to assess the effect of the distributive intervention; Analysis could not be conducted on randomly grouped participants, which might have a substantial influence on the results. |
|  | Missing outcome data | High risk | Dropouts: 98 (59%) emerged in the metformin group and 91 (52.6%) in the placebo group. Dropout fairly high in each group and no imputation method was performed to replace missing data. |
|  | Measurement of the outcome | Low risk | The methods employed for measuring outcomes were appropriate and consistent throughout the intervention groups. |
|  | Selection of the reported result | Low risk | Identical outcomes were reported in both the clinical trial register and the publication (NCT02274948). |
| Burgert 2008 | Randomisation process | Some concerns | Details regarding randomisation were not furnished. Insufficient information was presented. Baseline characteristics were similar in the two groups. |
|  | Deviations from the intended interventions | Some concerns | Inadequate information (the method of blinding was unclear). |
|  | Missing outcome data | High risk | Dropouts: 2 (11.8%) emerged in the metformin group and 4 (23.5%) in the placebo group. A much higher dropout rate at the placebo group. No imputation method was performed to replace missing data. |
|  | Measurement of the outcome | Some concerns | The methods employed for measuring outcomes were appropriate and consistent throughout the intervention groups. Blinding of outcome assessment was not reported, and it was likely that the outcomes were influenced by the knowledge of treatment allocation. |
|  | Selection of the reported result | Some concerns | The clinical trial entry could not be located; thus, it remains uncertain whether selective reporting took place. |
| Garibay-Nieto 2017 | Randomisation process | Some concerns | Details regarding randomisation were not furnished. Insufficient information was presented. |
|  | Deviations from the intended interventions | Some concerns | Unsure who was blinded. |
|  | Missing outcome data | High risk | Dropouts: 10 (41.7%) emerged in the metformin group and 12 (40.0%) in the placebo group. The dropout rate was relatively high in each group and no imputation method was implemented to replace the missing data. |
|  | Measurement of the outcome | Low risk | The methods employed for measuring outcomes were appropriate and consistent throughout the intervention groups. |
|  | Selection of the reported result | Low risk | Identical outcomes were reported in both the clinical trial register and the publication (NCT02063802). |
| Evia-Viscarra 2012 | Randomisation process | Low risk | An appropriate randomization method was utilized (patients were divided into two groups (placebo group and metformin group) using a table of random numbers). Baseline characteristics were similar in the two groups. |
|  | Deviations from the intended interventions | High risk | Unsure who was blinded. No appropriate analysis was employed to assess the effect of the distributive intervention; Analysis could not be conducted on randomly grouped participants, which might have a substantial influence on the results. |
|  | Missing outcome data | High risk | Dropouts: 3 (20.0%) emerged in the metformin group and 2 (12.5%) in the placebo group. No imputation method was performed to replace missing data. |
|  | Measurement of the outcome | Low risk | The methods employed for measuring outcomes were appropriate and consistent throughout the intervention groups. |
|  | Selection of the reported result | Low risk | Identical outcomes were reported in both the clinical trial register and the publication (NCT01410604). |
| Berkowitz 2003 | Randomisation process | Some concerns | There was no description of the randomisation process and no account of how the allocation was concealed. |
|  | Deviations from the intended interventions | Low risk | The risk of performance bias is likely to be low due to the blinding of participants, parents, and trial personnel (participants, parents, and all study personnel were blinded to treatment condition during phase 1. Only the research pharmacist was aware of treatment status). |
|  | Missing outcome data | Low risk | Although the number of dropouts was small, a more appropriate imputation approach could have been employed to enhance data analysis. The imputation method was only utilized for primary outcome measures (weight and waist circumference, which were objectively measured). |
|  | Measurement of the outcome | Low risk | The methods employed for measuring outcomes were appropriate and consistent throughout the intervention groups. |
|  | Selection of the reported result | Low risk | Identical outcomes were reported in both the clinical trial register and the publication (NCT00212173). |
| Berkowitz 2006 | Randomisation process | Low risk | An appropriate randomization method was employed (the randomization schedule was stratified by center and baseline BMI (≤37 kg/m2 or >37 kg/m2) and was computer-generated in blocks of 4 by the sponsor. Each site was responsible for assigning sequential treatments within each stratum), along with adequate allocation concealment (the sponsor kept allocation codes sealed and secure until the database was locked before analysis). Baseline characteristics were similar in the two groups. |
|  | Deviations from the intended interventions | Low risk | The risk of performance bias is likely to be low due to the blinding of participants, parents, and trial personnel (participants, their parents, and study personnel were blinded to treatment). |
|  | Missing outcome data | High risk | Dropouts: 87 (23.6%) emerged in the sibutramine group and 50 (38.5%) in the placebo group. LOCF was solely employed to substitute for the missing BMI data; other objective outcome data were presented for completers only. The dropout rate was moderately high and was relatively higher in the placebo group compared to the drug group. It was difficult to assess the level of attrition bias based on these factors. |
|  | Measurement of the outcome | Low risk | The methods employed for measuring outcomes were appropriate and consistent throughout the intervention groups. |
|  | Selection of the reported result | Low risk | Identical outcomes were reported in both the clinical trial register and the publication (NCT00261911). |
| García-Morales 2006 | Randomisation process | Some concerns | An appropriate randomization method was employed (patients were block-randomized by using a computer generated list), along with adequate allocation concealment (patients were block-randomized by using a computer generated list. All the materials for a patient were identified by the patient number. The placebo and drug capsules were identical in appearance and smell. The trial medications were prepared by one author (A.B.), who did not know the identity of the patients. Another author (L.M.G.-M.) received the trial materials without any knowledge of the procedures or order in the random number list). Nevertheless, the baseline heart rate differed between the two groups. |
|  | Deviations from the intended interventions | Some concerns | Unclear who was blinded (this was a 6 month, randomized, double blind, placebo-controlled, prospective clinical trial of sibutramine QD). |
|  | Missing outcome data | Some concerns | Dropouts: 5 (19.2%) emerged in the sibutramine group and 6 (24.0%) in the placebo group. LOCF and modified intention-to-treat analysis were employed to replace the missing data for the primary outcomes. Nevertheless, the 5 participants who dropped out prior to the first month were not included. |
|  | Measurement of the outcome | Low risk | The methods employed for measuring outcomes were appropriate and consistent throughout the intervention groups. |
|  | Selection of the reported result | Some concerns | The clinical trial entry could not be located; thus, it remains uncertain whether selective reporting took place. |
| Van Mil 2007 | Randomisation process | Low risk | The author clarified the randomisation process; however, it remained unclear whether the process would have introduced selection bias (randomisation was performed by Knoll Pharmaceuticals. Boxes with  medication for each visit were numbered for each subject. Subjects received their number and the boxes with medication that belonged to that number. The numbers/medication was handed out in order of inclusion in the study). Allocation was concealed (Knoll Pharmaceuticals BV [currently Abbott Laboratories (Hoofddorp, The Netherlands)], manufactured and provided code-numbered placebo and sibutramine capsules. Subjects received their trial and medication code according to order of entrance into the study, without stratification). Baseline characteristics were similar in the two groups. |
|  | Deviations from the intended interventions | Low risk | Author confirmed participants and personnel were blinded |
|  | Missing outcome data | Low risk | An imputation method was employed; however, results were only presented for completers. The dropout rates were relatively low. |
|  | Measurement of the outcome | Low risk | The methods employed for measuring outcomes were appropriate and consistent throughout the intervention groups. |
|  | Selection of the reported result | Some concerns | The clinical trial entry could not be located; thus, it remains uncertain whether selective reporting took place. |
| Chanoine 2005 | Randomisation process | Low risk | An appropriate randomization method was employed (patients were randomized centrally according to a computer-generated randomization schedule prepared by the study’s sponsor, with stratification by body weight (<80 kg or ≥80 kg) on day 1 and by weight loss during the  lead-in period (<1 kg or ≥1 kg)), along with adequate allocation concealment (the allocation process was triple-blind; the allotted treatment group was obtained through an automated telephone system). Baseline characteristics were similar in the two groups. |
|  | Deviations from the intended interventions | Some concerns | The author confirmed all participants, trial personnel and outcome  assessors were blinded. Even though an imputation method (LOCF) was employed, the missing data were not incorporated into the analysis. The effect on objective outcomes remains unclear. |
|  | Missing outcome data | High risk | Even though an imputation method was used (LOCF), dropout  rates were high. Effect on objective outcomes unclear. |
|  | Measurement of the outcome | Low risk | The methods employed for measuring outcomes were appropriate and consistent throughout the intervention groups. |
|  | Selection of the reported result | Some concerns | The clinical trial entry could not be located; thus, it remains uncertain whether selective reporting took place. |
| Maahs 2006 | Randomisation process | Low risk | An appropriate randomization method was employed , along with adequate allocation concealment (the GCRC statistician generated the randomization sequence before the start of the study. The list of randomization assignments was sealed and sent to the study pharmacist, who had no contact with the study subjects). Baseline characteristics were similar in the two groups. |
|  | Deviations from the intended interventions | Low risk | Participants and personnel were blinded (only the research pharmacist was aware of treatment status). An appropriate analysis was employed to assess the effect of assignment to intervention (results were calculated for the 34 subjects who completed the study, but they were also assessed by using intention-to-treat analysis (N = 40). |
|  | Missing outcome data | Some concerns | An imputation method was not employed to substitute for the missing data; however, the dropout rate was relatively low. |
|  | Measurement of the outcome | Low risk | The methods employed for measuring outcomes were appropriate and consistent throughout the intervention groups. |
|  | Selection of the reported result | Some concerns | The clinical trial entry could not be located; thus, it remains uncertain whether selective reporting took place. |
| NCT00001723 | Randomisation process | Low risk | An appropriate randomization method was employed (we randomized participants in a 1:1 fashion to orlistat 120 mg or identical appearing placebo thrice daily with meals plus a daily multivitamin (Centrum, Whitehall-Robins Healthcare, Madison, NJ) containing 5000 IU vitamin A (80% as retinol, 20% as beta carotene), 400 IU vitamin D as ergocalciferol, 30 IU vitamin E (as di-α tocopheryl acetate), and 25 mcg vitamin K (as phytonadione). Investigators assigned consecutive code  numbers to participants from pre-specified lists that were stratified by race  (Caucasian versus African American), sex (Male, Female), and degree of pubertal development (3 strata for boys: testes <15ml, testes 15-20mL, and testes >20mL; for girls: Breast Tanner stage I-III; Tanner stage IV, and Tanner stage V). The NIH CRC Pharmaceutical Development Section used permuted blocks with stratification to generate allocations that translated code numbers into trial group assignments by using a pseudo-random number program), along with adequate allocation concealment (pharmacy personnel not involved with the conduct of the study, dispensed identical-appearing study capsules in containers that differed only by participant code number. During the trial, no participant, investigator, or other medical or nursing staff interacting with participants was aware of study group assignments). Baseline characteristics were similar in the two groups. |
|  | Deviations from the intended interventions | Low risk | Participants and personnel were blinded (Subject, Caregiver, Investigator, Outcomes Assessor). |
|  | Missing outcome data | Some concerns | According to ClinicalTrials.gov, 87% of orlistat participants completed the trial, 84% completed placebo arm. An imputation method was not employed to substitute for the missing data. |
|  | Measurement of the outcome | Low risk | The methods employed for measuring outcomes were appropriate and consistent throughout the intervention groups. |
|  | Selection of the reported result | Some concerns | Insufficient information to permit judgement. |
| Ozkan 2004 | Randomisation process | High risk | An inappropriate randomisation method was used (randomisation was done by alternation of successive patients, who met the inclusion criteria, to receive conventional treatment alone or orlistat in addition to conventional treatment). Allocation was likely not concealed due to the randomisation method used. |
|  | Deviations from the intended interventions | High risk | The control group did not receive a placebo and thus could not have been blinded (the true benefit of orlistat versus conventional therapy remains to be determined in a larger placebo-controlled study). |
|  | Missing outcome data | High risk | An imputation method for replacing missing data was not carried out, and the dropout rate was moderate. |
|  | Measurement of the outcome | High risk | It is unclear whether the outcome evaluators were blinded and the assessment of outcomes was influenced by knowledge of the interventions received. |
|  | Selection of the reported result | Some concerns | The clinical trial entry could not be located; thus, it remains uncertain whether selective reporting took place. |
| Zahmatkesh 2023 | Randomisation process | Low risk | An appropriate randomization method was employed , along with adequate allocation concealment (BMI Z score and sex were randomly assigned by stratified randomization and the permuted block randomization technique with quadruple and binary blocks to ensure the uniform distribution of these parameters in the groups. Owing to the study’s doubleblind nature, sets of packages containing orlistat were made by someone other than the researcher prior to the study’s start, and the placebo had a similar appearance to orlistat. Indeed, from the time that participants were divided into the intervention and control groups until the conclusion of the intervention, researchers in the evaluation phase (anthropometric measures and laboratory tests) were unaware of the groups into which the patients were randomized.). Baseline characteristics were similar in the two groups. |
|  | Deviations from the intended interventions | Some concerns | It remains ambiguous whether appropriate analyses were employed to evaluate the effect of the distributive intervention. It is indistinct whether the failure to analyze the randomized participants is likely to have a considerable impact on the results. |
|  | Missing outcome data | Some concerns | Dropouts: 3 (10.0%) emerged in the orlistat group and 4 (13.3%) in the placebo group. An imputation method was not employed to substitute for the missing data. |
|  | Measurement of the outcome | Low risk | The methods employed for measuring outcomes were appropriate and consistent throughout the intervention groups. |
|  | Selection of the reported result | Low risk | Identical outcomes were reported in both the clinical trial register and the publication (IRCT20220409). |

**Appendix 7. Comparison-adjusted funnel plot for the network meta-analysis**

**Supplemental Figure 3a.** Weight-related outcomes

**Supplemental Figure 3b.** Metabolic outcomes

**Supplemental Figure 3c.** Anthropometric outcomes

**Supplemental Figure 3d.** Safety outcomes

In this presentation, all studies are centered on the summary effect estimate of their respective comparisons [μXY (logOR for present study)], which is represented by the vertical red line. Individual study-level effect sizes are represented by yiXY [where X and Y are two study agents]. μxy is the comparison-specific summary estimate for x versus y. The outer dotted lines represent a 95% CI for the difference between study-specific effect sizes and comparison-specific summary estimates. Please note that this is drawn only for comparisons with two or more studies.

**Abbreviation**: A, BMI; B, change in BMI; C, weight; D, BMI-SDS; E, waist circumference; F, TC; G, LDL-C; H, HDL-C; I, TG; J, FBG; K, FINS; L, HOMA-IR; M, SBP; N, DBP; O, heart rate; P, depression; Q, gastrointestinal disorders; R, serious adverse events; Control: placebo or no treatment.

**Appendix 8. Results of a pairwise meta-analysis for each outcome**

Supplemental Table 7. Results of a pairwise meta-analysis for each outcome

| **Item** | **Comparison** | **No. of studies** | **Participants** | **WMD**  **(95% CI)** | **Heterogeneity (*I*^2^ %)** | **P-value** | **Publication bias**  **(Egger test, P-value)** |
| --- | --- | --- | --- | --- | --- | --- | --- |
| **BMI** | PHEN/TPM vs. Control | 1 | 167/56 | **-6.97 (-9.84, -4.10)** | NA | NA | NA |
|  | Semaglutide vs. Control | 1 | 131/62 | **-5.90 (-7.04, -4.76)** | NA | NA | NA |
|  | Exenatide vs. Control | 3 | 67/65 | **-1.26 (-2.00, -0.52)** | 0.0 | 0.707 | NA |
|  | Liraglutide vs. Control | 1 | 125/126 | **-1.58 (-2.47, -0.69)** | NA | NA | NA |
|  | Topiramate vs. Control | 1 | 16/14 | -0.86 (-2.18, 0.46) | NA | NA | NA |
|  | Sibutramine vs. Control | 3 | 315/114 | **-1.37 (-2.69, -0.06)** | 66.4 | 0.051 | NA |
|  | Fluoxetine vs. Control | 1 | 15/15 | **-0.80 (-0.86, -0.75)** | NA | NA | NA |
|  | Met/Flu vs. Control | 1 | 15/15 | **-1.10 (-1.12, -1.08)** | NA | NA | NA |
|  | Metformin vs. Control | 14 | 499/449 | **-1.06 (-1.88, -0.24)** | **91.1** | **0.000** | 0.265 |
|  | Orlistat vs. Control | 5 | 521/347 | **-1.29 (-1.93, -0.64)** | **78.3** | **0.001** | NA |
|  | Metformin vs. Fluoxetine | 1 | 15/15 | **1.50 (1.43, 1.57)** | NA | NA | NA |
|  | Metformin vs. Met/Flu | 1 | 15/15 | **1.80 (1.75, 1.85)** | NA | NA | NA |
|  | Fluoxetine vs. Met/Flu | 1 | 15/15 | **0.30 (0.25, 0.35)** | NA | NA | NA |
| **Percent change in BMI** | PHEN/TPM vs. Control | 1 | 167/56 | **-9.70 (-12.93, -6.47)** | NA | NA | NA |
|  | Semaglutide vs. Control | 1 | 131/62 | **-16.10 (-19.18, -13.02)** | NA | NA | NA |
|  | Exenatide vs. Control | 2 | 45/43 | **-3.31 (-5.47, -1.14)** | 6.3 | 0.302 | NA |
|  | Liraglutide vs. Control | 1 | 125/126 | **-4.64 (-7.12, -2.16)** | NA | NA | NA |
|  | Topiramate vs. Control | 1 | 16/14 | -1.89 (-5.22, 1.44) | NA | NA | NA |
|  | Sibutramine vs. Control | 3 | 347/141 | **-5.78 (-8.62, -2.93)** | **70.9** | **0.032** | NA |
| **BMI-SDS** | Exenatide vs. Control | 1 | 22/22 | -0.10 (-0.37, 0.17) | NA | NA | NA |
|  | Liraglutide vs. Control | 1 | 125/126 | **-0.23 (-0.37, -0.09)** | NA | NA | NA |
|  | Metformin vs. Control | 4 | 210/213 | **-0.10 (-0.16, -0.04)** | 0.0 | 0.570 | NA |
|  | Sibutramine vs. Control | 1 | 11/12 | -0.13 (-0.27, 0.01) | NA | NA | NA |
| **Weight** | PHEN/TPM vs. Control | 1 | 167/56 | **-14.59 (-17.47, -11.72)** | NA | NA | NA |
|  | Semaglutide vs. Control | 1 | 131/62 | **-18.00 (-21.48, -14.52)** | NA | NA | NA |
|  | Exenatide vs. Control | 3 | 67/65 | **-3.78 (-5.96, -1.61)** | 0.0 | 0.575 | NA |
|  | Liraglutide vs. Control | 1 | 125/126 | **-4.51 (-7.17, -1.85)** | NA | NA | NA |
|  | Topiramate vs. Control | 1 | 16/14 | -2.43 (-6.24, 1.38) | NA | NA | NA |
|  | Metformin vs. Control | 9 | 398/349 | **-2.67 (-3.59, -1.76)** | 0.0 | 0.784 | NA |
|  | Sibutramine vs. Control | 4 | 358/153 | **-4.33 (-8.18, -0.49)** | **90.1** | **0.000** | NA |
|  | Orlistat vs. Control | 3 | 142/140 | -5.50 (-11.02, 0.02) | **86.7** | **0.001** | NA |
| **Waist circumference** | PHEN/TPM vs. Control | 1 | 167/56 | **-8.98 (-12.07, -5.89)** | NA | NA | NA |
|  | Semaglutide vs. Control | 1 | 131/62 | **-12.20 (-14.84, -9.56)** | NA | NA | NA |
|  | Exenatide vs. Control | 2 | 34/32 | -1.43 (-4.76, 1.91) | 0.0 | 0.653 | NA |
|  | Liraglutide vs. Control | 1 | 125/126 | **-2.93 (-5.32, -0.54)** | NA | NA | NA |
|  | Sibutramine vs. Control | 3 | 343/139 | **-5.85 (-7.34, -4.36)** | 0.0 | 0.615 | NA |
|  | Fluoxetine vs. Control | 1 | 15/15 | **-0.20 (-0.22, -0.18)** | NA | NA | NA |
|  | Met/Flu vs. Control | 1 | 15/15 | **-0.90 (-1.00, -0.80)** | NA | NA | NA |
|  | Metformin vs. Control | 5 | 162/175 | -0.97 (-2.26, 0.33) | 56.4 | 0.057 | NA |
|  | Orlistat vs. Control | 1 | 27/26 | **-3.27 (-4.71, -1.83)** | NA | NA | NA |
|  | Metformin vs. Fluoxetine | 1 | 15/15 | 0.00 (-0.02, 0.02) | NA | NA | NA |
|  | Metformin vs. Met/Flu | 1 | 15/15 | **0.70 (0.60, 0.80)** | NA | NA | NA |
|  | Fluoxetine vs. Met/Flu | 1 | 15/15 | **0.70 (0.60, 0.80)** | NA | NA | NA |
| **FBG** | Semaglutide vs. Control | 1 | 127/57 | **-0.20 (-0.36, -0.04)** | NA | NA | NA |
|  | Exenatide vs. Control | 3 | 67/65 | -0.05 (-0.23, 0.12) | 0.0 | 0.741 | NA |
|  | Liraglutide vs. Control | 1 | 125/126 | -0.10 (-0.24, 0.04) | NA | NA | NA |
|  | Topiramate vs. Control | 1 | 16/14 | -0.43 (-0.93, 0.07) | NA | NA | NA |
|  | Sibutramine vs. Control | 1 | 23/23 | 0.03 (-0.31, 0.37) | NA | NA | NA |
|  | Metformin vs. Control | 9 | 406/356 | -0.05 (-0.13, 0.02) | 0.0 | 0.518 | NA |
|  | Orlistat vs. Control | 1 | 27/26 | **-0.24 (-0.38, -0.10)** | NA | NA | NA |
| **FINS** | Exenatide vs. Control | 3 | 67/65 | -0.35 (-4.49, 3.80) | 21.3 | 0.281 | NA |
|  | Sibutramine vs. Control | 1 | 275/76 | **-7.10 (-10.65, -3.55)** | NA | NA | NA |
|  | Metformin vs. Control | 10 | 420/373 | **-3.90 (-7.32, -0.48)** | **75.9** | **0.000** | 0.784 |
|  | Orlistat vs. Control | 1 | 27/26 | **-2.50 (-3.75, -1.25)** | NA | NA | NA |
| **HOMA-IR** | Sibutramine vs. Control | 1 | 271/73 | **-12.20 (-18.17, -6.23)** | NA | NA | NA |
|  | Metformin vs. Control | 11 | 447/400 | -0.70 (-1.54, 0.14) | **68.3** | **0.000** | 0.943 |
|  | Orlistat vs. Control | 1 | 27/26 | **-0.80 (-1.16, -0.44)** | NA | NA | NA |
| **TC** | Exenatide vs. Control | 3 | 67/65 | 3.65 (-3.95, 11.25) | 0.0 | 0.374 | NA |
|  | Topiramate vs. Control | 1 | 16/14 | 2.85 (-9.85, 15.55) | NA | NA | NA |
|  | Metformin vs. Control | 7 | 383/328 | -2.11 (-6.03, 1.81) | 0.0 | 0.94 | NA |
|  | Sibutramine vs. Control | 1 | 23/23 | 17.60 (-2.02, 37.22) | NA | NA | NA |
|  | Orlistat vs. Control | 2 | 47/46 | **-8.77 (-13.95, -3.59)** | 0.0 | 0.377 | NA |
| **LDL-C** | Exenatide vs. Control | 3 | 67/65 | 4.70 (-2.17, 11.57) | 0.0 | 0.420 | NA |
|  | Topiramate vs. Control | 1 | 16/14 | 7.76 (-2.38, 17.90) | NA | NA | NA |
|  | Metformin vs. Control | 9 | 421/369 | 1.00 (-2.74, 4.74) | 23.5 | 0.234 | NA |
|  | Sibutramine vs. Control | 1 | 23/23 | 4.30 (-13.66, 22.26) | NA | NA | NA |
|  | Orlistat vs. Control | 2 | 47/46 | -3.98 (-11.76, 3.79) | 23.1 | 0.254 | NA |
| **HDL-C** | Exenatide vs. Control | 3 | 67/65 | -0.18 (-6.72, 6.36) | 53.7 | 0.115 | NA |
|  | Topiramate vs. Control | 1 | 16/14 | 2.67 (-1.81, 7.15) | NA | NA | NA |
|  | Metformin vs. Control | 9 | 408/359 | -0.31 (-1.80, 1.19) | 0.0 | 0.470 | NA |
|  | Sibutramine vs. Control | 1 | 274/77 | 3.00 (1.25, 4.75) | NA | NA | NA |
|  | Orlistat vs. Control | 2 | 47/46 | 1.53 (0.38, 2.69) | 0.0 | 0.347 | NA |
| **TG** | Exenatide vs. Control | 3 | 67/65 | -6.02 (-22.11, 10.07) | 0.0 | 0.463 | NA |
|  | Topiramate vs. Control | 1 | 16/14 | **-42.52 (-81.17, -3.87)** | NA | NA | NA |
|  | Metformin vs. Control | 10 | 435/386 | -2.78 (-6.09, 0.53) | **80.1** | **0.000** | 0.814 |
|  | Sibutramine vs. Control | 2 | 294/97 | **-24.20 (-36.71, -11.70)** | 0.0 | 0.600 | NA |
|  | Orlistat vs. Control | 1 | 27/26 | **-4.40 (-7.38, -1.42)** | NA | NA | NA |
| **SBP** | PHEN/TPM vs. Control | 1 | 167/56 | -2.44 (-6.03, 1.15) | NA | NA | NA |
|  | Semaglutide vs. Control | 1 | 131/62 | -2.00 (-5.04, 1.04) | NA | NA | NA |
|  | Exenatide vs. Control | 3 | 67/65 | -3.42 (-7.18, 0.34) | 0.0 | 0.661 | NA |
|  | Liraglutide vs. Control | 1 | 125/126 | -2.05 (-4.54, 0.44) | NA | NA | NA |
|  | Topiramate vs. Control | 1 | 16/14 | -0.77 (-7.72, 6.18) | NA | NA | NA |
|  | Sibutramine vs. Control | 4 | 436/195 | **1.12 (0.25, 2.00)** | 0.0 | 0.719 | NA |
|  | Metformin vs. Control | 6 | 315/256 | -0.33 (-0.81, 0.16) | 0.0 | 0.459 | NA |
| **DBP** | PHEN/TPM vs. Control | 1 | 167/56 | **-3.30 (-6.24, -0.36)** | NA | NA | NA |
|  | Semaglutide vs. Control | 1 | 131/62 | -1.00 (-3.52, 1.52) | NA | NA | NA |
|  | Exenatide vs. Control | 3 | 67/65 | -1.89 (-5.40, 1.62) | 0.0 | 0.985 | NA |
|  | Liraglutide vs. Control | 1 | 125/126 | 1.23 (-0.68, 3.14) | NA | NA | NA |
|  | Topiramate vs. Control | 1 | 16/14 | 0.94 (-4.99, 6.87) | NA | NA | NA |
|  | Sibutramine vs. Control | 4 | 436/195 | 1.70 (-0.93, 4.34) | **72.7** | **0.012** | NA |
|  | Metformin vs. Control | 6 | 315/256 | 0.03 (-0.63, 0.69) | 33.0 | 0.188 | NA |
| **Heart rate** | PHEN/TPM vs. Control | 1 | 167/56 | 1.68 (-2.15, 5.51) | NA | NA | NA |
|  | Semaglutide vs. Control | 1 | 119/58 | 1.00 (-3.08, 5.08) | NA | NA | NA |
|  | Exenatide vs. Control | 2 | 45/43 | 4.64 (-1.56, 10.84) | 30.4 | 0.231 | NA |
|  | Liraglutide vs. Control | 1 | 125/126 | 2.01 (-0.57, 4.59) | NA | NA | NA |
|  | Topiramate vs. Control | 1 | 16/14 | -1.46 (-8.67, 5.75) | NA | NA | NA |
|  | Sibutramine vs. Control | 4 | 436/195 | **4.89 (1.63, 8.15)** | 54.7 | 0.085 | NA |
|  | Metformin vs. Control | 1 | 15/13 | **-8.00 (-15.89, -0.11)** | NA | NA | NA |
| **Depression** | PHEN/TPM vs. Control | 1 | 167/56 | 4.55 (0.25, 82.03) | NA | NA | NA |
|  | Semaglutide vs. Control | 1 | 133/67 | 1.53 (0.06, 38.02) | NA | NA | NA |
|  | Liraglutide vs. Control | 1 | 125/126 | 1.71 (0.40, 7.31) | NA | NA | NA |
|  | Topiramate vs. Control | 1 | 16/14 | 0.87 (0.05, 15.28) | NA | NA | NA |
|  | Sibutramine vs. Control | 2 | 380/142 | 2.13 (0.35, 12.96) | 0.0 | 0.763 | NA |
|  | Orlistat vs. Control | 1 | 352/181 | 2.59 (0.12, 54.22) | NA | NA | NA |
| **Gastrointestinal disorders** | PHEN/TPM vs. Control | 1 | 167/56 | 0.77 (0.32, 1.87) | NA | NA | NA |
|  | Semaglutide vs. Control | 1 | 133/67 | 1.53 (0.61, 38.02) | NA | NA | NA |
|  | Exenatide vs. Control | 2 | 55/55 | 2.50 (0.48, 12.96) | 57.0 | 0.127 | NA |
|  | Liraglutide vs. Control | 1 | 125/126 | 3.20 (1.91, 5.36) | NA | NA | NA |
|  | Metformin vs. Control | 2 | 104/116 | 0.93 (0.28, 3.17) | 68.2 | 0.076 | NA |
| **SAE** | PHEN/TPM vs. Control | 1 | 167/56 | 1.71 (0.08, 36.09) | NA | NA | NA |
|  | Semaglutide vs. Control | 1 | 133/67 | 1.29 (0.48, 3.50) | NA | NA | NA |
|  | Exenatide vs. Control | 3 | 68/68 | 3.09 (0.12, 78.70) | NA | NA | NA |
|  | Liraglutide vs. Control | 1 | 125/126 | 0.60 (0.14, 2.55) | NA | NA | NA |
|  | Topiramate vs. Control | 1 | 16/14 | NA | NA | NA | NA |
|  | Sibutramine vs. Control | 2 | 391/153 | 3.60 (0.46, 28.43) | NA | NA | NA |
|  | Fluoxetine vs. Control | 1 | 15/15 | NA | NA | NA | NA |
|  | Met/Flu vs. Control | 1 | 15/15 | NA | NA | NA | NA |
|  | Metformin vs. Control | 8 | 461/473 | 2.55 (0.35, 18.52) | 0.0 | 0.900 | NA |
|  | Metformin vs. Fluoxetine | 1 | 15/15 | NA | NA | NA | NA |
|  | Metformin vs. Met/Flu | 1 | 15/15 | NA | NA | NA | NA |
|  | Fluoxetine vs. Met/Flu | 1 | 15/15 | NA | NA | NA | NA |
|  | Orlistat vs. Control | 3 | 472/301 | 1.04 (0.40, 2.74) | 0.0 | 0.442 | NA |
|  | SGLT-2 inhibitor vs. Control | 4 | 241/3967 | **1.28**  **(1.06, 1.56)** | 0.0 | 0.92 |  |

**Appendix 9. Results of network meta-analysis**

**Supplemental Table 8.** Results of network meta-analysis for FBG and FINS

| **Exenatide** | NA | NA | -2.76‡  (-10.72, 5.30) | NA | -1.46‡  (-13.41, 11.26) | -6.06‡  (-18.46, 6.99) | 1.06‡  (-5.67, 8.46) |
| --- | --- | --- | --- | --- | --- | --- | --- |
| 0.05‡  (-0.17, 0.27) | **Liraglutide** | NA | NA | NA | NA | NA | NA |
| 0.15‡  (-0.08, 0.38) | 0.10‡  (-0.11, 0.31) | **Semaglutide** | NA | NA | NA | NA | NA |
| 0.00‡  (-0.19, 0.19) | -0.05‡  (-0.20, 0.11) | -0.15‡  (-0.32, 0.03) | **Metformin** | NA | 1.29‡  (-9.18, 12.46) | -3.33‡  (-14.28, 8.35) | **3.81**†  **(0.24, 7.98)** |
| 0.38‡  (-0.15, 0.90) | 0.33‡  (-0.18, 0.84) | 0.23‡  (-0.29, 0.75) | 0.38‡  (-0.13, 0.88) | **Topiramate** | NA | NA | NA |
| 0.19‡  (-0.04, 0.41) | 0.14‡  (-0.06, 0.34) | 0.04‡  (-0.17, 0.25) | **0.19***  **(0.03, 0.35)** | -0.19‡  (-0.71, 0.33) | **Orlistat** | -4.62‡  (-19.32, 10.05) | 2.49‡  (-7.64, 12.64) |
| -0.08‡  (-0.46, 0.31) | -0.13‡  (-0.50, 0.24) | -0.23‡  (-0.61, 0.15) | -0.08‡  (-0.43, 0.27) | -0.46‡  (-1.06, 0.15) | -0.27‡  (-0.64, 0.10) | **Sibutramine** | 7.12‡  (-3.51, 17.78) |
| -0.05‡  (-0.22, 0.12) | -0.10†  (-0.24, 0.04) | **-0.20***  **(-0.36, -0.04)** | -0.05‡  (-0.13, 0.02) | -0.43‡  (-0.93, 0.07) | **-0.24***  **(-0.38, -0.10)** | 0.03‡  (-0.31, 0.37) | **Control** |

Treatment FBG (MD with 95% Crl) FINS (MD with 95% Crl)

Results of network meta-analysis for FBG and FINS were listed in the left lower half (S1) and upper right half (S2).

S1. FBG (*N*_T_ = 14 [47%], *n*_t_ = 1458 [38%], Bayesian fixed-effects model, *I*^2^ = 0.0%)

S2. FINS (*N*_T_ = 17 [57%], *n*_t_ = 1329 [35%], *I*^2^ = 3.0%)

Comparisons should be read from left to right, and the estimate is in the cell in common between the column-defining treatment and

the row-defining treatment. A mean difference (MD) of less than 0 indicates that the outcome is more likely with treatment (column) than reference (row). Significant results are in bold and underscored. The evidence is graded using the CINeMA system (Confidence in Network Meta-Analysis), an adaptation of the GRADE (Grading of Recommendations, Assessment, Development, and Evaluations) approach for network meta-analysis

*Control* placebo or no treatment, *NA* not available

*Moderate quality of evidence. †Low quality of evidence. ‡Very low quality of evidence. *N_T_* total number of trials reporting the outcome (percentage of sample), *n_t_* total number of patients available for the respective outcome (percentage of sample)

**Supplemental Table 9.** Results of network meta-analysis for HOMA-IR and HDL-C

| **Exenatide** | -1.18‡  (-5.90, 4.03) | 1.92‡  (-5.30, 9.71) | 0.41‡  (-5.17, 6.17) | 2.24‡  (-3.67, 9.05) | -0.73‡  (-4.91, 4.06) |
| --- | --- | --- | --- | --- | --- |
| NA | **Metformin** | 3.07‡  (-3.22, 9.56) | 1.62‡  (-2.87, 5.52) | 3.42†  (-1.44, 8.58) | 0.43‡  (-1.61, 2.66) |
| NA | NA | **Topiramate** | -1.49‡  (-8.81, 5.28) | 0.35‡  (-7.17, 7.85) | -2.65‡  (-8.67, 3.44) |
| NA | 0.10‡  (-3.15, 3.29) | NA | **Orlistat** | 1.78‡  (-3.59, 8.07) | -1.24‡  (-4.41, 2.88) |
| NA | **11.51**‡  **(4.80, 18.20)** | NA | **11.43**‡  **(4.15, 18.65)** | **Sibutramine** | -3.00†  (-7.56, 1.55) |
| NA | -0.70‡  (-1.77, 0.34) | NA | -0.80‡  (-3.83, 2.25) | **-12.22**‡  **(-18.82, -5.59)** | **Control** |

Treatment HOMA-IR (MD with 95% Crl) HDL-C (MD with 95% Crl)

Results of network meta-analysis for HOMA-IR and HDL-C were listed in the left lower half (S1) and upper right half (S2).

S1. HOMA-IR (*N*_T_ = 13 [43%], *n*_t_ = 1244 [33%], *I*^2^ = 8.0%)

S2. HDL-C (*N*_T_ = 16 [53%], *n*_t_ = 1373 [36%], *I*^2^ = 1.0%)

Comparisons should be read from left to right, and the estimate is in the cell in common between the column-defining treatment and

the row-defining treatment. A mean difference (MD) of less than 0 indicates that the outcome is more likely with treatment (column) than reference (row). Significant results are in bold and underscored. The evidence is graded using the CINeMA system (Confidence in Network Meta-Analysis), an adaptation of the GRADE (Grading of Recommendations, Assessment, Development, and Evaluations) approach for network meta-analysis

*Control* placebo or no treatment, *NA* not available

†Low quality of evidence. ‡Very low quality of evidence. *N_T_* total number of trials reporting the outcome (percentage of sample), *n_t_* total number of patients available for the respective outcome (percentage of sample)

**Supplemental Table 10.** Results of network meta-analysis for LDL-C and TC

| **Exenatide** | -5.79*  (-14.28, 2.74) | -0.82‡  (-15.60, 13.99) | -12.44*  (-21.64, 3.26) | 13.89‡  (-7.14, 35.11) | -3.67‡  (-11.22, 3.92) |
| --- | --- | --- | --- | --- | --- |
| 3.44‡  (-6.03, 12.51) | **Metformin** | 4.95‡  (-8.34, 18.27) | **-6.64**‡  **(-13.17, -0.16)** | 19.69‡  (-0.30, 39.72) | 2.12‡  (-1.80, 6.03) |
| -3.29‡  (-18.52, 11.48) | -6.68‡  (-20.01, 6.41) | **Topiramate** | -11.62‡  (-25.40, 2.15) | 14.76‡  (-8.72, 38.21) | -2.84‡  (-15.59, 9.86) |
| 8.90‡  (-4.01, 19.31) | 5.53‡  (-4.74, 13.41) | 12.11‡  (-3.39, 26.26) | **Orlistat** | **26.35**‡  **(6.03, 46.73)** | **8.77***  **(3.57, 13.96)** |
| -0.04‡  (-21.03, 20.84) | -3.46‡  (-23.17, 16.45) | 3.25‡  (-20.07, 26.13) | -8.88‡  (-29.49, 12.55) | **Sibutramine** | -17.58‡  (-37.23, 2.11) |
| 4.43‡  (-4.13, 12.50) | 0.99‡  (-3.15, 5.11) | 7.68‡  (-4.76, 20.24) | -4.52‡  (-11.40, 4.71) | 4.50‡  (-14.99, 23.64) | **Control** |

Treatment LDL-C (MD with 95% Crl) TC (MD with 95% Crl)

Results of network meta-analysis for LDL-C and TC were listed in the left lower half (S1) and upper right half (S2).

S1. LDL-C (*N*_T_ = 16 [53%], *n*_t_ = 1091 [29%], *I*^2^ = 6.0%)

S2. TC (*N*_T_ = 14 [47%], *n*_t_ = 1012 [26%], Bayesian fixed-effects model, *I*^2^ = 0.0%)

Comparisons should be read from left to right, and the estimate is in the cell in common between the column-defining treatment and

the row-defining treatment. A mean difference (MD) of less than 0 indicates that the outcome is more likely with treatment (column) than reference (row). Significant results are in bold and underscored. The evidence is graded using the CINeMA system (Confidence in Network Meta-Analysis), an adaptation of the GRADE (Grading of Recommendations, Assessment, Development, and Evaluations) approach for network meta-analysis

*Control* placebo or no treatment, *NA* not available

*Moderate quality of evidence. ‡Very low quality of evidence. *N_T_* total number of trials reporting the outcome (percentage of sample), *n_t_* total number of patients available for the respective outcome (percentage of sample)

**Supplemental Table 11.** Network meta-analysis results for TG

| **Exenatide** |  |  |  |  |  |
| --- | --- | --- | --- | --- | --- |
| 4.57‡  (-27.75, 40.17) | **Metformin** |  |  |  |  |
| 39.28‡  (-24.97, 104.67) | 34.45‡  (-25.51, 93.33) | **Topiramate** |  |  |  |
| 1.06*  (-51.32, 54.70) | -3.09‡  (-50.74, 41.47) | -37.95‡  (-110.48, 33.45) | **Orlistat** |  |  |
| 18.50‡  (-29.18, 64.88) | 14.05‡  (-26.99, 50.90) | -20.70‡  (-89.27, 46.43) | 17.57*  (-40.00, 72.26) | **Sibutramine** |  |
| -3.21‡  (-32.93, 27.78) | -7.76‡  (-23.94, 6.28) | -42.41‡  (-99.84, 14.99) | -4.40‡  (-47.84, 39.34) | -21.81‡  (-56.58, 15.26) | **Control** |

Treatment TG (MD with 95% Crl)

TG (*N*_T_ = 17 [57%], *n*_t_ = 1427 [37%], *I*^2^ = 10.0%)

Comparisons should be read from left to right, and the estimate is in the cell in common between the column-defining treatment and

the row-defining treatment. A mean difference (MD) of less than 0 indicates that the outcome is more likely with treatment (column) than reference (row). Significant results are in bold and underscored. The evidence is graded using the CINeMA system (Confidence in Network Meta-Analysis), an adaptation of the GRADE (Grading of Recommendations, Assessment, Development, and Evaluations) approach for network meta-analysis

*Control* placebo or no treatment, *NA* not available

*Moderate quality of evidence. †Low quality of evidence. ‡Very low quality of evidence. *N_T_* total number of trials reporting the outcome (percentage of sample), *n_t_* total number of patients available for the respective outcome (percentage of sample)

**Supplemental Table 12.** Results of network meta-analysis for SBP and DBP

| **Exenatide** | 3.05‡  (-2.87, 8.89) | 0.83‡  (-5.15, 6.88) | 2.04‡  (-2.49, 6.72) | 2.73‡  (-5.39, 10.75) | -1.50‡  (-7.85, 4.73) | 3.44‡  (-1.27, 8.24) | 1.82‡  (-2.40, 5.94) |
| --- | --- | --- | --- | --- | --- | --- | --- |
| -1.36‡  (-5.89, 3.14) | **Liraglutide** | -2.22‡  (-8.37, 3.88) | -1.06‡  (-5.46, 3.86) | -0.28‡  (-8.43, 7.75) | -4.58*  (-10.85, 1.74) | 0.39‡  (-4.30, 5.39) | -1.23‡  (-5.49, 2.98) |
| -1.42‡  (-6.23, 3.41) | -0.05‡  (-3.97, 3.88) | **Semaglutide** | 1.18‡  (-3.44, 6.26) | 1.91‡  (-6.34, 10.10) | -2.33‡  (-8.80, 4.16) | 2.59‡  (-2.26, 7.82) | 0.98‡  (-3.45, 5.43) |
| -3.09‡  (-6.88, 0.70) | -1.72‡  (-4.24, 0.81) | -1.67‡  (-4.73, 1.38) | **Metformin** | 0.72‡  (-6.66, 7.87) | -3.53*  (-8.80, 1.36) | 1.47‡  (-1.71, 4.42) | -0.12‡  (-2.41, 1.53) |
| -2.65‡  (-10.55, 5.23) | -1.28‡  (-8.70, 6.09) | -1.22‡  (-8.88, 6.34) | 0.44‡  (-6.55, 7.40) | **Topiramate** | -4.28‡  (-12.65, 4.27) | 0.68‡  (-6.57, 8.18) | -0.94‡  (-7.93, 6.07) |
| -0.97‡  (-6.15, 4.23) | 0.39‡  (-3.96, 4.75) | 0.44‡  (-4.24, 5.15) | 2.11‡  (-1.50, 5.74) | 1.67‡  (-6.14, 9.52) | **PHEN/TPM** | 4.93*  (-0.14, 10.34) | 3.33*  (-1.39, 7.99) |
| **-4.53**†  **(-8.39, -0.68)** | **-3.16***  **(-5.79, -0.53)** | -3.12‡  (-6.26, 0.04) | **-1.45**†  **(-2.44, -0.44)** | -1.89‡  (-8.90, 5.14) | -3.55‡  (-7.25, 0.13) | **Sibutramine** | -1.61‡  (-4.16, 0.58) |
| -3.41‡  (-7.17, 0.34) | -2.04†  (-4.53, 0.44) | -2.00†  (-5.02, 1.02) | -0.33‡  (-0.82, 0.16) | -0.77‡  (-7.70, 6.21) | -2.43‡  (-6.03, 1.14) | **1.12**†  **(0.24, 1.99)** | **Control** |

Treatment SBP (MD with 95% Crl) DBP (MD with 95% Crl)

Results of network meta-analysis for SBP and DBP were listed in the left lower half (S1) and upper right half (S2).

S1. SBP (*N*_T_ = 17 [57%], *n*_t_ = 2031 [53%], Bayesian fixed-effects model, *I*^2^ = 0.0%)

S2. DBP (*N*_T_ = 17 [57%], *n*_t_ = 2031 [53%], *I*^2^ = 13.0%)

Comparisons should be read from left to right, and the estimate is in the cell in common between the column-defining treatment and

the row-defining treatment. A mean difference (MD) of less than 0 indicates that the outcome is more likely with treatment (column) than reference (row). Significant results are in bold and underscored. The evidence is graded using the CINeMA system (Confidence in Network Meta-Analysis), an adaptation of the GRADE (Grading of Recommendations, Assessment, Development, and Evaluations) approach for network meta-analysis

*PHEN/TPM* phentermine/topiramate, *Control* placebo or no treatment, *NA* not available

*Moderate quality of evidence. †Low quality of evidence. ‡Very low quality of evidence. *N_T_* total number of trials reporting the outcome (percentage of sample), *n_t_* total number of patients available for the respective outcome (percentage of sample)

**Supplemental Table 13.** Results of network meta-analysis for heart rate

| **Exenatide** |  |  |  |  |  |  |  |
| --- | --- | --- | --- | --- | --- | --- | --- |
| 2.57‡  (-9.04, 13.58) | **Liraglutide** |  |  |  |  |  |  |
| 3.54‡  (-8.36, 14.93) | 0.97‡  (-11.29, 13.36) | **Semaglutide** |  |  |  |  |  |
| 12.57‡  (-1.02, 25.69) | 10.03‡  (-3.82, 23.79) | 9.01‡  (-5.15, 23.15) | **Metformin** |  |  |  |  |
| 6.04‡  (-7.12, 18.74) | 3.48‡  (-9.94, 16.90) | 2.51‡  (-11.31, 16.16) | -6.54‡  (-21.76, 8.74) | **Topiramate** |  |  |  |
| 2.92‡  (-8.87, 14.18) | 0.35‡  (-11.86, 12.74) | -0.61‡  (-13.20, 11.93) | -9.65‡  (-23.65, 4.45) | -3.12‡  (-16.75, 10.61) | **PHEN/TPM** |  |  |
| -0.22‡  (-9.89, 8.00) | -2.62‡  (-13.09, 6.28) | -3.66‡  (-14.50, 5.71) | **-12.78**‡  **(-25.24, -1.32)** | -6.22‡  (-18.27, 4.83) | -3.02‡  (-13.88, 6.23) | **Sibutramine** |  |
| 4.57‡  (-3.51, 11.88) | 2.01‡  (-6.44, 10.51) | 1.04‡  (-7.84, 9.96) | -8.01‡  (-19.00, 3.09) | -1.45‡  (-11.93, 9.11) | 1.66‡  (-7.24, 10.46) | **4.71**‡  **(0.69, 10.17)** | **Control** |

Treatment Heart rate (MD with 95% Crl)

Heart rate (*N*_T_ = 37 [30%], *n*_t_ = 1428 [37%], *I*^2^ = 2.0%)

Comparisons should be read from left to right, and the estimate is in the cell in common between the column-defining treatment and

the row-defining treatment. A mean difference (MD) of less than 0 indicates that the outcome is more likely with treatment (column) than reference (row). Significant results are in bold and underscored. The evidence is graded using the CINeMA system (Confidence in Network Meta-Analysis), an adaptation of the GRADE (Grading of Recommendations, Assessment, Development, and Evaluations) approach for network meta-analysis

*PHEN/TPM* phentermine/topiramate, *Control* placebo or no treatment, *NA* not available

‡Very low quality of evidence. *N_T_* total number of trials reporting the outcome (percentage of sample), *n_t_* total number of patients available for the respective outcome (percentage of sample)

**Supplemental Table 14.** Results of network meta-analysis for depression and gastrointestinal disorders

| **Exenatide** | 0.79‡  (0.08, 7.39) | 1.65‡  (0.03, 80.18) | 2.73‡  (0.37, 20.15) | NA | NA | 3.28‡  (0.31, 34.42) | NA | 2.53‡  (0.55, 11.69) |
| --- | --- | --- | --- | --- | --- | --- | --- | --- |
| NA | **Liraglutide** | 2.10‡  (0.04, 105.76) | 3.46‡  (0.44, 27.57) | NA | NA | 4.16‡  (0.37, 46.57) | NA | 3.20†  (0.63, 16.35) |
| NA | 0.90‡  (0.03, 30.44) | **Semaglutide** | 1.65‡  (0.04, 73.18) | NA | NA | 1.98‡  (0.04, 107.01) | NA | 1.53‡  (0.04, 54.10) |
| NA | NA | NA | **Metformin** | NA | NA | 1.20‡  (0.13, 10.81) | NA | 0.93‡  (0.26, 3.34) |
| NA | 1.52‡  (0.05, 44.12) | 1.69‡  (0.02, 141.50) | NA | **Orlistat** | NA | NA | NA | NA |
| NA | 0.51‡  (0.02, 12.65) | 0.57‡  (0.01, 42.16) | NA | 0.34‡  (0.01, 21.92) | **Topiramate** | NA | NA | NA |
| NA | 2.66‡  (0.11, 67.77) | 2.98‡  (0.04, 224.62) | NA | 1.76‡  (0.03, 116.83) | 5.25‡  (0.09, 308.62) | **PHEN/TPM** | NA | 0.77‡  (0.13, 4.58) |
| NA | 1.25‡  (0.12, 12.66) | 1.39‡  (0.04, 55.62) | NA | 0.82‡  (0.02, 28.28) | 2.46‡  (0.08, 72.95) | 0.47‡  (0.02, 14.17) | **Sibutramine** | NA |
| NA | 0.59†  (0.14, 2.50) | 0.65‡  (0.03, 16.28) | NA | 0.39‡  (0.02, 8.09) | 1.15‡  (0.07, 20.34) | 0.22‡  (0.01, 3.97) | 0.47‡  (0.08, 2.85) | **Control** |

Treatment Depression (OR with 95% Crl) Gastrointestinal disorders (OR with 95% Crl)

Results of network meta-analysis for depression and gastrointestinal disorders were listed in the left lower half (S1) and upper right half (S2).

S1. Depression (*N*_T_ = 7 [23%], *n*_t_ = 1759 [46%], *I*^2^ = 0.0%)

S2. Gastrointestinal disorders (*N*_T_ = 7 [23%], *n*_t_ = 1004 [26%], *I*^2^ = 7.0%)

Comparisons should be read from left to right, and the estimate is in the cell in common between the column-defining treatment and

the row-defining treatment. An OR less than 1 indicates that the outcome is more likely with treatment (column) than reference (row). Significant results are in bold and underscored. The evidence is graded using the CINeMA system (Confidence in Network Meta-Analysis), an adaptation of the GRADE (Grading of Recommendations, Assessment, Development, and Evaluations) approach for network meta-analysis.

*PHEN/TPM* phentermine/topiramate, *Control* placebo or no treatment, *NA* not available

†Low quality of evidence. ‡Very low quality of evidence. *N_T_* total number of trials reporting the outcome (percentage of sample), *n_t_* total number of patients available for the respective outcome (percentage of sample)

Supplemental Table 15. Network meta-analysis results for SAE

| **Exenatide** |  |  |  |  |  |  |  |  |  |  |
| --- | --- | --- | --- | --- | --- | --- | --- | --- | --- | --- |
| 0.37‡  (0.03, 4.80) | **Liraglutide** |  |  |  |  |  |  |  |  |  |
| 0.80‡  (0.08, 8.30) | 2.17‡  (0.37, 12.65) | **Semaglutide** |  |  |  |  |  |  |  |  |
| 0.87‡  (0.08, 9.98) | 2.39‡  (0.36, 15.64) | 1.10‡  (0.23, 5.20) | **Metformin** |  |  |  |  |  |  |  |
| 0.73‡  (0.03, 19.64) | 2.00‡  (0.11, 36.43) | 0.92‡  (0.06, 13.73) | 0.84‡  (0.07, 10.33) | **Fluoxetine** |  |  |  |  |  |  |
| 0.73‡  (0.03, 19.64) | 2.00‡  (0.11, 36.43) | 0.92‡  (0.06, 13.73) | 0.84‡  (0.07, 10.33) | 1.00‡  (0.06, 16.71) | **Met/Flu** |  |  |  |  |  |
| 0.54‡  (0.01, 49.33) | 1.48‡  (0.02, 102.47) | 0.68‡  (0.01, 41.26) | 0.62‡  (0.01, 39.56) | 0.74‡  (0.01, 81.75) | 0.74‡  (0.01, 81.75) | **Topiramate** |  |  |  |  |
| 1.05‡  (0.03, 43.22) | 2.87‡  (0.10, 84.23) | 1.32‡  (0.05, 32.72) | 1.20‡  (0.05, 31.82) | 1.43‡  (0.03, 74.51) | 1.43‡  (0.03, 74.51) | 1.94‡  (0.01, 293.33) | **PHEN/TPM** |  |  |  |
| 0.64‡  (0.06, 6.60) | 1.75‡  (0.31, 10.02) | 0.81‡  (0.20, 3.23) | 0.73‡  (0.16, 3.40) | 0.87‡  (0.06, 12.88) | 0.87‡  (0.06, 12.88) | 1.19‡  (0.02, 71.38) | 0.61‡  (0.03, 14.98) | **Orlistat** |  |  |
| 1.69‡  (0.10, 27.83) | 4.60‡  (0.44, 47.70) | 2.12‡  (0.26, 17.05) | 1.93‡  (0.22, 17.16) | 2.30‡  (0.10, 51.43) | 2.30‡  (0.10, 51.43) | 3.12‡  (0.04, 249.86) | 1.61‡  (0.05, 56.37) | 2.63‡  (0.33, 20.87) | **Sibutramine** |  |
| 0.62‡  (0.07, 5.14) | 1.68†  (0.39, 7.19) | 0.77‡  (0.29, 2.10) | 0.70‡  (0.21, 2.32) | 0.84‡  (0.07, 10.33) | 0.84‡  (0.07, 10.33) | 1.14‡  (0.02, 61.08) | 0.59‡  (0.03, 12.39) | 0.96‡  (0.37, 2.52) | 0.37‡  (0.06, 2.28) | **Control** |

Treatment SAE (OR with 95% Crl)

SAE (*N*_T_ = 20 [67%], *n*_t_ = 3241 [85%], *I*^2^ = 0.0%)

Comparisons should be read from left to right, and the estimate is in the cell in common between the column-defining treatment and

the row-defining treatment. An OR less than 1 indicates that the outcome is more likely with treatment (column) than reference (row). Significant results are in bold and underscored. The evidence is graded using the CINeMA system (Confidence in Network Meta-Analysis), an adaptation of the GRADE (Grading of Recommendations, Assessment, Development, and Evaluations) approach for network meta-analysis.

*Met/Flu* metformin/ fluoxetine, *PHEN/TPM* phentermine/topiramate, *Control* placebo or no treatment, *NA* not available

†Low quality of evidence. ‡Very low quality of evidence. *N_T_* total number of trials reporting the outcome (percentage of sample), *n_t_* total number of patients available for the respective outcome (percentage of sample)

**Appendix 10. Ranking based on simulations for outcome**

**Supplemental Table 16.**

| **Outcome** |  | **PHEN/TPM** | **Semaglutide** | **Exenatide** | **Liraglutide** | **Topiramate** | **Metformin** | **Fluoxetine** | **MET/FLU** | **Sibutramine** | **Orlistat** | **Control** |
| --- | --- | --- | --- | --- | --- | --- | --- | --- | --- | --- | --- | --- |
| **BMI** | SUCRA | 0.97 | 0.93 | 0.44 | 0.50 | 0.32 | 0.26 | 0.52 | 0.62 | 0.45 | 0.46 | 0.04 |
|  | Rank | **1** | **2** | **8** | **5** | **9** | **10** | **4** | **3** | **7** | **6** | **11** |
| **Percent change in BMI** | SUCRA | 0.77 | 0.96 | 0.39 | 0.46 | 0.26 | - | - | - | 0.57 | - | 0.08 |
|  | Rank | **2** | **1** | **5** | **4** | **6** | **-** | **-** | **-** | **3** | **-** | **7** |
| **BMI-SDS** | SUCRA | - | - | 0.47 | 0.90 | - | 0.47 | - | - | 0.59 | - | 0.07 |
|  | Rank | **-** | **-** | **3** | **1** | **-** | **4** | **-** | **-** | **2** | **-** | **5** |
| **Weight** | SUCRA | 0.90 | 0.97 | 0.46 | 0.47 | 0.301 | 0.30 | - | - | 0.49 | 0.57 | 0.05 |
|  | Rank | **2** | **1** | **6** | **5** | **7** | **8** | **-** | **-** | **4** | **3** | **9** |
| **Waist circumference** | SUCRA | 0.89 | 0.99 | 0.38 | 0.57 | - | 0.20 | 0.19 | 0.38 | 0.78 | 0.60 | 0.02 |
|  | Rank | **2** | **1** | **6** | **5** | **-** | **8** | **9** | **7** | **3** | **4** | **10** |
| **FBG** | SUCRA | - | 0.73 | 0.35 | 0.49 | 0.89 | 0.36 | - | - | 0.23 | 0.81 | 0.15 |
|  | Rank | **-** | **3** | **6** | **4** | **1** | **5** | **-** | **-** | **7** | **2** | **8** |
| **FINS** | SUCRA | - | - | 0.35 | - | - | 0.66 | - | - | 0.82 | 0.49 | 0.19 |
|  | Rank | **-** | **-** | **4** | **-** | **-** | **2** | **-** | **-** | **1** | **3** | **5** |
| **HOMA-IR** | SUCRA | - | - | - | - | - | 0.46 | - | - | 0.99 | 0.42 | 0.11 |
|  | Rank | **-** | **-** | **-** | **-** | **-** | **2** | **-** | **-** | **1** | **3** | **4** |
| **TC** | SUCRA | - | - | 0.33 | - | 0.41 | 0.71 | - | - | 0.06 | 0.98 | 0.52 |
|  | Rank | **-** | **-** | **5** | **-** | **4** | **2** | **-** | **-** | **6** | **1** | **3** |
| **LDL-C** | SUCRA | - | - | 0.33 | - | 0.20 | 0.53 | - | - | 0.40 | 0.89 | 0.65 |
|  | Rank | **-** | **-** | **5** | **-** | **6** | **3** | **-** | **-** | **4** | **1** | **2** |
| **HDL-C** | SUCRA | - | - | 0.45 | - | 0.70 | 0.21 | - | - | 0.80 | 0.54 | 0.30 |
|  | Rank | **-** | **-** | **4** | **-** | **2** | **6** | **-** | **-** | **1** | **3** | **5** |
| **TG** | SUCRA | - | - | 0.35 | - | 0.86 | 0.48 | - | - | 0.70 | 0.38 | 0.22 |
|  | Rank | **-** | **-** | **5** | **-** | **1** | **3** | **-** | **-** | **2** | **4** | **6** |
| **SBP** | SUCRA | 0.70 | 0.65 | 0.82 | 0.67 | 0.45 | 0.40 | - | - | 0.05 | - | 0.25 |
|  | Rank | **2** | **4** | **1** | **3** | **5** | **6** | **-** | **-** | **8** | **-** | **7** |
| **DBP** | SUCRA | 0.88 | 0.63 | 0.73 | 0.27 | 0.37 | 0.44 | - | - | 0.17 | - | 0.50 |
|  | Rank | **1** | **3** | **2** | **7** | **6** | **5** | **-** | **-** | **8** | **-** | **4** |
| **HR** | SUCRA | 0.45 | 0.50 | 0.24 | 0.42 | 0.66 | 0.92 | - | - | 0.18 | - | 0.62 |
|  | Rank | **5** | **4** | **7** | **6** | **2** | **1** | **-** | **-** | **8** | **-** | **3** |
| **Depression** | SUCRA | 0.16 | 0.25 | - | 0.69 | 0.80 | - | - | - | 0.57 | 0.20 | 0.82 |
|  | Rank | **7** | **5** | **-** | **3** | **2** | **-** | **-** | **-** | **4** | **6** | **1** |
| **Gastrointestinal disorders** | SUCRA | 0.77 | 0.07 | 0.38 | 0.34 | - | 0.721 | - | - | - | - | 0.72 |
|  | Rank | **1** | **6** | **4** | **5** | **-** | **2** | **-** | **-** | **-** | **-** | **3** |
| **SAE** | SUCRA | 0.15 | 0.57 | 0.22 | 0.71 | 0.72 | 0.45 | 0.40 | 0.62 | 0.39 | 0.64 | 0.63 |
|  | Rank | **11** | **6** | **10** | **2** | **1** | **7** | **8** | **5** | **9** | **3** | **4** |

|  |  |  |  |  |  |  |  |  |  |  |  |
| --- | --- | --- | --- | --- | --- | --- | --- | --- | --- | --- | --- |

**Best treatment Worst treatment No data**

Ranking is the probability of being the best treatment, the second best treatment, the third best treatment, and so on, out of all treatments

**Abbreviation**: SUCRA: surface under the cumulative ranking curve; MET/FLU: metformin/ fluoxetine; PHEN/TPM: phentermine/topiramate; Control: placebo or no treatment

**Appendix 11. Subgroup network meta-analyses**

**Supplemental Table 17.** Subgroup network meta-analyses for BMI

| **Comparison** | **Exenatide vs. Control** | **Fluoxetine vs.**  **Control** | **Liraglutide vs.**  **Control** | **Met/Flu vs. Control** | **Metformin vs. Control** | **Orlistat vs. Control** | **PHEN/TPM vs. Control** | **Semaglutide vs. Control** | **Sibutramine vs. Control** |
| --- | --- | --- | --- | --- | --- | --- | --- | --- | --- |
| **Overall**  (*N*_T_= 29, *n*_t_= 3224,  *I*^2^ = 0.0%) | -1.33  (-2.83, 0.15) | **-1.57**  **(-2.82, -0.35)** | -1.57  (-3.70, 0.56) | **-1.87**  **(-3.12, -0.65)** | **-0.85**  **(-1.49, -0.25)** | **-1.38**  **(-2.37, -0.44)** | **-6.96**  **(-10.44, -3.43)** | **-5.90**  **(-8.13, -3.65)** | **-1.37**  **(-2.74, -0.02)** |
| **BMI ≥35 kg/m^2^**  (*N*_T_= 16, *n*_t_= 2063, *I*^2^ = 0.0%) | **-1.29**  **(-2.42, -0.24)** | - | **-1.59**  **(-3.04, -0.10)** | - | **-1.46**  **(-2.35, -0.50)** | -0.75  (-1.72, 0.33) | **-6.89**  **(-10.03, -4.02)** | **-5.91**  **(-7.53, -4.26)** | **-1.89**  **(-3.22, -0.61)** |
| **BMI<35 kg/m^2^**  (*N*_T_= 12, *n*_t_= 961,  *I*^2^ = 0.0%) | - | -0.80  (-3.56, 1.98) | - | -1.10  (-3.84, 1.67) | -0.91  (-1.96, 0.11) | **-2.47**  **(-4.70, -0.47)** | - | - | -0.40  (-3.38, 2.58) |

Supplemental Table 17. Subgroup network meta-analyses for BMI continued

| **Comparison** | **Topiramate vs. Control** | **Fluoxetine vs.**  **Exenatide** | **Liraglutide vs.**  **Exenatide** | **Met/Flu vs.**  **Exenatide** | **Metformin vs. Exenatide** | **Orlistat vs.**  **Exenatide** | **PHEN/TPM vs. Exenatide** | **Semaglutide vs. Exenatide** | **Sibutramine vs.**  **Exenatide** |
| --- | --- | --- | --- | --- | --- | --- | --- | --- | --- |
| **Overall** | -0.87  (-3.21, 1.48) | -0.24  (-2.18, 1.69) | -0.24  (-2.83, 2.36) | -0.55  (-2.48, 1.39) | 0.48  (-1.14, 2.09) | -0.05  (-1.84, 1.72) | **-5.62**  **(-9.42, -1.79)** | **-4.57**  **(-7.25, -1.86)** | -0.04  (-2.05, 1.97) |
| **BMI ≥35 kg/m^2^** | -0.86  (-2.62, 0.90) | - | -0.29  (-2.07, 1.59) | - | -0.17  (-1.52, 1.34) | 0.54  (-0.86, 2.13) | **-5.59**  **(-8.88, -2.51)** | **-4.62**  **(-6.53, -2.60)** | -0.60  (-2.27, 1.11) |
| **BMI<35 kg/m^2^** | - | - | - | - | - | - | - | - | - |

Supplemental Table 17. Subgroup network meta-analyses for BMI continued

| **Comparison** | **Topiramate vs. Exenatide** | **Liraglutide vs. Fluoxetine** | **Met/Flu vs.**  **Fluoxetine** | **Metformin vs. Fluoxetine** | **Orlistat vs.**  **Fluoxetine** | **PHEN/TPM vs. Fluoxetine** | **Semaglutide vs. Fluoxetine** | **Sibutramine vs. Fluoxetine** | **Topiramate vs.**  **Fluoxetine** |
| --- | --- | --- | --- | --- | --- | --- | --- | --- | --- |
| **Overall** | 0.47  (-2.32, 3.25) | 0.00  (-2.45, 2.47) | -0.30  (-1.68, 1.08) | 0.73  (-0.52, 1.95) | 0.19  (-1.39, 1.74) | **-5.38**  **(-9.08, -1.65)** | **-4.33**  **(-6.86, -1.76)** | 0.21  (-1.62, 2.03) | 0.71  (-1.93, 3.38) |
| **BMI ≥35 kg/m^2^** | 0.43  (-1.62, 2.55) | - | - | - | - | - | - | - | - |
| **BMI<35 kg/m^2^** | - | - | -0.30  (-4.21, 3.57) | -0.11  (-3.09, 2.81) | -1.66  (-5.29, 1.69) | - | - | 0.40  (-3.69, 4.46) | - |

Supplemental Table 17. Subgroup network meta-analyses for BMI continued

| **Comparison** | **Met/Flu vs.**  **Liraglutide** | **Metformin vs. Liraglutide** | **Orlistat vs.**  **Liraglutide** | **PHEN/TPM vs. Liraglutide** | **Semaglutide vs. Liraglutide** | **Sibutramine vs. Liraglutide** | **Topiramate vs.**  **Liraglutide** | **Metformin vs.**  **Met/Flu** | **Orlistat vs.**  **Met/Flu** |
| --- | --- | --- | --- | --- | --- | --- | --- | --- | --- |
| **Overall** | -0.30  (-2.78, 2.16) | 0.73  (-1.52, 2.92) | 0.20  (-2.17, 2.50) | **-5.38**  **(-9.45, -1.28)** | **-4.32**  **(-7.39, -1.24)** | 0.21  (-2.33, 2.71) | 0.71  (-2.47, 3.88) | 1.03  (-0.22, 2.25) | 0.49  (-1.09, 2.04) |
| **BMI ≥35 kg/m^2^** | - | 0.12  (-1.56, 1.90) | 0.84  (-0.89, 2.67) | **-5.30**  **(-8.77, -2.10)** | **-4.32**  **(-6.51, -2.14)** | -0.31  (-2.29, 1.61) | 0.72  (-1.57, 3.02) | - | - |
| **BMI<35 kg/m^2^** | - | - | - | - | - | - | - | 0.19  (-2.77, 3.10) | -1.36  (-4.98, 1.97) |

Supplemental Table 17. Subgroup network meta-analyses for BMI continued

| **Comparison** | **PHEN/TPM vs. Met/Flu** | **Semaglutide vs. Met/Flu** | **Sibutramine vs. Met/Flu** | **Topiramate vs. Met/Flu** | **Orlistat vs.**  **Metformin** | **PHEN/TPM vs. Metformin** | **Semaglutide vs. Metformin** | **Sibutramine vs. Metformin** | **Topiramate vs.**  **Metformin** |
| --- | --- | --- | --- | --- | --- | --- | --- | --- | --- |
| **Overall** | **-5.08**  **(-8.76, -1.34)** | **-4.02**  **(-6.57, -1.45)** | 0.51  (-1.34, 2.33) | 1.01  (-1.64, 3.70) | -0.53  (-1.68, 0.60) | **-6.11**  **(-9.63, -2.53)** | **-5.05**  **(-7.34, -2.70)** | -0.52  (-2.00, 0.97) | -0.02  (-2.43, 2.44) |
| **BMI ≥35 kg/m^2^** | - | - | - | - | 0.72  (-0.66, 2.07) | **-5.42**  **(-8.71, -2.47)** | **-4.45**  **(-6.36, -2.62)** | -0.44  (-2.10, 1.09) | 0.58  (-1.43, 2.54) |
| **BMI<35 kg/m^2^** | - | - | 0.70  (-3.35, 4.74) | - | -1.56  (-4.02, 0.68) | - | - | 0.51  (-2.63, 3.68) | - |

Supplemental Table 17. Subgroup network meta-analyses for BMI continued

| **Comparison** | **PHEN/TPM vs. Orlistat** | **Semaglutide vs. Orlistat** | **Sibutramine vs. Orlistat** | **Topiramate vs. Orlistat** | **Semaglutide vs. PHEN/TPM** | **Sibutramine vs. PHEN/TPM** | **Topiramate vs.**  **PHEN/TPM** | **Sibutramine vs. Semaglutide** | **Topiramate vs.**  **Semaglutide** |
| --- | --- | --- | --- | --- | --- | --- | --- | --- | --- |
| **Overall** | **-5.57**  **(-9.18, -1.92)** | **-4.52**  **(-6.93, -2.04)** | 0.01  (-1.63, 1.69) | 0.52  (-2.01, 3.08) | 1.06  (-3.09, 5.20) | **5.59**  **(1.81, 9.30)** | **6.09**  **(1.88, 10.29)** | **4.53**  **(1.88, 7.14)** | **5.03**  **(1.79, 8.28)** |
| **BMI ≥35 kg/m2** | **-6.15**  **(-9.45, -3.15)** | **-5.16**  **(-7.09, -3.29)** | -1.15  (-2.85, 0.42) | -0.13  (-2.17, 1.85) | 0.99  (-2.31, 4.51) | **5.00**  **(1.78, 8.36)** | **6.04**  **(2.65, 9.63)** | **4.02**  **(1.91, 6.08)** | **5.04**  **(2.64, 7.44)** |
| **BMI<35 kg/m2** | - | - | 2.08  (-1.45, 5.84) | - | - | - | - | - | - |

Supplemental Table 17. Subgroup network meta-analyses for BMI continued

| **Comparison** | **Topiramate vs. Sibutramine** |  |  |  |  |  |  |  |  |
| --- | --- | --- | --- | --- | --- | --- | --- | --- | --- |
| **Overall** | 0.50  (-2.20, 3.23) |  |  |  |  |  |  |  |  |
| **BMI ≥35 kg/m^2^** | 1.04  (-1.15, 3.24) |  |  |  |  |  |  |  |  |
| **BMI<35 kg/m^2^** | - |  |  |  |  |  |  |  |  |

For comparisons, MD (95% credible interval) is in cells between treatments. Bold and underlined cells are significant.

*BMI* body-mass index, *Met/Flu* metformin/ fluoxetine, *PHEN/TPM* phentermine/topiramate, *Control* placebo or no treatment. *N_T_* total number of trials reporting the outcome, *n_t_* total number of patients available for the respective outcome.

**Appendix 12. Sensitivity analyses**

Supplemental Table 18. Results of the sensitivity analysis for BMI by excluding open-label trials (26 trials, 3115 patients, *I*^2^ = 0.0%)

| **Exenatide** |  |  |  |  |  |  |  |  |  |  |
| --- | --- | --- | --- | --- | --- | --- | --- | --- | --- | --- |
| 0.25  (-2.12, 2.62) | **Liraglutide** |  |  |  |  |  |  |  |  |  |
| **4.56**  **(2.10, 7.03)** | **4.31**  **(1.50, 7.14)** | **Semaglutide** |  |  |  |  |  |  |  |  |
| -0.64  (-2.12, 0.90) | -0.89  (-2.89, 1.17) | **-5.20**  **(-7.33, -3.04)** | **Metformin** |  |  |  |  |  |  |  |
| 0.16  (-1.58, 1.94) | -0.09  (-2.30, 2.16) | **-4.40**  **(-6.72, -2.04)** | 0.81  (-0.31, 1.89) | **Fluoxetine** |  |  |  |  |  |  |
| 0.46  (-1.28, 2.24) | 0.22  (-2.00, 2.46) | **-4.10**  **(-6.44, -1.75)** | 1.11  (-0.01, 2.19) | 0.30  (-0.92, 1.52) | **Met/Flu** |  |  |  |  |  |
| -0.47  (-3.04, 2.10) | -0.72  (-3.62, 2.19) | **-5.04**  **(-8.03, -2.04)** | 0.17  (-2.11, 2.39) | -0.63  (-3.08, 1.79) | -0.93  (-3.38, 1.48) | **Topiramate** |  |  |  |  |
| **5.70**  **(2.07, 9.31)** | **5.45**  **(1.55, 9.30)** | 1.14  (-2.81, 5.05) | **6.33**  **(2.88, 9.71)** | **5.53**  **(1.98, 9.03)** | **5.23**  **(1.67, 8.73)** | **6.17**  **(2.15, 10.15)** | **PHEN/TPM** |  |  |  |
| -0.40  (-2.07, 1.27) | -0.65  (-2.80, 1.49) | **-4.96**  **(-7.23, -2.69)** | 0.24  (-0.92, 1.33) | -0.56  (-2.02, 0.87) | -0.86  (-2.33, 0.56) | 0.07  (-2.29, 2.44) | **-6.10**  **(-9.56, -2.59)** | **Orlistat** |  |  |
| 0.03  (-1.83, 1.90) | -0.23  (-2.51, 2.11) | **-4.54**  **(-6.93, -2.11)** | 0.66  (-0.74, 2.04) | -0.14  (-1.80, 1.53) | -0.44  (-2.10, 1.23) | 0.49  (-1.99, 3.02) | **-5.67**  **(-9.24, -2.06)** | 0.42  (-1.13, 2.01) | **Sibutramine** |  |
| -1.33  (-2.70, 0.05) | -1.58  (-3.51, 0.36) | **-5.89**  **(-7.95, -3.84)** | **-0.69**  **(-1.33, -0.10)** | **-1.49**  **(-2.61, -0.41)** | **-1.79**  **(-2.91, -0.71)** | -0.86  (-3.02, 1.31) | **-7.03**  **(-10.36, -3.64)** | -0.93  (-1.87, 0.01) | **-1.35**  **(-2.62, -0.12)** | **Control** |

Comparisons should be read from left to right, and the estimate is in the cell in common between the column-defining treatment and

the row-defining treatment. A mean difference (MD) of less than 0 indicates that the outcome is more likely with treatment (column) than reference (row). Significant results are in bold and underscored.

*BMI* body-mass index, *Met/Flu* metformin/ fluoxetine, *PHEN/TPM* phentermine/topiramate, *Control* placebo

**Appendix 13. Meta-regression for network meta-analyses**

**Supplemental Table 19.** Network meta-regression results on BMI

| **Covariate** | **Drug** | **MD drug vs. Control** | **B (beta) of comparison** | | |
| --- | --- | --- | --- | --- | --- |
|  |  | **MD (95% CI)** | **2.5%** | **median** | **97.5%** |
| **Age** | Exenatide | -0.66 (-3.93, 2.68) | -6.50 | -1.19 | 3.96 |
|  | Fluoxetine | -1.72 (-4.37, 1.35) | -17.49 | -1.87 | 17.67 |
|  | Liraglutide | -1.67 (-43.84, 37.66) | -141.81 | 0.26 | 152.55 |
|  | Met/Flu | -1.67 (-3.11, -0.08) | -3.80 | 1.13 | 9.27 |
|  | Metformin | -0.76 (-1.49, -0.08) | -0.87 | 0.29 | 1.36 |
|  | Orlistat | -1.76 (-2.85, -0.73) | -0.28 | 2.53 | 5.28 |
|  | PHEN/TPM | -6.97 (-12.12, -1.70) | -50.99 | -0.19 | 46.80 |
|  | Semaglutide | -5.73 (-21.40, 10.41) | -26.42 | -0.28 | 24.92 |
|  | Sibutramine | -1.58 (-3.28, 0.10) | -4.41 | 1.17 | 6.83 |
|  | Topiramate | -0.54 (-38.28, 43.94) | -82.82 | -0.55 | 68.73 |
| **Follow-up** | Exenatide | -1.37 (-2.98, 0.23) | -3.09 | -0.55 | 1.99 |
|  | Fluoxetine | -2.92 (-7.55, 3.78) | -0.91 | 5.23 | 16.97 |
|  | Liraglutide | -2.04 (-32.59, 13.64) | -22.06 | 0.91 | 36.17 |
|  | Met/Flu | -12.70 (-4.74, 5.39) | -28.04 | -11.81 | 12.00 |
|  | Metformin | -1.00 (-1.74, -0.27) | -1.95 | 0.17 | 2.11 |
|  | Orlistat | -1.38 (-2.45, -0.36) | -2.70 | -0.64 | 1.24 |
|  | PHEN/TPM | -7.95 (-77.25, 24.10) | -22.39 | 0.69 | 60.41 |
|  | Semaglutide | -4.19 (-34.66, 41.72) | -18.24 | -0.11 | 32.33 |
|  | Sibutramine | -1.53 (-2.98, -0.07) | -4.22 | -1.69 | 0.82 |
|  | Topiramate | -0.79 (-10.62, 22.36) | -40.00 | 0.35 | 81.86 |
| **BMI** | Exenatide | -1.64 (-5.24, 2.17) | -5.24 | -1.64 | 2.17 |
|  | Fluoxetine | -3.87 (-14.50, 8.11) | -14.50 | -3.87 | 8.11 |
|  | Liraglutide | -1.36 (-9.07, 12.33) | -9.07 | -1.36 | 12.33 |
|  | Met/Flu | -8.20 (-33.18, 2.43) | -33.18 | -8.20 | 2.43 |
|  | Metformin | -1.00 (-1.73, 0.52) | -1.73 | -1.00 | 0.52 |
|  | Orlistat | -1.46 (-3.18, -0.07) | **-3.18** | **-1.46** | **-0.07** |
|  | PHEN/TPM | -7.09 (-29.49, 8.62) | -29.49 | -7.09 | 8.62 |
|  | Semaglutide | -5.07 (-22.58, 54.81) | -22.58 | -5.07 | 54.81 |
|  | Sibutramine | -1.07 (-3.16, 0.85) | -3.16 | -1.07 | 0.85 |
|  | Topiramate | 0.21 (-16.21, 47.43) | -16.21 | 0.21 | 47.43 |

*Met/Flu* metformin/ fluoxetine, *PHEN/TPM* phentermine/topiramate, *Control* placebo or no treatment.

**Appendix 14. Grading the evidence for the network meta-analysis using CINeMA**

Our conclusions are based on the recommendations of CINeMA's online documentation (http://cinema.ispm.ch/#doc). We graded each network estimate using the criteria listed below.

1. Study limitations:

For each relative efficacy estimate, we use a weighted average score based on the percentage contribution of studies at each bias level.

For instance, you can compute a weighted average of the risk of bias by allocating scores of -1, 0, and 1 to low, moderate, and high risk of bias, respectively.

For the comparison exercise A versus B, this would produce a weighted score of 0.44 × -1 + 0.32 × 0 + 0.24 × 1 = -0.20, which corresponds to’ some concerns’.

1. Across-study bias (publication bias):

We evaluated overall publication bias using the comparison-adjusted funnel plot. though our search includes everything from research registries to drug registries, the comparative adjustment funnel plot for our primary outcome, BMI, appeared to be asymmetrical, as did the other outcome measures, DBP, HR, HOMA-IR,TC, and TG.

1. Imprecision:

The treatment effects contained in the 95% confidence interval are compared to the range of equivalency determined by CINeMA. When the confidence interval crossed the threshold, the comparison degraded by one level. The clinically relevant threshold was chosen at standardised mean differences of -0.1 and 0.1 for continuous outcomes and odds ratios of 0.80 and 1.25 for dichotomous outcomes for comparisons of two treatments. Every time the confidence interval crossed one criterion, we demoted the comparison one level. Crossing both thresholds resulted in a two-level downgrade.

**Risk of bias contributions**

**Supplemental Figure 4a.** Risk of bias assessment for BMI

**Supplemental Figure 4b.** Risk of bias assessment for BMI percentage change

**Supplemental Figure 4c.** Risk of bias assessment for BMI-SDS

**Supplemental Figure 4d.** Risk of bias assessment for weight

**Supplemental Figure 4e.** Risk of bias assessment for waist circumference

**Supplemental Figure 4f.** Risk of bias assessment for TC

**Supplemental Figure 4g.** Risk of bias assessment for LDL-C

**Supplemental Figure 4h.** Risk of bias assessment for HDL-C

**Supplemental Figure 4i.** Risk of bias assessment for TG

**Supplemental Figure 4j.** Risk of bias assessment for FBG

**Supplemental Figure 4k.** Risk of bias assessment for FINS

**Supplemental Figure 4l.** Risk of bias assessment for HOMA-IR

**Supplemental Figure 4m.** Risk of bias assessment for SBP

**Supplemental Figure 4n.** Risk of bias assessment for DBP

**Supplemental Figure 4o.** Risk of bias assessment for heart rate

**Supplemental Figure 4p.** Risk of bias assessment for depression

**Supplemental Figure 4q.** Risk of bias assessment for gastrointestinal disorders

**Supplemental Figure 4r.** Risk of bias assessment for SAE

The network estimates a relative treatment effect for each bar. White vertical lines separate the percentage contributions of different studies. Each bar shows the percentage contribution from studies judged to be at low (green), moderate (yellow), and high (red) risk of bias.

*Met/Flu* metformin/ fluoxetine, *PHEN/TPM* phentermine/topiramate, *Control* placebo or no treatment

**GRADE assessment on quality of evidence**

Supplemental Table 20a. CINeMA ratings for all comparisons of BMI

| **Comparison** | **Nature**  **of**  **evidence** | **Confidence**  **level** | **Downgrading** |
| --- | --- | --- | --- |
| Exenatide vs. Control | Mixed | Very low | Study limitations, Reporting bias |
| Fluoxetine vs. Control | Mixed | Very low | Study limitations, Reporting bias |
| Liraglutide vs. Control | Mixed | Moderate | Reporting bias |
| Met/Flu vs. Control | Mixed | Very low | Study limitations, Reporting bias |
| Metformin vs. Control | Mixed | Very low | Study limitations, Serious Imprecision |
| Orlistat vs. Control | Mixed | Moderate | Study limitations, Reporting bias |
| PHEN/TPM vs. Control | Mixed | Very low | Study limitations, Reporting bias |
| Semaglutide vs.Control | Mixed | Moderate | Study limitations, Reporting bias |
| Sibutramine vs. Control | Mixed | Very low | Study limitations, Reporting bias |
| Topiramate vs. Control | Mixed | Very low | Study limitations, Reporting bias, Serious Imprecision |
| Fluoxetine vs. Met/Flu | Mixed | Very low | Study limitations, Reporting bias |
| Fluoxetine vs. Metformin | Mixed | Very low | Study limitations, Reporting bias |
| Met/Flu vs. Metformin | Mixed | Very low | Study limitations, Reporting bias |
| Exenatide vs. Fluoxetine | Indirect | Very low | Study limitations, Reporting bias, Serious Imprecision |
| Exenatide vs. Liraglutide | Indirect | Very low | Study limitations, Reporting bias, Serious Imprecision |
| Exenatide vs. Met/Flu | Indirect | Very low | Study limitations, Reporting bias, Serious Imprecision |
| Exenatide vs. Metformin | Indirect | Very low | Study limitations, Reporting bias |
| Exenatide vs. Orlistat | Indirect | Very low | Study limitations, Reporting bias, Serious Imprecision |
| Exenatide vs. PHEN/TPM | Indirect | Very low | Study limitations, Reporting bias |
| Exenatide vs. Semaglutide | Indirect | Moderate | Study limitations, Reporting bias |
| Exenatide vs. Sibutramine | Indirect | Very low | Study limitations, Reporting bias, Serious Imprecision |
| Exenatide vs. Topiramate | Indirect | Very low | Study limitations, Reporting bias, Serious Imprecision |
| Fluoxetine vs. Liraglutide | Indirect | Very low | Study limitations, Reporting bias, Serious Imprecision |
| Fluoxetine vs. Orlistat | Indirect | Very low | Study limitations, Reporting bias, Serious Imprecision |
| Fluoxetine vs. PHEN/TPM | Indirect | Low | Study limitations, Reporting bias |
| Fluoxetine vs. Semaglutide | Indirect | Very low | Study limitations, Reporting bias |
| Fluoxetine vs. Sibutramine | Indirect | Very low | Study limitations, Reporting bias, Serious Imprecision |
| Fluoxetine vs. Topiramate | Indirect | Very low | Study limitations, Reporting bias, Serious Imprecision |
| Liraglutide vs. Met/Flu | Indirect | Very low | Study limitations, Reporting bias, Serious Imprecision |
| Liraglutide vs. Metformin | Indirect | Moderate | Study limitations, Reporting bias |
| Liraglutide vs. Orlistat | Indirect | Very low | Study limitations, Reporting bias, Serious Imprecision |
| Liraglutide vs. PHEN/TPM | Indirect | Moderate | Study limitations, Reporting bias |
| Liraglutide vs. Semaglutide | Indirect | Moderate | Study limitations, Reporting bias |
| Liraglutide vs. Sibutramine | Indirect | Very low | Study limitations, Reporting bias, Serious Imprecision |
| Liraglutide vs. Topiramate | Indirect | Very low | Study limitations, Reporting bias, Serious Imprecision |
| Met/Flu vs. Orlistat | Indirect | Very low | Study limitations, Reporting bias, Serious Imprecision |
| Met/Flu vs. PHEN/TPM | Indirect | Very low | Study limitations, Reporting bias |
| Met/Flu vs. Semaglutide | Indirect | Very low | Study limitations, Reporting bias |
| Met/Flu vs. Sibutramine | Indirect | Very low | Study limitations, Reporting bias, Serious Imprecision |
| Met/Flu vs. Topiramate | Indirect | Very low | Study limitations, Reporting bias, Serious Imprecision |
| Metformin vs. Orlistat | Indirect | Very low | Study limitations, Reporting bias |
| Metformin vs. PHEN/TPM | Indirect | Very low | Study limitations, Reporting bias |
| Metformin vs. Semaglutide | Indirect | Very low | Study limitations, Reporting bias |
| Metformin vs. Sibutramine | Indirect | Very low | Study limitations, Reporting bias |
| Metformin vs. Topiramate | Indirect | Very low | Study limitations, Reporting bias, Serious Imprecision |
| Orlistat vs. PHEN/TPM | Indirect | Very low | Study limitations, Reporting bias |
| Orlistat vs. Semaglutide | Indirect | Moderate | Study limitations, Reporting bias |
| Orlistat vs. Sibutramine | Indirect | Very low | Study limitations, Reporting bias, Serious Imprecision |
| Orlistat vs. Topiramate | Indirect | Very low | Study limitations, Reporting bias, Serious Imprecision |
| PHEN/TPM vs. Semaglutide | Indirect | Very low | Study limitations, Reporting bias, Serious Imprecision |
| PHEN/TPM vs. Sibutramine | Indirect | Very low | Study limitations, Reporting bias |
| PHEN/TPM vs. Topiramate | Indirect | Very low | Study limitations, Reporting bias |
| Semaglutide vs. Sibutramine | Indirect | Moderate | Study limitations, Reporting bias |
| Semaglutide vs. Topiramate | Indirect | Very low | Study limitations, Reporting bias |
| Sibutramine vs. Topiramate | Indirect | Very low | Study limitations, Reporting bias, Serious Imprecision |

**Supplemental Table 20b.** CINeMA ratings for all comparisons of BMI percentage change

| **Comparison** | **Nature**  **of**  **evidence** | **Confidence**  **level** | **Downgrading** |
| --- | --- | --- | --- |
| Exenatide vs. Control | Mixed | Low | Study limitations |
| Liraglutide vs. Control | Mixed | High |  |
| PHEN/TPM vs. Control | Mixed | Low | Study limitations |
| Semaglutide vs.Control | Mixed | Moderate | Study limitations |
| Sibutramine vs. Control | Mixed | Low | Study limitations |
| Topiramate vs. Control | Mixed | Very low | Study limitations, Serious Imprecision |
| Exenatide vs. Liraglutide | Indirect | Very low | Study limitations, Serious Imprecision |
| Exenatide vs. PHEN/TPM | Indirect | Very low | Study limitations, Serious Imprecision |
| Exenatide vs. Semaglutide | Indirect | Low | Study limitations |
| Exenatide vs. Sibutramine | Indirect | Very low | Study limitations, Serious Imprecision |
| Exenatide vs. Topiramate | Indirect | Very low | Study limitations, Serious Imprecision |
| Liraglutide vs. PHEN/TPM | Indirect | Very low | Study limitations, Serious Imprecision |
| Liraglutide vs. Semaglutide | Indirect | Moderate | Study limitations |
| Liraglutide vs. Sibutramine | Indirect | Very low | Study limitations, Serious Imprecision |
| Liraglutide vs. Topiramate | Indirect | Very low | Study limitations, Serious Imprecision |
| PHEN/TPM vs. Semaglutide | Indirect | Very low | Study limitations, Serious Imprecision |
| PHEN/TPM vs. Sibutramine | Indirect | Very low | Study limitations, Serious Imprecision |
| PHEN/TPM vs. Topiramate | Indirect | Low | Study limitations |
| Semaglutide vs. Sibutramine | Indirect | Moderate | Study limitations |
| Semaglutide vs. Topiramate | Indirect | Low | Study limitations |
| Sibutramine vs. Topiramate | Indirect | Very low | Study limitations, Serious Imprecision |

Supplemental Table 20c. CINeMA ratings for all comparisons of BMI-SDS

| **Comparison** | **Nature**  **of**  **evidence** | **Confidence**  **level** | **Downgrading** |
| --- | --- | --- | --- |
| Exenatide vs. Control | Mixed | Low | Serious Imprecision |
| Liraglutide vs.Control | Mixed | High |  |
| Metformin vs.Control | Mixed | Moderate | Study limitations |
| Sibutramine vs. Control | Mixed | Very low | Study limitations, Serious Imprecision |
| Exenatide vs. Liraglutide | Indirect | Low | Serious Imprecision |
| Exenatide vs. Metformin | Indirect | Low | Serious Imprecision |
| Exenatide vs. Sibutramine | Indirect | Very low | Study limitations, Serious Imprecision |
| Liraglutide vs. Metformin | Indirect | Low | Study limitations |
| Liraglutide vs. Sibutramine | Indirect | Very low | Study limitations, Serious Imprecision |
| Metformin vs. Sibutramine | Indirect | Very low | Study limitations, Serious Imprecision |

Supplemental Table 20d. CINeMA ratings for all comparisons of weight

| **Comparison** | **Nature**  **of**  **evidence** | **Confidence**  **level** | **Downgrading** |
| --- | --- | --- | --- |
| Exenatide vs. Control | Mixed | Very low | Study limitations, Serious Imprecision |
| Liraglutide vs. Control | Mixed | Low | Serious Imprecision |
| Metformin vs. Control | Mixed | Low | Study limitations |
| Orlistat vs. Control | Mixed | Moderate | Study limitations |
| PHEN/TPM vs. Control | Mixed | Low | Study limitations, Serious Imprecision |
| Semaglutide vs. Control | Mixed | Moderate | Study limitations |
| Sibutramine vs. Control | Mixed | Low | Study limitations |
| Topiramate vs. Control | Mixed | Very low | Study limitations |
| Exenatide vs. Liraglutide | Indirect | Very low | Study limitations, Serious Imprecision |
| Exenatide vs. Metformin | Indirect | Very low | Study limitations, Serious Imprecision |
| Exenatide vs. Orlistat | Indirect | Very low | Study limitations, Serious Imprecision |
| Exenatide vs. PHEN/TPM | Indirect | Low | Study limitations |
| Exenatide vs. Semaglutide | Indirect | Moderate | Study limitations |
| Exenatide vs. Sibutramine | Indirect | Very low | Study limitations, Serious Imprecision |
| Exenatide vs. Topiramate | Indirect | Very low | Study limitations, Serious Imprecision |
| Liraglutide vs. Metformin | Indirect | Very low | Study limitations, Serious Imprecision |
| Liraglutide vs. Orlistat | Indirect | Very low | Study limitations, Serious Imprecision |
| Liraglutide vs. PHEN/TPM | Indirect | Moderate | Study limitations |
| Liraglutide vs. Semaglutide | Indirect | Moderate | Study limitations |
| Liraglutide vs. Sibutramine | Indirect | Very low | Study limitations, Serious Imprecision |
| Liraglutide vs. Topiramate | Indirect | Very low | Study limitations, Serious Imprecision |
| Metformin vs. Orlistat | Indirect | Very low | Study limitations, Serious Imprecision |
| Metformin vs. PHEN/TPM | Indirect | Low | Study limitations |
| Metformin vs. Semaglutide | Indirect | Moderate | Study limitations |
| Metformin vs. Sibutramine | Indirect | Very low | Study limitations, Serious Imprecision |
| Metformin vs. Topiramate | Indirect | Very low | Study limitations, Serious Imprecision |
| Orlistat vs. PHEN/TPM | Indirect | Low | Study limitations |
| Orlistat vs. Semaglutide | Indirect | Moderate | Study limitations |
| Orlistat vs. Sibutramine | Indirect | Very low | Study limitations, Serious Imprecision |
| Orlistat vs. Topiramate | Indirect | Very low | Study limitations, Serious Imprecision |
| PHEN/TPM vs. Semaglutide | Indirect | Very low | Study limitations, Serious Imprecision |
| PHEN/TPM vs. Sibutramine | Indirect | Low | Study limitations |
| PHEN/TPM vs. Topiramate | Indirect | Low | Study limitations |
| Semaglutide vs. Sibutramine | Indirect | Moderate | Study limitations |
| Semaglutide vs. Topiramate | Indirect | Low | Study limitations |
| Sibutramine vs. Topiramate | Indirect | Very low | Study limitations, Serious Imprecision |

**Supplemental Table 20e.** CINeMA ratings for all comparisons of waist circumference

| **Comparison** | **Nature**  **of**  **evidence** | **Confidence**  **level** | **Downgrading** |
| --- | --- | --- | --- |
| Exenatide vs. Control | Mixed | Very low | Study limitations, Serious Imprecision |
| Fluoxetine vs. Control | Mixed | Low | Study limitations |
| Liraglutide vs. Control | Mixed | High |  |
| Met/Flu vs. Control | Mixed | Low | Study limitations |
| Metformin vs. Control | Mixed | Low | Study limitations |
| Orlistat vs. Control | Mixed | Moderate | Study limitations |
| PHEN/TPM vs. Control | Mixed | Low | Study limitations |
| Semaglutide vs. Control | Mixed | Moderate | Study limitations |
| Sibutramine vs. Control | Mixed | Low | Study limitations |
| Fluoxetine vs. Met/Flu | Mixed | Low | Study limitations |
| Fluoxetine vs. Metformin | Mixed | Very low | Study limitations, Serious Imprecision |
| Met/Flu vs. Metformin | Mixed | Low | Study limitations |
| Exenatide vs. Fluoxetine | Indirect | Very low | Study limitations, Serious Imprecision |
| Exenatide vs. Liraglutide | Indirect | Very low | Study limitations, Serious Imprecision |
| Exenatide vs. Met/Flu | Indirect | Very low | Study limitations, Serious Imprecision |
| Exenatide vs. Metformin | Indirect | Very low | Study limitations, Serious Imprecision |
| Exenatide vs. Orlistat | Indirect | Very low | Study limitations, Serious Imprecision |
| Exenatide vs. PHEN/TPM | Indirect | Low | Study limitations |
| Exenatide vs. Semaglutide | Indirect | Moderate | Study limitations |
| Exenatide vs. Sibutramine | Indirect | Low | Study limitations |
| Fluoxetine vs. Liraglutide | Indirect | Moderate | Study limitations |
| Fluoxetine vs. Orlistat | Indirect | Low | Study limitations |
| Fluoxetine vs. PHEN/TPM | Indirect | Low | Study limitations |
| Fluoxetine vs. Semaglutide | Indirect | Low | Study limitations |
| Fluoxetine vs. Sibutramine | Indirect | Low | Study limitations |
| Liraglutide vs. Met/Flu | Indirect | Very low | Study limitations, Serious Imprecision |
| Liraglutide vs. Metformin | Indirect | Moderate | Study limitations |
| Liraglutide vs. Orlistat | Indirect | Very low | Study limitations, Serious Imprecision |
| Liraglutide vs. PHEN/TPM | Indirect | Moderate | Study limitations |
| Liraglutide vs. Semaglutide | Indirect | Moderate | Study limitations |
| Liraglutide vs. Sibutramine | Indirect | Moderate | Study limitations |
| Met/Flu vs. Orlistat | Indirect | Low | Study limitations |
| Met/Flu vs. PHEN/TPM | Indirect | Low | Study limitations |
| Met/Flu vs. Semaglutide | Indirect | Low | Study limitations |
| Met/Flu vs. Sibutramine | Indirect | Low | Study limitations |
| Metformin vs. Orlistat | Indirect | Low | Study limitations |
| Metformin vs. PHEN/TPM | Indirect | Low | Study limitations |
| Metformin vs. Semaglutide | Indirect | Low | Study limitations |
| Metformin vs. Sibutramine | Indirect | Low | Study limitations |
| Orlistat vs. PHEN/TPM | Indirect | Low | Study limitations |
| Orlistat vs. Semaglutide | Indirect | Moderate | Study limitations |
| Orlistat vs. Sibutramine | Indirect | Moderate | Study limitations |
| PHEN/TPM vs. Semaglutide | Indirect | Very low | Study limitations, Serious Imprecision |
| PHEN/TPM vs. Sibutramine | Indirect | Very low | Study limitations, Serious Imprecision |
| Semaglutide vs. Sibutramine | Indirect | Moderate | Study limitations |

Supplemental Table 20f. CINeMA ratings for all comparisons of TC

| **Comparison** | **Nature**  **of**  **evidence** | **Confidence**  **level** | **Downgrading** |
| --- | --- | --- | --- |
| Exenatide vs. Control | Mixed | Very low | Study limitations, Reporting bias, Serious Imprecision |
| Metformin vs.Control | Mixed | Very low | Study limitations, Reporting bias, Serious Imprecision |
| Orlistat vs. Control | Mixed | Moderate | Study limitations, Reporting bias |
| Sibutramine vs. Control | Mixed | Very low | Study limitations, Reporting bias, Serious Imprecision |
| Topiramate vs. Control | Mixed | Very low | Study limitations, Reporting bias, Serious Imprecision |
| Exenatide vs. Metformin | Indirect | Very low | Study limitations, Reporting bias, Serious Imprecision |
| Exenatide vs.Orlistat | Indirect | Moderate | Study limitations, Reporting bias |
| Exenatide vs. Sibutramine | Indirect | Very low | Study limitations, Reporting bias, Serious Imprecision |
| Exenatide vs. Topiramate | Indirect | Very low | Study limitations, Reporting bias, Serious Imprecision |
| Metformin vs. Orlistat | Indirect | Moderate | Study limitations, Reporting bias |
| Metformin vs. Sibutramine | Indirect | Very low | Study limitations, Reporting bias, Serious Imprecision |
| Metformin vs. Topiramate | Indirect | Very low | Study limitations, Reporting bias, Serious Imprecision |
| Orlistat vs. Sibutramine | Indirect | Very low | Study limitations, Reporting bias, Serious Imprecision |
| Orlistat vs.Topiramate | Indirect | Very low | Study limitations, Reporting bias, Serious Imprecision |
| Sibutramine vs. Topiramate | Indirect | Very low | Study limitations, Reporting bias, Serious Imprecision |

Supplemental Table 20g. CINeMA ratings for all comparisons of LDL-C

| **Comparison** | **Nature**  **of**  **evidence** | **Confidence**  **level** | **Downgrading** |
| --- | --- | --- | --- |
| Exenatide vs. Control | Mixed | Very low | Study limitations, Serious Imprecision |
| Metformin vs.Control | Mixed | Very low | Study limitations, Serious Imprecision |
| Orlistat vs. Control | Mixed | Very low | Study limitations, Serious Imprecision |
| Sibutramine vs. Control | Mixed | Very low | Study limitations, Serious Imprecision |
| Topiramate vs. Control | Mixed | Very low | Study limitations, Serious Imprecision |
| Exenatide vs. Metformin | Indirect | Very low | Study limitations, Serious Imprecision |
| Exenatide vs. Orlistat | Indirect | Very low | Study limitations, Serious Imprecision |
| Exenatide vs. Sibutramine | Indirect | Very low | Study limitations, Serious Imprecision |
| Exenatide vs. Topiramate | Indirect | Very low | Study limitations, Serious Imprecision |
| Metformin vs. Orlistat | Indirect | Very low | Study limitations, Serious Imprecision |
| Metformin vs. Sibutramine | Indirect | Very low | Study limitations, Serious Imprecision |
| Metformin vs. Topiramate | Indirect | Very low | Study limitations, Serious Imprecision |
| Orlistat vs. Sibutramine | Indirect | Very low | Study limitations, Serious Imprecision |
| Orlistat vs. Topiramate | Indirect | Very low | Study limitations, Serious Imprecision |
| Sibutramine vs. Topiramate | Indirect | Very low | Study limitations, Serious Imprecision |

Supplemental Table 20h. CINeMA ratings for all comparisons of HDL-C

| **Comparison** | **Nature**  **of**  **evidence** | **Confidence**  **level** | **Downgrading** |
| --- | --- | --- | --- |
| Exenatide vs. Control | Mixed | Very low | Study limitations, Serious Imprecision |
| Metformin vs. Control | Mixed | Very low | Serious Imprecision |
| Orlistat vs. Control | Mixed | Very low | Study limitations, Serious Imprecision |
| Sibutramine vs. Control | Mixed | Low | Study limitations |
| Topiramate vs. Control | Mixed | Very low | Study limitations |
| Exenatide vs. Metformin | Indirect | Very low | Study limitations, Serious Imprecision |
| Exenatide vs. Orlistat | Indirect | Very low | Study limitations, Serious Imprecision |
| Exenatide vs. Sibutramine | Indirect | Very low | Study limitations, Serious Imprecision |
| Exenatide vs. Topiramate | Indirect | Very low | Study limitations, Serious Imprecision |
| Metformin vs. Orlistat | Indirect | Very low | Study limitations, Serious Imprecision |
| Metformin vs. Sibutramine | Indirect | Low | Study limitations, Serious Imprecision |
| Metformin vs. Topiramate | Indirect | Very low | Study limitations, Serious Imprecision |
| Orlistat vs. Sibutramine | Indirect | Very low | Study limitations, Serious Imprecision |
| Orlistat vs. Topiramate | Indirect | Very low | Study limitations, Serious Imprecision |
| Sibutramine vs. Topiramate | Indirect | Very low | Study limitations, Serious Imprecision |

Supplemental Table 20i. CINeMA ratings for all comparisons of TG

| **Comparison** | **Nature**  **of**  **evidence** | **Confidence**  **level** | **Downgrading** |
| --- | --- | --- | --- |
| Exenatide vs. Control | Mixed | Very low | Study limitations, Reporting bias, Serious Imprecision |
| Metformin vs. Control | Mixed | Very low | Study limitations, Reporting bias, Serious Imprecision |
| Orlistat vs. Control | Mixed | Very low | Study limitations, Reporting bias, Serious Imprecision |
| Sibutramine vs. Control | Mixed | Very low | Study limitations, Reporting bias |
| Topiramate vs. Control | Mixed | Very low | Study limitations, Reporting bias |
| Exenatide vs. Metformin | Indirect | Very low | Study limitations, Reporting bias, Serious Imprecision |
| Exenatide vs. Orlistat | Indirect | Moderate | Study limitations, Reporting bias, Serious Imprecision |
| Exenatide vs. Sibutramine | Indirect | Very low | Study limitations, Reporting bias, Serious Imprecision |
| Exenatide vs. Topiramate | Indirect | Very low | Study limitations, Reporting bias, Serious Imprecision |
| Metformin vs. Orlistat | Indirect | Very low | Study limitations, Reporting bias, Serious Imprecision |
| Metformin vs. Sibutramine | Indirect | Very low | Study limitations, Reporting bias |
| Metformin vs. Topiramate | Indirect | Very low | Study limitations, Reporting bias |
| Orlistat vs. Sibutramine | Indirect | Moderate | Study limitations, Reporting bias |
| Orlistat vs. Topiramate | Indirect | Very low | Study limitations, Reporting bias, Serious Imprecision |
| Sibutramine vs. Topiramate | Indirect | Very low | Study limitations, Reporting bias, Serious Imprecision |

Supplemental Table 20j. CINeMA ratings for all comparisons of FBG

| **Comparison** | **Nature**  **of**  **evidence** | **Confidence**  **level** | **Downgrading** |
| --- | --- | --- | --- |
| Exenatide vs. Control | Mixed | Very low | Study limitations, Serious Imprecision |
| Liraglutide vs. Control | Mixed | Low | Serious Imprecision |
| Metformin vs. Control | Mixed | Very low | Study limitations, Serious Imprecision |
| Orlistat vs. Control | Mixed | Moderate | Study limitations |
| Semaglutide vs. Control | Mixed | Moderate | Study limitations |
| Sibutramine vs. Control | Mixed | Very low | Study limitations, Serious Imprecision |
| Topiramate vs. Control | Mixed | Very low | Study limitations, Serious Imprecision |
| Exenatide vs. Liraglutide | Indirect | Very low | Study limitations, Serious Imprecision |
| Exenatide vs. Metformin | Indirect | Very low | Study limitations, Serious Imprecision |
| Exenatide vs. Orlistat | Indirect | Very low | Study limitations, Serious Imprecision |
| Exenatide vs. Semaglutide | Indirect | Very low | Study limitations, Serious Imprecision |
| Exenatide vs. Sibutramine | Indirect | Very low | Study limitations, Serious Imprecision |
| Exenatide vs. Topiramate | Indirect | Very low | Study limitations, Serious Imprecision |
| Liraglutide vs. Metformin | Indirect | Very low | Study limitations, Serious Imprecision |
| Liraglutide vs. Orlistat | Indirect | Very low | Study limitations, Serious Imprecision |
| Liraglutide vs. Semaglutide | Indirect | Very low | Study limitations, Serious Imprecision |
| Liraglutide vs. Sibutramine | Indirect | Very low | Study limitations, Serious Imprecision |
| Liraglutide vs. Topiramate | Indirect | Very low | Study limitations, Serious Imprecision |
| Metformin vs. Orlistat | Indirect | Moderate | Study limitations |
| Metformin vs. Semaglutide | Indirect | Very low | Study limitations, Serious Imprecision |
| Metformin vs. Sibutramine | Indirect | Very low | Study limitations, Serious Imprecision |
| Metformin vs. Topiramate | Indirect | Very low | Study limitations, Serious Imprecision |
| Orlistat vs. Semaglutide | Indirect | Very low | Study limitations, Serious Imprecision |
| Orlistat vs. Sibutramine | Indirect | Very low | Study limitations, Serious Imprecision |
| Orlistat vs. Topiramate | Indirect | Very low | Study limitations, Serious Imprecision |
| Semaglutide vs. Sibutramine | Indirect | Very low | Study limitations, Serious Imprecision |
| Semaglutide vs. Topiramate | Indirect | Very low | Study limitations, Serious Imprecision |
| Sibutramine vs. Topiramate | Indirect | Very low | Study limitations, Serious Imprecision |

**Supplemental Table 20k. CINeMA ratings for all comparisons of FINS**

| **Comparison** | **Nature**  **of**  **evidence** | **Confidence**  **level** | **Downgrading** |
| --- | --- | --- | --- |
| Exenatide vs. Control | Mixed | Very low | Study limitations, Serious Imprecision |
| Metformin vs. Control | Mixed | Low | Study limitations |
| Orlistat vs. Control | Mixed | Very low | Study limitations, Serious Imprecision |
| Sibutramine vs. Control | Mixed | Very low | Study limitations, Serious Imprecision |
| Exenatide vs. Metformin | Indirect | Very low | Study limitations, Serious Imprecision |
| Exenatide vs. Orlistat | Indirect | Very low | Study limitations, Serious Imprecision |
| Exenatide vs. Sibutramine | Indirect | Very low | Study limitations, Serious Imprecision |
| Metformin vs. Orlistat | Indirect | Very low | Study limitations, Serious Imprecision |
| Metformin vs. Sibutramine | Indirect | Very low | Study limitations, Serious Imprecision |
| Orlistat vs. Sibutramine | Indirect | Very low | Study limitations, Serious Imprecision |

Supplemental Table 20l. CINeMA ratings for all comparisons of HOMA-IR

| **Comparison** | **Nature**  **of**  **evidence** | **Confidence**  **level** | **Downgrading** |
| --- | --- | --- | --- |
| Metformin vs. Control | Mixed | Very low | Study limitations, Reporting bias, Serious Imprecision |
| Orlistat vs. Control | Mixed | Very low | Study limitations, Reporting bias, Serious Imprecision |
| Sibutramine vs. Control | Mixed | Very low | Study limitations, Reporting bias |
| Metformin vs. Orlistat | Indirect | Very low | Study limitations, Reporting bias, Serious Imprecision |
| Metformin vs. Sibutramine | Indirect | Very low | Study limitations, Reporting bias |
| Orlistat vs. Sibutramine | Indirect | Very low | Study limitations, Reporting bias |

Supplemental Table 20m. CINeMA ratings for all comparisons of SBP

| **Comparison** | **Nature**  **of**  **evidence** | **Confidence**  **level** | **Downgrading** |
| --- | --- | --- | --- |
| Exenatide vs. Control | Mixed | Very low | Study limitations, Serious Imprecision |
| Liraglutide vs. Control | Mixed | Low | Serious Imprecision |
| Metformin vs. Control | Mixed | Very low | Study limitations, Serious Imprecision |
| PHEN/TPM vs. Control | Mixed | Very low | Study limitations, Serious Imprecision |
| Semaglutide vs. Control | Mixed | Low | Study limitations, Serious Imprecision |
| Sibutramine vs. Control | Mixed | Low | Study limitations |
| Topiramate vs. Control | Mixed | Very low | Study limitations, Serious Imprecision |
| Exenatide vs. Liraglutide | Indirect | Very low | Study limitations, Serious Imprecision |
| Exenatide vs. Metformin | Indirect | Very low | Study limitations, Serious Imprecision |
| Exenatide vs. PHEN/TPM | Indirect | Very low | Study limitations, Serious Imprecision |
| Exenatide vs. Semaglutide | Indirect | Very low | Study limitations, Serious Imprecision |
| Exenatide vs. Sibutramine | Indirect | Low | Study limitations |
| Exenatide vs. Topiramate | Indirect | Very low | Study limitations, Serious Imprecision |
| Liraglutide vs. Metformin | Indirect | Very low | Study limitations, Serious Imprecision |
| Liraglutide vs. PHEN/TPM | Indirect | Very low | Study limitations, Serious Imprecision |
| Liraglutide vs. Semaglutide | Indirect | Very low | Study limitations, Serious Imprecision |
| Liraglutide vs. Sibutramine | Indirect | Moderate | Study limitations |
| Liraglutide vs. Topiramate | Indirect | Very low | Study limitations, Serious Imprecision |
| Metformin vs. PHEN/TPM | Indirect | Very low | Study limitations, Serious Imprecision |
| Metformin vs. Semaglutide | Indirect | Very low | Study limitations, Serious Imprecision |
| Metformin vs. Sibutramine | Indirect | Low | Study limitations |
| Metformin vs. Topiramate | Indirect | Very low | Study limitations, Serious Imprecision |
| PHEN/TPM vs. Semaglutide | Indirect | Very low | Study limitations, Serious Imprecision |
| PHEN/TPM vs. Sibutramine | Indirect | Very low | Study limitations, Serious Imprecision |
| PHEN/TPM vs. Topiramate | Indirect | Very low | Study limitations, Serious Imprecision |
| Semaglutide vs. Sibutramine | Indirect | Very low | Study limitations, Serious Imprecision |
| Semaglutide vs. Topiramate | Indirect | Very low | Study limitations, Serious Imprecision |
| Sibutramine vs. Topiramate | Indirect | Very low | Study limitations, Serious Imprecision |

Supplemental Table 20n. CINeMA ratings for all comparisons of DBP

| **Comparison** | **Nature**  **of**  **evidence** | **Confidence**  **level** | **Downgrading** |
| --- | --- | --- | --- |
| Exenatide vs. Control | Mixed | Very low | Study limitations, Reporting bias, Serious Imprecision |
| Liraglutide vs. Control | Mixed | Very low | Reporting bias, Serious Imprecision |
| Metformin vs. Control | Mixed | Very low | Study limitations, Reporting bias, Serious Imprecision |
| PHEN/TPM vs. Control | Mixed | Moderate | Reporting bias |
| Semaglutide vs. Control | Mixed | Very low | Study limitations, Reporting bias, Serious Imprecision |
| Sibutramine vs. Control | Mixed | Very low | Study limitations, Reporting bias |
| Topiramate vs. Control | Mixed | Very low | Study limitations, Reporting bias, Serious Imprecision |
| Exenatide vs. Liraglutide | Indirect | Very low | Study limitations, Serious Imprecision |
| Exenatide vs. Metformin | Indirect | Very low | Study limitations, Reporting bias, Serious Imprecision |
| Exenatide vs. PHEN/TPM | Indirect | Very low | Study limitations, Reporting bias, Serious Imprecision |
| Exenatide vs. Semaglutide | Indirect | Very low | Study limitations, Reporting bias, Serious Imprecision |
| Exenatide vs. Sibutramine | Indirect | Very low | Study limitations, Reporting bias, Serious Imprecision |
| Exenatide vs. Topiramate | Indirect | Very low | Study limitations, Reporting bias, Serious Imprecision |
| Liraglutide vs. Metformin | Indirect | Very low | Study limitations, Reporting bias, Serious Imprecision |
| Liraglutide vs. PHEN/TPM | Indirect | Moderate | Reporting bias |
| Liraglutide vs. Semaglutide | Indirect | Very low | Study limitations, Reporting bias, Serious Imprecision |
| Liraglutide vs. Sibutramine | Indirect | Very low | Study limitations, Reporting bias, Serious Imprecision |
| Liraglutide vs. Topiramate | Indirect | Very low | Study limitations, Reporting bias, Serious Imprecision |
| Metformin vs. PHEN/TPM | Indirect | Moderate | Study limitations, Reporting bias |
| Metformin vs. Semaglutide | Indirect | Very low | Study limitations, Reporting bias, Serious Imprecision |
| Metformin vs. Sibutramine | Indirect | Very low | Study limitations, Reporting bias, Serious Imprecision |
| Metformin vs. Topiramate | Indirect | Very low | Study limitations, Reporting bias, Serious Imprecision |
| PHEN/TPM vs. Semaglutide | Indirect | Very low | Study limitations, Reporting bias, Serious Imprecision |
| PHEN/TPM vs. Sibutramine | Indirect | Moderate | Study limitations, Reporting bias |
| PHEN/TPM vs. Topiramate | Indirect | Very low | Study limitations, Reporting bias, Serious Imprecision |
| Semaglutide vs. Sibutramine | Indirect | Very low | Study limitations, Reporting bias, Serious Imprecision |
| Semaglutide vs. Topiramate | Indirect | Very low | Study limitations, Reporting bias, Serious Imprecision |
| Sibutramine vs. Topiramate | Indirect | Very low | Study limitations, Reporting bias, Serious Imprecision |

Supplemental Table 20o. CINeMA ratings for all comparisons of heart rate

| **Comparison** | **Nature**  **of**  **evidence** | **Confidence**  **level** | **Downgrading** |
| --- | --- | --- | --- |
| Exenatide vs. Control | Mixed | Very low | Study limitations, Reporting bias, Serious Imprecision |
| Liraglutide vs. Control | Mixed | Very low | Reporting bias, Serious Imprecision |
| Metformin vs. Control | Mixed | Very low | Study limitations, Reporting bias, Serious Imprecision |
| PHEN/TPM vs. Control | Mixed | Very low | Study limitations, Reporting bias, Serious Imprecision |
| Semaglutide vs. Control | Mixed | Very low | Study limitations, Reporting bias, Serious Imprecision |
| Sibutramine vs. Control | Mixed | Very low | Study limitations, Reporting bias |
| Topiramate vs. Control | Mixed | Very low | Study limitations, Reporting bias, Serious Imprecision |
| Exenatide vs. Liraglutide | Indirect | Very low | Study limitations, Reporting bias, Serious Imprecision |
| Exenatide vs. Metformin | Indirect | Very low | Study limitations, Reporting bias |
| Exenatide vs. PHEN/TPM | Indirect | Very low | Study limitations, Reporting bias, Serious Imprecision |
| Exenatide vs. Semaglutide | Indirect | Very low | Study limitations, Reporting bias, Serious Imprecision |
| Exenatide vs. Sibutramine | Indirect | Very low | Study limitations, Reporting bias, Serious Imprecision |
| Exenatide vs. Topiramate | Indirect | Very low | Study limitations, Reporting bias, Serious Imprecision |
| Liraglutide vs. Metformin | Indirect | Very low | Study limitations, Reporting bias, Serious Imprecision |
| Liraglutide vs. PHEN/TPM | Indirect | Very low | Study limitations, Reporting bias, Serious Imprecision |
| Liraglutide vs. Semaglutide | Indirect | Very low | Study limitations, Reporting bias, Serious Imprecision |
| Liraglutide vs. Sibutramine | Indirect | Very low | Study limitations, Reporting bias, Serious Imprecision |
| Liraglutide vs. Topiramate | Indirect | Very low | Study limitations, Reporting bias, Serious Imprecision |
| Metformin vs. PHEN/TPM | Indirect | Very low | Study limitations, Reporting bias, Serious Imprecision |
| Metformin vs. Semaglutide | Indirect | Very low | Study limitations, Reporting bias, Serious Imprecision |
| Metformin vs. Sibutramine | Indirect | Very low | Study limitations, Reporting bias |
| Metformin vs. Topiramate | Indirect | Very low | Study limitations, Reporting bias, Serious Imprecision |
| PHEN/TPM vs. Semaglutide | Indirect | Very low | Study limitations, Reporting bias, Serious Imprecision |
| PHEN/TPM vs. Sibutramine | Indirect | Very low | Study limitations, Reporting bias, Serious Imprecision |
| PHEN/TPM vs. Topiramate | Indirect | Very low | Study limitations, Reporting bias, Serious Imprecision |
| Semaglutide vs. Sibutramine | Indirect | Very low | Study limitations, Reporting bias, Serious Imprecision |
| Semaglutide vs. Topiramate | Indirect | Very low | Study limitations, Reporting bias, Serious Imprecision |
| Sibutramine vs. Topiramate | Indirect | Very low | Study limitations, Reporting bias, Serious Imprecision |

Supplemental Table 20p. CINeMA ratings for all comparisons of depression

| **Comparison** | **Nature**  **of**  **evidence** | **Confidence**  **level** | **Downgrading** |
| --- | --- | --- | --- |
| Liraglutide vs. Control | Mixed | Low | Serious Imprecision |
| Orlistat vs. Control | Mixed | Very low | Study limitations, Serious Imprecision |
| PHEN/TPM vs. Control | Mixed | Very low | Study limitations, Serious Imprecision |
| Semaglutide vs. Control | Mixed | Very low | Study limitations, Serious Imprecision |
| Sibutramine vs. Control | Mixed | Very low | Study limitations, Serious Imprecision |
| Topiramate vs. Control | Mixed | Very low | Study limitations, Serious Imprecision |
| Liraglutide vs. Orlistat | Indirect | Very low | Study limitations, Serious Imprecision |
| Liraglutide vs. PHEN/TPM | Indirect | Very low | Study limitations, Serious Imprecision |
| Liraglutide vs. Semaglutide | Indirect | Very low | Study limitations, Serious Imprecision |
| Liraglutide vs. Sibutramine | Indirect | Very low | Study limitations, Serious Imprecision |
| Liraglutide vs. Topiramate | Indirect | Very low | Study limitations, Serious Imprecision |
| Orlistat vs. PHEN/TPM | Indirect | Very low | Study limitations, Serious Imprecision |
| Orlistat vs. Semaglutide | Indirect | Very low | Study limitations, Serious Imprecision |
| Orlistat vs. Sibutramine | Indirect | Very low | Study limitations, Serious Imprecision |
| Orlistat vs. Topiramate | Indirect | Very low | Study limitations, Serious Imprecision |
| PHEN/TPM vs. Semaglutide | Indirect | Very low | Study limitations, Serious Imprecision |
| PHEN/TPM vs. Sibutramine | Indirect | Very low | Study limitations, Serious Imprecision |
| PHEN/TPM vs. Topiramate | Indirect | Very low | Study limitations, Serious Imprecision |
| Semaglutide vs. Sibutramine | Indirect | Very low | Study limitations, Serious Imprecision |
| Semaglutide vs. Topiramate | Indirect | Very low | Study limitations, Serious Imprecision |
| Sibutramine vs. Topiramate | Indirect | Very low | Study limitations, Serious Imprecision |

Supplemental Table 20q. CINeMA ratings for all comparisons of gastrointestinal disorders

| **Comparison** | **Nature**  **of**  **evidence** | **Confidence**  **level** | **Downgrading** |
| --- | --- | --- | --- |
| Exenatide vs. Control | Mixed | Very low | Study limitations, Serious Imprecision |
| Liraglutide vs. Control | Mixed | Low | Serious Imprecision |
| Metformin vs. Control | Mixed | Very low | Study limitations, Serious Imprecision |
| PHEN/TPM vs. Control | Mixed | Very low | Study limitations, Serious Imprecision |
| Semaglutide vs. Control | Mixed | Very low | Study limitations, Serious Imprecision |
| Exenatide vs. Liraglutide | Indirect | Very low | Study limitations, Serious Imprecision |
| Exenatide vs. Metformin | Indirect | Very low | Study limitations, Serious Imprecision |
| Exenatide vs. PHEN/TPM | Indirect | Very low | Study limitations, Serious Imprecision |
| Exenatide vs. Semaglutide | Indirect | Very low | Study limitations, Serious Imprecision |
| Liraglutide vs. Metformin | Indirect | Very low | Study limitations, Serious Imprecision |
| Liraglutide vs. PHEN/TPM | Indirect | Very low | Study limitations, Serious Imprecision |
| Liraglutide vs. Semaglutide | Indirect | Very low | Study limitations, Serious Imprecision |
| Metformin vs. PHEN/TPM | Indirect | Very low | Study limitations, Serious Imprecision |
| Metformin vs. Semaglutide | Indirect | Very low | Study limitations, Serious Imprecision |
| PHEN/TPM vs. Semaglutide | Indirect | Very low | Study limitations, Serious Imprecision |

Supplemental Table 20r. CINeMA ratings for all comparisons of SAE

| **Comparison** | **Nature**  **of**  **evidence** | **Confidence**  **level** | **Downgrading** |
| --- | --- | --- | --- |
| Exenatide vs. Control | Mixed | Very low | Study limitations, Serious Imprecision |
| Fluoxetine vs. Control | Mixed | Very low | Study limitations, Serious Imprecision |
| Liraglutide vs. Control | Mixed | Low | Serious Imprecision |
| Met/Flu vs. Control | Mixed | Very low | Study limitations, Serious Imprecision |
| Metformin vs. Control | Mixed | Very low | Study limitations, Serious Imprecision |
| Orlistat vs. Control | Mixed | Very low | Study limitations, Serious Imprecision |
| PHEN/TPM vs. Control | Mixed | Very low | Study limitations, Serious Imprecision |
| Semaglutide vs. Control | Mixed | Very low | Study limitations, Serious Imprecision |
| Sibutramine vs. Control | Mixed | Very low | Study limitations, Serious Imprecision |
| Topiramate vs. Control | Mixed | Very low | Study limitations, Serious Imprecision |
| Fluoxetine vs. Met/Flu | Mixed | Very low | Study limitations, Serious Imprecision |
| Fluoxetine vs. Metformin | Mixed | Very low | Study limitations, Serious Imprecision |
| Met/Flu vs. Metformin | Mixed | Very low | Study limitations, Serious Imprecision |
| Exenatide vs. Fluoxetine | Indirect | Very low | Study limitations, Serious Imprecision |
| Exenatide vs. Liraglutide | Indirect | Very low | Study limitations, Serious Imprecision |
| Exenatide vs. Met/Flu | Indirect | Very low | Study limitations, Serious Imprecision |
| Exenatide vs. Metformin | Indirect | Very low | Study limitations, Serious Imprecision |
| Exenatide vs. Orlistat | Indirect | Very low | Study limitations, Serious Imprecision |
| Exenatide vs. PHEN/TPM | Indirect | Very low | Study limitations, Serious Imprecision |
| Exenatide vs. Semaglutide | Indirect | Very low | Study limitations, Serious Imprecision |
| Exenatide vs. Sibutramine | Indirect | Very low | Study limitations, Serious Imprecision |
| Exenatide vs. Topiramate | Indirect | Very low | Study limitations, Serious Imprecision |
| Fluoxetine vs. Liraglutide | Indirect | Very low | Study limitations, Serious Imprecision |
| Fluoxetine vs. Orlistat | Indirect | Very low | Study limitations, Serious Imprecision |
| Fluoxetine vs. PHEN/TPM | Indirect | Very low | Study limitations, Serious Imprecision |
| Fluoxetine vs. Semaglutide | Indirect | Very low | Study limitations, Serious Imprecision |
| Fluoxetine vs. Sibutramine | Indirect | Very low | Study limitations, Serious Imprecision |
| Fluoxetine vs. Topiramate | Indirect | Very low | Study limitations, Serious Imprecision |
| Liraglutide vs. Met/Flu | Indirect | Very low | Study limitations, Serious Imprecision |
| Liraglutide vs. Metformin | Indirect | Very low | Study limitations, Serious Imprecision |
| Liraglutide vs. Orlistat | Indirect | Very low | Study limitations, Serious Imprecision |
| Liraglutide vs. PHEN/TPM | Indirect | Very low | Study limitations, Serious Imprecision |
| Liraglutide vs. Semaglutide | Indirect | Very low | Study limitations, Serious Imprecision |
| Liraglutide vs. Sibutramine | Indirect | Very low | Study limitations, Serious Imprecision |
| Liraglutide vs. Topiramate | Indirect | Very low | Study limitations, Serious Imprecision |
| Met/Flu vs. Orlistat | Indirect | Very low | Study limitations, Serious Imprecision |
| Met/Flu vs. PHEN/TPM | Indirect | Very low | Study limitations, Serious Imprecision |
| Met/Flu vs. Semaglutide | Indirect | Very low | Study limitations, Serious Imprecision |
| Met/Flu vs. Sibutramine | Indirect | Very low | Study limitations, Serious Imprecision |
| Met/Flu vs. Topiramate | Indirect | Very low | Study limitations, Serious Imprecision |
| Metformin vs. Orlistat | Indirect | Very low | Study limitations, Serious Imprecision |
| Metformin vs. PHEN/TPM | Indirect | Very low | Study limitations, Serious Imprecision |
| Metformin vs. Semaglutide | Indirect | Very low | Study limitations, Serious Imprecision |
| Metformin vs. Sibutramine | Indirect | Very low | Study limitations, Serious Imprecision |
| Metformin vs. Topiramate | Indirect | Very low | Study limitations, Serious Imprecision |
| Orlistat vs. PHEN/TPM | Indirect | Very low | Study limitations, Serious Imprecision |
| Orlistat vs. Semaglutide | Indirect | Very low | Study limitations, Serious Imprecision |
| Orlistat vs. Sibutramine | Indirect | Very low | Study limitations, Serious Imprecision |
| Orlistat vs. Topiramate | Indirect | Very low | Study limitations, Serious Imprecision |
| PHEN/TPM vs. Semaglutide | Indirect | Very low | Study limitations, Serious Imprecision |
| PHEN/TPM vs. Sibutramine | Indirect | Very low | Study limitations, Serious Imprecision |
| PHEN/TPM vs. Topiramate | Indirect | Very low | Study limitations, Serious Imprecision |
| Semaglutide vs. Sibutramine | Indirect | Very low | Study limitations, Serious Imprecision |
| Semaglutide vs. Topiramate | Indirect | Very low | Study limitations, Serious Imprecision |
| Sibutramine vs. Topiramate | Indirect | Very low | Study limitations, Serious Imprecision |

We present the evidence levels for all outcome comparisons. Comparison: names of the drugs compared. Nature of evidence: mixed (combination of direct and indirect evidence), indirect (only indirect evidence available). Confidence level: high, moderate, low, and very low. Downgrading: CINeMA items are responsible for downgrading. CINeMA (Confidence in Network Meta-analysis).

*Met/Flu* metformin/ fluoxetine, *PHEN/TPM* phentermine/topiramate, *Control* placebo or no treatment

**Appendix 15. References for included trials**

**Supplemental references**

1. Kelly AS, Bensignor MO, Hsia DS, Shoemaker AH, Shih W, Peterson C, *et al*. Phentermine/Topiramate for the Treatment of Adolescent Obesity. *NEJM Evid*. 2022;1(6).
2. Weghuber D, Barrett T, Barrientos-Pérez M, Gies I, Hesse D, Jeppesen OK, *et al*. Once-Weekly Semaglutide in Adolescents with Obesity. *N Engl J Med*. 2022;387(24):2245-57.
3. Weghuber D, Forslund A, Ahlström H, Alderborn A, Bergström K, Brunner S, *et al*. A 6-month randomized, double-blind, placebo-controlled trial of weekly exenatide in adolescents with obesity. *Pediatr Obes*. 2020;15(7):e12624.
4. Kelly AS, Rudser KD, Nathan BM, Fox CK, Metzig AM, Coombes BJ, *et al*. The effect of glucagon-like peptide-1 receptor agonist therapy on body mass index in adolescents with severe obesity: a randomized, placebo-controlled, clinical trial. *JAMA Pediatr*. 2013;167(4):355-60.
5. Fox CK, Clark JM, Rudser KD, Ryder JR, Gross AC, Nathan BM, *et al*. Exenatide for weight-loss maintenance in adolescents with severe obesity: A randomized, placebo-controlled trial. *Obesity (Silver Spring)*. 2022;30(5):1105-15.
6. Kelly AS, Auerbach P, Barrientos-Perez M, Gies I, Hale PM, Marcus C, *et al*. A Randomized, Controlled Trial of Liraglutide for Adolescents with Obesity. *N Engl J Med*. 2020;382(22):2117-28.
7. Fox CK, Kaizer AM, Rudser KD, Nathan BM, Gross AC, Sunni M, *et al*. Meal replacements followed by topiramate for the treatment of adolescent severe obesity: A pilot randomized controlled trial. *Obesity (Silver Spring)*. 2016;24(12):2553-61.
8. Atabek ME, Pirgon O. Use of metformin in obese adolescents with hyperinsulinemia: a 6-month, randomized, double-blind, placebo-controlled clinical trial. *J Pediatr Endocrinol Metab*. 2008;21(4):339-48.
9. Clarson CL, Mahmud FH, Baker JE, Clark HE, McKay WM, Schauteet VD, *et al*. Metformin in combination with structured lifestyle intervention improved body mass index in obese adolescents, but did not improve insulin resistance. *Endocrine*. 2009;36(1):141-6.
10. Freemark M. Liver dysfunction in paediatric obesity: a randomized, controlled trial of metformin. *Acta Paediatr*. 2007;96(9):1326-32.
11. Kendall D, Vail A, Amin R, Barrett T, Dimitri P, Ivison F, *et al*. Metformin in obese children and adolescents: the MOCA trial. *J Clin Endocrinol Metab*. 2013;98(1):322-9.
12. Mauras N, DelGiorno C, Hossain J, Bird K, Killen K, Merinbaum D, *et al*. Metformin use in children with obesity and normal glucose tolerance--effects on cardiovascular markers and intrahepatic fat. *J Pediatr Endocrinol Metab*. 2012;25(1-2):33-40.
13. Rezvanian H, Hashemipour M, Kelishadi R, Tavakoli N, Poursafa P. A randomized, triple masked, placebo-controlled clinical trial for controlling childhood obesity. *World J Pediatr*. 2010;6(4):317-22.
14. Wiegand S, l'Allemand D, Hübel H, Krude H, Bürmann M, Martus P, *et al*. Metformin and placebo therapy both improve weight management and fasting insulin in obese insulin-resistant adolescents: a prospective, placebo-controlled, randomized study. *Eur J Endocrinol*. 2010;163(4):585-92.
15. Wilson DM, Abrams SH, Aye T, Lee PD, Lenders C, Lustig RH, *et al*. Metformin extended release treatment of adolescent obesity: a 48-week randomized, double-blind, placebo-controlled trial with 48-week follow-up. *Arch Pediatr Adolesc Med*. 2010;164(2):116-23.
16. Yanovski JA, Krakoff J, Salaita CG, McDuffie JR, Kozlosky M, Sebring NG, *et al*. Effects of metformin on body weight and body composition in obese insulin-resistant children: a randomized clinical trial. *Diabetes*. 2011;60(2):477-85.
17. Pastor-Villaescusa B, Caballero-Villarraso J, Cañete MD, Hoyos R, Maldonado J, Bueno G, *et al*. Evaluation of differential effects of metformin treatment in obese children according to pubertal stage and genetic variations: study protocol for a randomized controlled trial. *Trials*. 2016;17(1):323.
18. Warnakulasuriya LS, Fernando MMA, Adikaram AVN, Thawfeek ARM, Anurasiri WL, Silva RR, *et al*. Metformin in the Management of Childhood Obesity: A Randomized Control Trial. *Child Obes*. 2018;14(8):553-65.
19. Burgert TS, Duran EJ, Goldberg-Gell R, Dziura J, Yeckel CW, Katz S, *et al*. Short-term metabolic and cardiovascular effects of metformin in markedly obese adolescents with normal glucose tolerance. *Pediatr Diabetes*. 2008;9(6):567-76.
20. Garibay-Nieto N, Queipo-García G, Alvarez F, Bustos M, Villanueva E, Ramírez F, *et al*. Effects of Conjugated Linoleic Acid and Metformin on Insulin Sensitivity in Obese Children: Randomized Clinical Trial. *J Clin Endocrinol Metab*. 2017;102(1):132-40.
21. Evia-Viscarra ML, Rodea-Montero ER, Apolinar-Jiménez E, Muñoz-Noriega N, García-Morales LM, Leaños-Pérez C, *et al*. The effects of metformin on inflammatory mediators in obese adolescents with insulin resistance: controlled randomized clinical trial. *J Pediatr Endocrinol Metab*. 2012;25(1-2):41-9.
22. Berkowitz RI, Wadden TA, Tershakovec AM, Cronquist JL. Behavior therapy and sibutramine for the treatment of adolescent obesity: a randomized controlled trial. *Jama*. 2003;289(14):1805-12.
23. Berkowitz RI, Fujioka K, Daniels SR, Hoppin AG, Owen S, Perry AC, *et al*. Effects of sibutramine treatment in obese adolescents: a randomized trial. *Ann Intern Med*. 2006;145(2):81-90.
24. García-Morales LM, Berber A, Macias-Lara CC, Lucio-Ortiz C, Del-Rio-Navarro BE, Dorantes-Alvárez LM. Use of sibutramine in obese mexican adolescents: a 6-month, randomized, double-blind, placebo-controlled, parallel-group trial. *Clin Ther*. 2006;28(5):770-82.
25. Van Mil EG, Westerterp KR, Kester AD, Delemarre-van de Waal HA, Gerver WJ, Saris WH. The effect of sibutramine on energy expenditure and body composition in obese adolescents. *J Clin Endocrinol Metab*. 2007;92(4):1409-14.
26. Chanoine JP, Hampl S, Jensen C, Boldrin M, Hauptman J. Effect of orlistat on weight and body composition in obese adolescents: a randomized controlled trial. *Jama*. 2005;293(23):2873-83.
27. Maahs D, de Serna DG, Kolotkin RL, Ralston S, Sandate J, Qualls C, *et al*. Randomized, double-blind, placebo-controlled trial of orlistat for weight loss in adolescents. *Endocr Pract*. 2006;12(1):18-28.
28. Safety and Efficacy of Xenical in Children and Adolescents With Obesity-Related Diseases. 2011; <https://clinicaltrials.gov/study/NCT00001723.> Updated December 18, 2012.
29. Ozkan B, Bereket A, Turan S, Keskin S. Addition of orlistat to conventional treatment in adolescents with severe obesity. *Eur J Pediatr*. 2004;163(12):738-41.
30. Zahmatkesh A, Sohouli MH, Shojaie S, Rohani P. The effect of orlistat in the treatment of non-alcoholic fatty liver in adolescents with overweight and obese. *European Journal of Pediatrics*. 2023.
